# Installation of -SO_2_F groups onto primary amides

**DOI:** 10.3762/bjoc.15.186

**Published:** 2019-08-09

**Authors:** Jing Liu, Shi-Meng Wang, Njud S Alharbi, Hua-Li Qin

**Affiliations:** 1 State Key Laboratory of Silicate Materials for Architectures; School of Chemistry, Chemical Engineering and Life Science, Wuhan University of Technology, 205 Luoshi Road, Wuhan 430070, China; 2 Biotechnology Research group, Deportment of Biological Sciences, Faculty of Science, King Abdulaziz University, Jeddah, Saudi Arabia

**Keywords:** *N*-fluorosulfonyl amides, primary amides, sulfuryl fluoride (SO_2_F_2_)

## Abstract

A protocol of SO_2_F_2_-mediated installation of sulfonyl fluoride onto primary amides has been developed providing a new portal to sulfur(VI) fluoride exchange (SuFEx) click chemistry. The generated molecules contain pharmaceutically important amide and -SO_2_F moieties for application in the discovery of new therapeutics.

## Introduction

**Figure 1:**
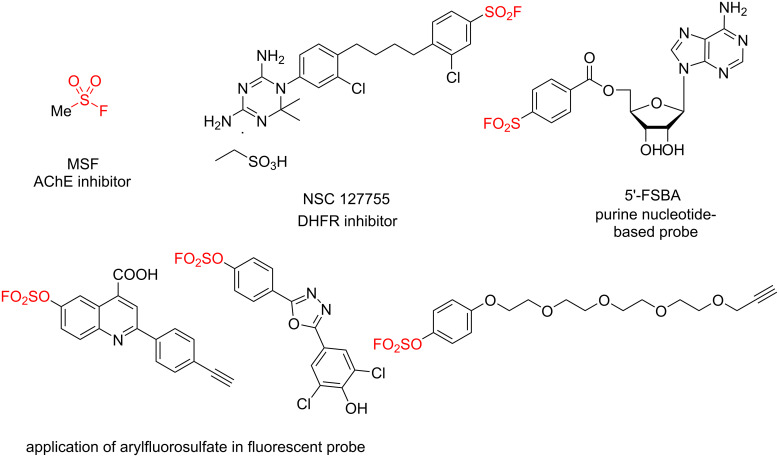
Representative sulfonyl fluoride compounds applied in medicinal chemistry and chemical biology.

Sulfur(VI) fluoride exchange (SuFEx) is a new class of click chemistry developed by Sharpless and co-workers in 2014, for creating molecular connections based on the unique stability–reactivity pattern of the S(VI)–F bond with reliability and efficiency, which has been widely applied in organic synthesis, chemical biology and drug discovery [1–19]. Among all the developed S(VI)–F species, sulfonyl fluoride (RSO_2_F) was specifically recognized as unique scaffold for covalent protein inhibitors and biological probes with the affinity-driven activation for forming covalent linkages with the amino acid residues of protein binding sites (Figure 1) [20]. The smallest member of this family, methyl sulfonyl fluoride (MSF), is known as a selective and irreversible inhibitor of acetylcholinesterase (AChE) [21–22]. The sulfonyl fluoride inhibitors NSC 127755 was found for specifically modifying tyrosine-31 of DHFR in chicken liver [23]. The nucleotide-derived probe 5’-(*para*-fluorosulfonylbenzoyl)adenosine (5’-FSBA) was used for labelling the second nucleotide binding site, the adenine nucleotide regulatory site [24]. In addition, aryl fluorosulfates have also been widely applied as sustainable alternative to aryl halides in coupling reactions and as potential covalent probes in protein profiling [14,25–28].

**Scheme 1:**
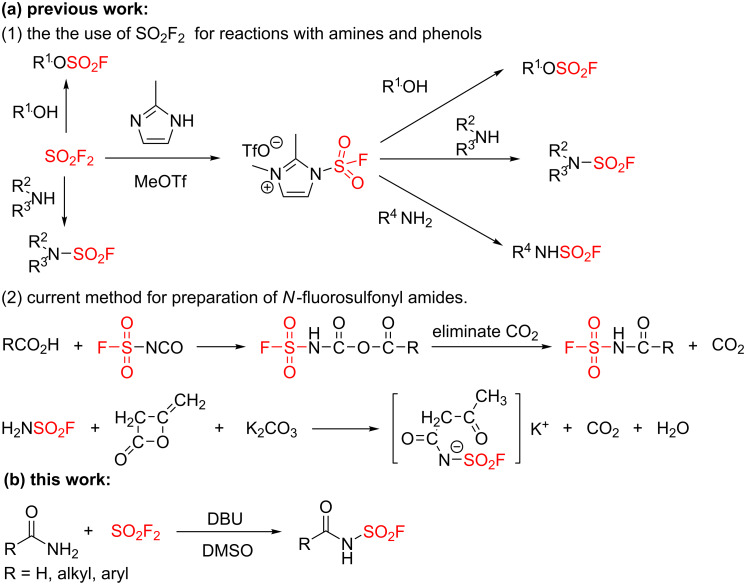
Synthesis background of *N*-fluorosulfonyl amides and fluorosulfates.

**Scheme 2:**
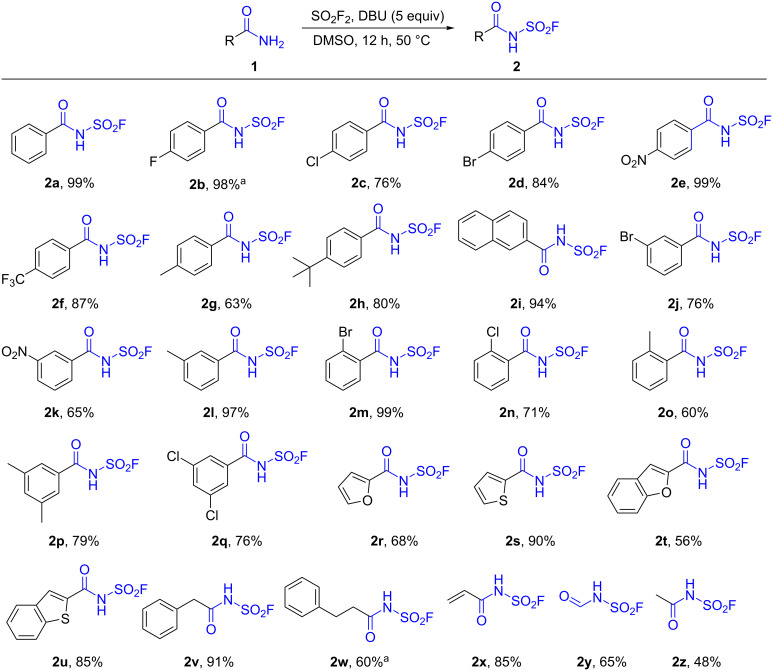
Screening of the substrate scope of amides. Reaction conditions: a mixture of amides **1** (1.0 mmol), DBU (5.0 mmol, 5.0 equiv), and DMSO (1.0 mL) was added to a reaction flask before SO_2_F_2_ was introduced into the stirred reaction mixture by slowly bubbling from a balloon, and the mixture was allowed to stir at 50 °C for 12 h. Isolated yields. ^a^50 °C, 18 h.

Phenols (or alcohols) and amines as the most common nucleophiles have been found to react with different S(VI) connectors (SO_2_F_2_, CH_2_=CH-SO_2_F, SOF_4_ etc.) to provide diversified sulfonyl fluoride derivatives. The reactions of phenols (or alcohols) with SO_2_F_2_ [29] or the fluorosulfuryl imidazolium salt were developed for mild and effective formation of the corresponding fluorosulfates to act as biological probes in chemical proteomics studies (Scheme 1, (1)) [1,30]. On the other hand, the reactions of aliphatic or aromatic amines with SO_2_F_2_ or the fluorosulfurylimidazolium salt have been achieved for assembly of *N*-sulfonyl fluorides [1,30], which have served as important active precursors for the development of noncovalent inhibitors (Scheme 1, (1)) [1,30–31]. Amides are the key connections in proteins, amides, and a vast number of synthetic structures, such as polymers, biologically active compounds and pharmaceutical products [32–35]. However, the installation of sulfonyl fluoride (SO_2_F) onto nitrogen atoms of amides has not been achieved, which, if accomplished, would provide a very important class of sulfonyl fluorides, namely, *N*-fluorosulfonyl amides, for the development of potential covalent inhibitors [1–24]. The Roesky group described a pioneering protocol for the synthesis of *N*-fluorosulfonyl amides from fluorosulfonyl isocyanate (Scheme 1, (2)) [36]. The available procedures for the preparation of *N*-fluorosulfonyl amides are very limited which relied on using either the isocyanate approach, or the amidosulfofluoride (FSO_2_NH_2_) (Scheme 1, (2)) [37–39]. Therefore, the development of a new method for the assembly of *N*-fluorosulfonyl amides from cheap and abundant reagent is highly desirable. Herein, we report the first, to the best of our knowledge, SO_2_F_2_-mediated *N*-fluorosulfonylation [40–42] of amides by using DBU as base for the constructions of a series *N*-fluorosulfonyl amides (Scheme 1b).

## Results and Discussions

Initially, benzamide (**1a**) was selected as model substrate to test the feasibility of this proposed *N*-fluorosulfonylation reaction in the presence of Cs_2_CO_3_ in DMSO under SO_2_F_2_ atmosphere (balloon) at 50 °C, and excitingly, the desired product benzoylsulfamoyl fluoride (**2a**) was obtained in 25% yield (Table 1, entry 1). Encouraged by this preliminary success, several common bases were evaluated, among which, 1,8-diazabicycloundec-7-ene (DBU) catalysed the proposed transformation most effectively to provide the desired product **2a** in nearly quantitative yield (Table 1, entries 2–7). Subsequently, different solvents were screened (Table 1, entries 5, 8–12) and DMSO was found to be the best option. Decreasing the temperature from 50 °C to 40 °C or even room temperature, or cutting down the amount of DBU to 4 equivalents resulted in decreased yields (Table 1, entries 13–15).

**Table 1:** Optimization of the reaction conditions.^a^

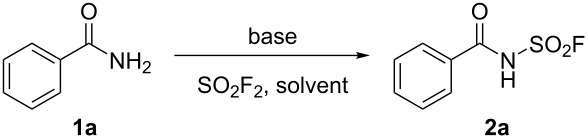				

Entry	Base	Solvent	Temp. (°C)	Yield (**2a**, %)^b^

1	Cs_2_CO_3_	DMSO	50	25
2	K_2_CO_3_	DMSO	50	13
3	KOH	DMSO	50	19
4	NaOH	DMSO	50	15
**5**	**DBU**	**DMSO**	**50**	**99**
6	Et_3_N	DMSO	50	–
7	DIPEA	DMSO	50	–
8	DBU	NMP	50	81
9	DBU	MeCN	50	75
10	DBU	toluene	50	87
11	DBU	dioxane	50	60
12	DBU	THF	50	79
13	DBU	DMSO	40	82
14	DBU	DMSO	R.T.	51
15^c^	DBU	DMSO	50	69

^a^Reaction conditions: benzamide (**1a**, 1.0 mmol, 1.0 equiv), DBU (5.0 equiv), and DMSO (1.0 mL) stirred with a SO_2_F_2_ balloon for 12 h. ^b^Isolated yield. ^c^4 equiv of DBU was used.

With the optimized conditions in hand, we next turned our efforts to investigate the scope of substrates. Under the standard conditions, a variety of substituted amides were examined which were smoothly converted to their corresponding substituted benzoylsulfamoyl fluoride derivatives (Scheme 2) in moderate to excellent isolated yields. Both electron-withdrawing groups, such as halogen atoms (**1b**–**d**, **1j**, **1m**, and **1n**), NO_2_ (**1e**, **1k**) and CF_3_ (**1f**), and electron-donating groups, such as Me (**1g**, **1l**, and **1o**), *tert*-butyl (**1h**) and 2-naphthyl (**1i**) on the aromatic rings, were well tolerated under the optimized conditions. It was worth noting that not only *para*- (**1b**–**h**) but also *meta*- (**1j**–**l**) and *ortho*- (**1m**–**o**) substituted benzamides afforded the desired products in generally good yields. Arylcarboxylic amides (**1p** and **1q**) bearing bis-substitutions also behaved well under the standard conditions. Heterocyclic aromatic carboxylic amides (**1r**–**u**) were well-tolerated and afforded the target products in 56–90% yields. In addition, alkyl carboxylic amides were also smoothly transformed into the corresponding products (**2v**–**z**). However, primary amides bearing an amino group or a phenolic hydroxy group were not successfully converted to the corresponding *N*-fluorosulfonyl amides and only a mixture of undesired products were observed.

**Scheme 3:**
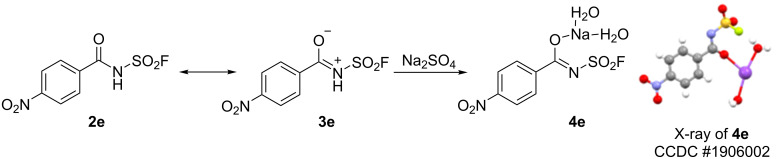
Amide resonance model and X-ray single crystal structure of **4e** (CCDC 1906002).

Interestingly, during the work-up process of drying **2e** with Na_2_SO_4_, a colourless crystal **4e** was observed and its structure was confirmed by XRD analysis (Scheme 3). We speculate that the tautomerism of amides [43] may occur in the reaction process and the tautomer **3e** could react with Na_2_SO_4_ to generate **4e**, which indicated that N–H connected with two electron-withdrawing groups (carbonyl, and SO_2_F) can behave as an acid to donate a proton for chemical transformations. This property of fluorosulfonyl amides **2** with nucleophilicity may attract significant attention for further applications.

**Scheme 4:**
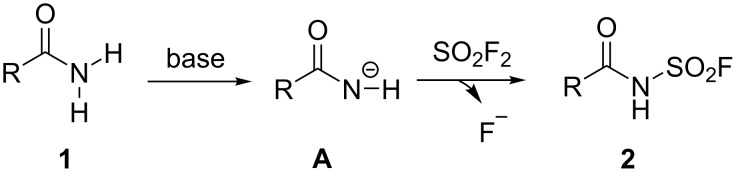
The proposed reaction mechanism.

As depicted in Scheme 4, a plausible reaction mechanism is proposed for SO_2_F_2_-mediated transformation of amides to *N*-fluorosulfonyl amides. The reaction was initiated by the deprotonation of amide **1** with the base (DBU) to generate an intermediate **A**, which subsequently went through a SuFEx process with SO_2_F_2_ to deliver the final product **2**.

## Conclusion

In conclusion, we have developed a novel method for *N*-fluorosulfonylation of amides. This simple, convenient, and mild protocol provides a portal to a class of novel sulfonyl fluorides for SuFEx click chemistry with great potential to be applied in the development of covalent inhibitors. Further studies of this class of molecules in chemical biology and drug discovery are underway in our laboratory.

## Supporting Information

File 1 Experimental part.

File 2 Crystallographic information file of **4e**.

File 3 Checkcif file of **4e**.
