# Supplementary material for: Installation of -SO2F groups onto primary amides
Source: Beilstein J Org Chem. 2019 Aug 9;15:1907–12. doi: 10.3762/bjoc.15.186 (PMC6693406; doi:10.3762/bjoc.15.186)
Supplement: File 1 — Experimental part. [file Beilstein_J_Org_Chem-15-1907-s001.pdf]

BEILSTEIN JOURNAL OF ORGANIC CHEMISTRY

Supporting Information

for

Installation of -SO_2F groups onto primary amides

Jing Liu, Shi-Meng Wang, Njud S. Alharbi and Hua-Li Qin

Beilstein J. Org. Chem. **2019,** *15,* 1907–1912. doi:10.3762/bjoc.15.186

Experimental part

License and Terms: This is a supporting information file under the terms of the Creative Commons Attribution License (http://creativecommons.org/licenses/by/4.0). Please note that the reuse, redistribution and reproduction in particular requires that the authors and source are credited.
The license is subject to the *Beilstein Journal of Organic Chemistry* terms and conditions: (https://www.beilstein-journals.org/bjoc)

Table of Contents

1. General information S2

2. Screening the optimized reaction conditions S3

3. General procedure for the synthesis of different amide S4

4. Procedure for the synthesis of **2a**–**z** S4

5. Procedure for the preparation of the crystal **4e** S4

6. Characterization of substrates S5

7. References S14

8. ^{1}H, ^{19}F, ^{13}C NMR spectra S15

9. Single crystal data of **4e** S41

1. General information

All reactions were carried out in dried glassware. All reagents were purchased from commercial sources and used without further purification. Unless otherwise specified, NMR spectra were recorded in $CDCl_3$ or DMSO-d_6 on a 500 MHz (for ^{1}H), 471 MHz (for ^{19}F), 126 MHz (for ^{13}C) spectrometer. All chemical shifts were reported in ppm relative to TMS (^{1}H NMR, 0 ppm) as internal standards. Some of the amides was prepared according to literature. Melting points of the products were measured on a micro melting point apparatus (SGW X-4) and are uncorrected. HRMS experiments were performed on a TOF-Q ESI or CI/EI instrument. The coupling constants are reported in Hertz (Hz). The following abbreviations are used to explain the multiplicities: s = singlet, d = doublet, t = triplet, q = quartet, m = multiplet.

2. Screening the optimized reaction conditions

Table S1 Screening the base[a]

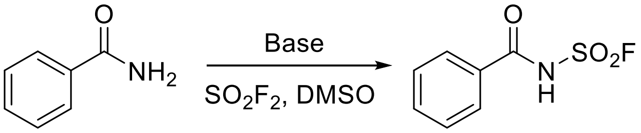

1a **2a**

Entry	Base	Solvent	Temperature (°C)	Yield (**2a**, %)[b]
1	Cs_2CO_3	DMSO	50	25
2	K_2CO_3	DMSO	50	13
3	KOH	DMSO	50	19
4	NaOH	DMSO	50	15
**5**	**DBU**	**DMSO**	**50**	**99**
6	Et_3N	DMSO	50	ND [c]
7	DIPEA	DMSO	50	ND

[a] Reaction conditions: Benzamide (**1a**, 1 mmol, 1 equiv), base (5 equiv), and DMSO (1 mL) stirred with a SO_2F_2 balloon at 50 °C for 12 h. [b] Isolated yields. [c] ND = Not Detected.

Table S2 Screening the solvents [a]

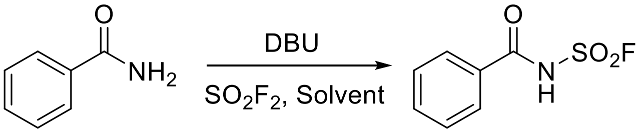

1a **2a**

Entry	Base	Solvent	Temperature (°C)	Yield (**2a**, %)[b]
**1**	**DBU**	**DMSO**	**50**	**99**
2	DBU	NMP	50	81
3	DBU	MeCN	50	75
4	DBU	Toluene	50	87
5	DBU	dioxane	50	60
6	DBU	THF	50	79

[a] Reaction conditions: Benzamide (**1a**, 1 mmol, 1 equiv), DBU (5 equiv), and solvent (1 mL) stirred with a SO_2F_2 balloon at 50 °C for 12 h. [b] Isolated yields.

Table S3: Screening the reaction temperature and DBU loading [a]

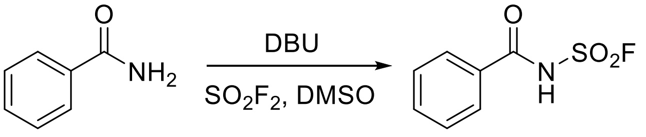

1a **2a**

Entry	Base	Solvent	Temperature (°C)	Yield (**2a**, %)[b]
**1**	**DBU (5 equiv)**	**DMSO**	**50**	**99**
2	DBU (5 equiv)	DMSO	40	82
3	DBU (5 equiv)	DMSO	R.T.	51
4	DBU (4 equiv)	DMSO	50	69

[a] Reaction conditions: Benzamide (**1a**, 1 mmol, 1 equiv), DBU (5 equiv), and DMSO (1 mL) stirred with a SO_2F_2 balloon for 12 h. [b] Isolated yields.

3. General procedure for the synthesis of different amides.

The amides of **1a–e**, **1j**, **1k**, **1m**, **1n**, **1m**, and **1x** – **z** were purchased from commercial sources and used without further purification. The amides of **1f**, **1g**, **1h**, **1i**, **1l**, and **1o** – **w** were prepared according to the literatures. [1, 2, 3, 4] All the homemade starting materials are identical to those reported regarding the ^{1}H and ^{13}C NMR and melting points (if applicable).

4. Procedure for the synthesis of 2a – z.

A mixture of amides **1** (1 mmol, 1 eq.), DBU (5 mmol, 5 equiv, 761.2 mg, 0.75 mL), and DMSO (1 mL) was allowed to stir at 50 °C under the atmosphere of a SO_2F_2 balloon overnight (monitored by TLC until complete consumption of amide starting materials).

The reaction mixture was quenched by 2 M HCl (10 mL). Then the reaction mixture was extracted with ethyl acetate (3 × 20 mL) and the extracts were washed with water, and concentrated to dryness under reduced pressure. The residue was purified by column chromatography on silica gel using pure ethyl acetate as eluent to give the desired product (**2**).

5. Procedure for the preparation of the crystal 4e

A mixture of amides **1e** (1 mmol, 1 equiv), DBU (5 mmol, 5 equiv, 761.2 mg, 0.75 mL), and DMSO (1 mL) was allowed to stir at 50 °C under the atmosphere of a SO_2F_2 balloon overnight (monitored by TLC until complete consumption of amide starting

materials). The reaction was quenched with 2 M HCl (10 mL), the resulting mixture was extracted with ethyl acetate (3 × 20 mL) and the extracts were washed with water. Then anhydrous Na_2SO_4 (10 mmol, 10 equiv) was added to the organic phase, which was filtered after being drying for 5 minutes, and the filtrate was concentrated to dryness under reduced pressure. The residue was purified by column chromatography on silica gel using pure ethyl acetate as eluent to give the desired product.

6. Characterization of substrates

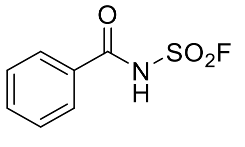

2a

Benzoylsulfamoyl fluoride (2a). Light orange oil (200.0 mg, isolated yield 99%). ^1H NMR (500 MHz, DMSO-d_6) δ 7.94 (d, J = 8.4 Hz, 2H), 7.46 (t, J = 7.4 Hz, 1H), 7.38 (t, J = 7.5 Hz, 2H). ^{19}F NMR (471 MHz, DMSO-d_6) δ 50.7 (s, 1F). ^{13}C NMR (126 MHz, DMSO-d_6) δ 170.4, 137.6, 130.9, 128.5, 127.8. HRMS ESI (m/z): $[M+H]^+$ calcd for $C_7H_7FNO_3S$: 204.0125, found: 204.0121.

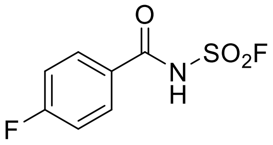

2b

(4-Fluorobenzoyl)sulfamoyl fluoride (2b). Light orange oil (216.8 mg, isolated yield 98%). ^1H NMR (500 MHz, DMSO-d_6) δ 7.99 (dd, J = 5.8, J = 8.8 Hz, 2H), 7.17 (t, J = 9.0 Hz, 2H). ^{19}F NMR (471 MHz, DMSO-d_6) δ 50.6 (s, 1F), －110.4- －110.5 (m, 1F). ^{13}C NMR (126 MHz, DMSO-d_6) δ 169.4, 164.0 (d, J = 248.0 Hz), 134.1, 131.1 (d, J = 9.1 Hz), 114.6 (d, J = 21.8 Hz). HRMS ESI (m/z): $[M+H]^+$ calcd for $C_7H_6F_2NO_3S$: 222,0031, found: 222.0028.

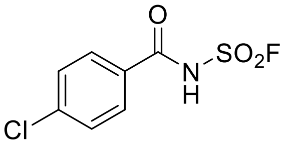

2c

(4-Chlorobenzoyl)sulfamoyl fluoride (2c). Yellow soild (181.1 mg, isolated yield 76%). ^{1}H NMR (500 MHz, DMSO-d$_6$) δ 7.94 (dd, J = 1.2, J = 8.4 Hz, 2H), 7.43 (d, J = 8.5 Hz, 2H). ^{19}F NMR (471 MHz, DMSO-d$_6$) δ 50.6 (s, 1F). ^{13}C NMR (126 MHz, DMSO-d$_6$) δ 167.0, 135.9, 133.1, 129.5, 128.4. Mp 138-139 °C. HRMS ESI (m/z): $[M+H]^+$ calcd for $C_7H_6ClFNO_3S$: 237.9735, found: 237.9731.

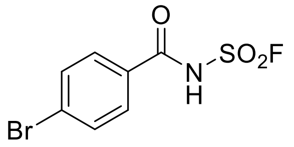

2d

(4-Bromobenzoyl)sulfamoyl fluoride (2d). Orange oil (236.0 mg, isolated yield 84%). ^{1}H NMR (500 MHz, DMSO-d$_6$) δ 7.87 (d, J = 8.5 Hz, 2H), 7.57 (d, J = 8.6 Hz, 2H). ^{19}F NMR (471 MHz, DMSO-d$_6$) δ 50.6 (s, 1F). ^{13}C NMR (126 MHz, DMSO-d$_6$) δ 169.6, 136.8 (d, J = 4.6 Hz), 130.9, 130.7, 124.8. HRMS ESI (m/z): $[M+H]^+$ calcd for $C_7H_6BrFNO_3S$: 281.9230, found: 281.9228.

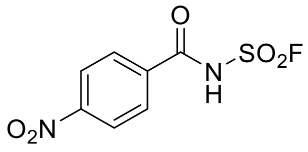

2e

(4-Nitrobenzoyl)sulfamoyl fluoride (2e). Yellow solid (245.5 mg, isolated yield 99%). ^{1}H NMR (500 MHz, DMSO-d$_6$) δ 8.23 (d, J = 8.9 Hz, 2H), 8.15 (d, J = 9.0 Hz, 2H). ^{19}F NMR (471 MHz, DMSO-d$_6$) δ 50.4 (s, 1F). ^{13}C NMR (126 MHz, DMSO-d$_6$) δ 168.3, 148.9, 143.3 (d, J = 3.6 Hz), 129.7, 123.1. Mp 117-119 °C. HRMS ESI (m/z): $[M+H]^+$ calcd for $C_7H_6FN_2O_5S$: 248.9976, found: 248.9972.

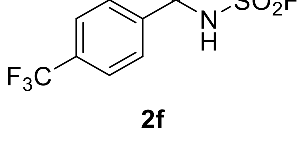

2f

(4-(Trifluoromethyl)benzoyl)sulfamoyl fluoride (2f). Light orange oil (236.9 mg, isolated yield 87%). ^{1}H NMR (500 MHz, DMSO-d$_6$) δ 8.14 (d, J = 8.1 Hz, 2H), 7.73 (d, J = 8.2 Hz, 2H). ^{19}F NMR (471 MHz, DMSO-d$_6$) δ 50.4 (s, 1F), - 61.4 (s, 3F). ^{13}C NMR (126 MHz, DMSO-d$_6$) δ 169.2, 141.4 (d, J = 3.6 Hz), 131.0 (q, J = 31.8 Hz),

129.3, 124.9 (q, J = 3.7 Hz), 124.3 (q, J = 272.5 Hz), 125.0-124.9 (m). HRMS ESI (m/z): $[M+H]^+$ calcd for $C_8H_6F_4NO_3S$: 271.9999, found: 271.9993.

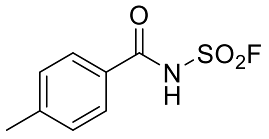

2g

(4-Methylbenzoyl)sulfamoyl fluoride (2g). Light orange oil (136.0 mg, isolated yield 63%). ^{1}H NMR (500 MHz, DMSO-d_6) δ 7.84 (d, J = 8.1 Hz, 2H), 7.17 (d, J = 8.1 Hz, 2H), 2.31 (s, 3H). ^{19}F NMR (471 MHz, DMSO-d_6) δ 50.8 (s, 1F). ^{13}C NMR (126 MHz, DMSO-d_6) δ 170.4 (d, J = 2.7 Hz), 140.5, 132.1, 130.1, 128.5 (d, J = 33.7 Hz), 21.0. HRMS ESI (m/z): $[M+H]^+$ calcd for $C_8H_9FNO_3S$: 218.0282, found: 218.0275.

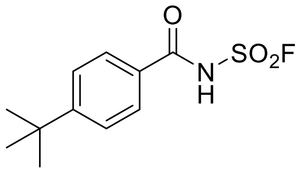

2h

(4-(*tert*-Butyl)benzoyl)sulfamoyl fluoride (2h). Light orange oil (206.2 mg, isolated yield 80%). ^{1}H NMR (500 MHz, DMSO-d_6) δ 7.88 (d, J = 8.2 Hz, 2H), 7.38 (d, J = 8.4 Hz, 2H), 1.28 (s, 9H). ^{19}F NMR (471 MHz, DMSO-d_6) δ 50.9 (s, 1F). ^{13}C NMR (126 MHz, DMSO-d_6) δ 170.4, 153.6, 135.0 (d, J = 4.5 Hz), 128.5, 124.5, 34.5, 31.1. HRMS ESI (m/z): $[M+H]^+$ calcd for $C_{11}H_{15}FNO_3S$: 260.0751, found: 260.0746.

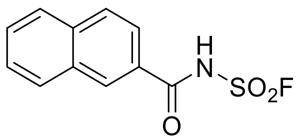

2i

(2-Naphthoyl)sulfamoyl fluoride (2i). Light orange oil (237.8 mg, isolated yield 94%). ^{1}H NMR (500 MHz, DMSO-d_6) δ 8.56 (s, 1H), 8.07-7.89 (m, 4H), 7.57-7.54 (m, 2H). ^{19}F NMR (471 MHz, DMSO-d_6) δ 51.1 (s, 1F). ^{13}C NMR (126 MHz, DMSO-d_6) δ 168.3, 134.5-134.4 (m), 132.3, 131.7, 128.9, 128.0-127.9 (m), 127.7-127.6 (m), 127.4-127.3 (m), 126.7, 126.34-126.32 (m), 124.5. HRMS ESI (m/z): $[M+H]^+$ calcd for $C_{11}H_9FNO_3S$: 254.0282, found: 254.0277.

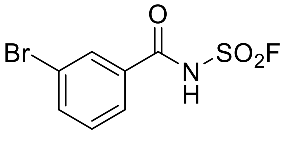

2j

(3-Bromobenzoyl)sulfamoyl fluoride (2j). Orange oil (215.1 mg, isolated yield 76%). ^{1}H NMR (500 MHz, DMSO-d_6) δ 8.01 (s, 1H), 7.88 (d, J = 7.6 Hz, 1H), 7.61 (d, J = 1.1 Hz, 1H), 7.33 (t, J = 7.9 Hz, 1H). ^{19}F NMR (471 MHz, DMSO-d_6) δ 50.5 (s, 1F). ^{13}C NMR (126 MHz, DMSO-d_6) δ 168.9, 139.9 (d, J = 4.6 Hz), 133.6, 131.2, 130.2, 127.5, 121.3. HRMS ESI (m/z): [M+H]$^+$ calcd for $C_7H_6BrFNO_3S$: 281.9230, found: 281.9228.

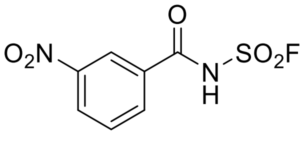

2k

(3-Nitrobenzoyl)sulfamoyl fluoride (2k). Yellow soild (160.6 mg, isolated yield 65%). ^{1}H NMR (500 MHz, DMSO-d_6) δ 8.67 (s, 1H), 8.33 (t, J = 7.8 Hz, 2H), 7.70 (t, J = 8.0 Hz, 1H). ^{19}F NMR (471 MHz, DMSO-d_6) δ 50.5 (s, 1F). ^{13}C NMR (126 MHz, DMSO-d_6) δ 167.9, 147.6, 139.2 (d, J = 4.5 Hz), 134.6, 129.7, 125.4, 122.9. Mp 94-96 °C. HRMS ESI (m/z): [M+H]$^+$ calcd for $C_7H_6FN_2O_5S$: 248.9976, found: 248.9972.

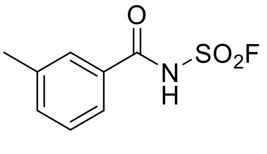

2l

(3-Methylbenzoyl)sulfamoyl fluoride (2l). Light orange oil (210.5 mg, isolated yield 97%). ^{1}H NMR (500 MHz, DMSO-d_6) δ 7.79 (s, 1H), 7.76-7.75 (m, 1H), 7.27-7.24 (m, 2H), 2.31 (s, 3H). ^{19}F NMR (471 MHz, DMSO-d_6) δ 50.8 (s, 1F). ^{13}C NMR (126 MHz, DMSO-d_6) δ 170.9, 137.7 (d, J = 3.6 Hz), 137.0, 131.6, 129.3, 127.8, 125.9, 20.8. HRMS ESI (m/z): [M+H]$^+$ calcd for $C_8H_9FNO_3S$: 218.0282, found: 218.0275.

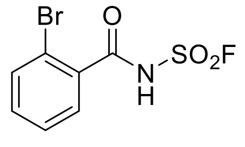

2m

(2-Bromobenzoyl)sulfamoyl fluoride (2m). Light orange oil (279.2 mg, isolated yield 99%). ^{1}H NMR (500 MHz, DMSO-d$_6$) δ 7.54 (d, J = 7.9 Hz, 1H), 7.51 (dd, J = 7.6, J = 1.5 Hz, 1H), 7.34 (t, J=7.4, 1H), 7.24 (td, J = 7.7, J = 1.5 Hz, 1H). ^{19}F NMR (471 MHz, DMSO-d$_6$) δ 50.5 (s, 1F). ^{13}C NMR (126 MHz, DMSO-d$_6$) δ 172.0, 141.1 (d, J = 4.5 Hz), 133.1, 130.3, 129.6, 127.3, 119.1. HRMS ESI (m/z): $[M+H]^+$ calcd for $C_7H_6BrFNO_3S$: 281.9230, found: 281.9228.

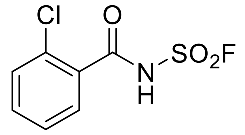

2n

(2-Chlorobenzoyl)sulfamoyl fluoride (2n). Light orange oil (169.1 mg, isolated yield 71%). ^{1}H NMR (500 MHz, DMSO-d$_6$) δ 7.54 (dd, J = 7.3, J = 1.6 Hz, 1H), 7.39-7.37 (m, 1H), 7.35-7.28 (m, 2H). ^{19}F NMR (471 MHz, DMSO-d$_6$) δ 50.5 (s, 1F). ^{13}C NMR (126 MHz, DMSO-d$_6$) δ 171.1, 139.1 (d, J = 4.5 Hz), 130.3, 130.1, 129.9, 129.6, 126.7. HRMS ESI (m/z): $[M+H]^+$ calcd for $C_7H_6ClFNO_3S$: 237.9735, found: 237.9731.

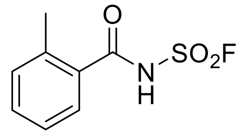

2o

(2-Methylbenzoyl)sulfamoyl fluoride (2o). Light orange oil (130.2 mg, isolated yield 60%). ^{1}H NMR (500 MHz, DMSO-d$_6$) δ 7.63 (d, J = 7.4 Hz, 1H), 7.24-7.22 (m, 1H), 7.16-7.13 (m, 2H), 2.44 (s, 3H). ^{19}F NMR (471 MHz, DMSO-d$_6$) δ 50.5 (s, 1F). ^{13}C NMR (126 MHz, DMSO-d$_6$) δ 173.5, 138.6 (d, J = 4.5 Hz), 136.6, 130.8, 129.1, 129.0, 125.2, 20.8. HRMS ESI (m/z): $[M+H]^+$ calcd for $C_8H_9FNO_3S$: 218.0282, found: 218.0275.

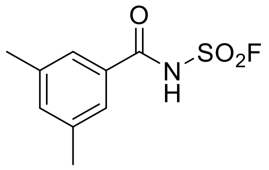

2p

(3,5-Dimethylbenzoyl)sulfamoyl fluoride (2p). Light orange oil (182.6 mg, isolated yield 79%). ^{1}H NMR (500 MHz, DMSO-d$_6$) δ 7.55 (s, 2H), 7.07 (s, 1H), 2.28 (s, 6H). ^{19}F NMR (471 MHz, DMSO-d$_6$) δ 50.8 (s, 1F). ^{13}C NMR (126 MHz, DMSO-d$_6$) δ 170.6, 136.6, 132.1, 126.4, 20.8. HRMS ESI (m/z): $[M+H]^+$ calcd for $C_9H_{11}FNO_3S$: 232.0438, found: 232.0432.

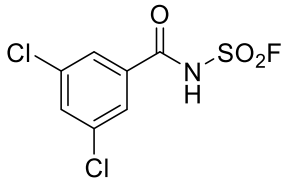

2q

(3,5-Dichlorobenzoyl)sulfamoyl fluoride (2q). Light orange oil (205.6 mg, isolated yield 76%). ^{1}H NMR (500 MHz, DMSO-d$_6$) δ 7.85 (d, J = 1.7 Hz, 2H), 7.61 (s, 1H). ^{19}F NMR (471 MHz, DMSO-d$_6$) δ 50.4 (s, 1F). ^{13}C NMR (126 MHz, DMSO-d$_6$) δ 167.6, 141.2 (d, J = 4.6 Hz), 134.0, 130.2, 127.1. HRMS ESI (m/z): $[M+H]^+$ calcd for $C_7H_5Cl_2FNO_3S$: 271.9346, found: 271.9341.

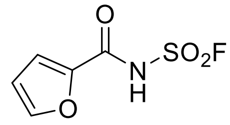

2r

(Furan-2-ylcarbonyl)sulfamoyl fluoride (2r). Light yellow soild (131.7 mg, isolated yield 68%). ^{1}H NMR (500 MHz, DMSO-d$_6$) δ 7.70 (s, 1H), 6.89 (d, J = 3.4 Hz, 1H), 6.50 (q, J = 1.7 Hz, 1H). ^{19}F NMR (471 MHz, DMSO-d$_6$) δ 51.8 (s, 1F). ^{13}C NMR (126 MHz, DMSO-d$_6$) δ 162.3, 145.0, 144.7, 114.0, 111.4. Mp 68-69 °C. HRMS ESI (m/z): $[M+H]^+$ calcd for $C_5H_5FNO_4S$: 193.9918, found: 193.9915.

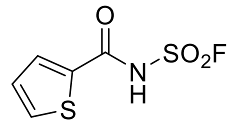

2s

(Thiophene-2-ylcarbonyl)sulfamoyl fluoride (2s). Yellow soild (188.1 mg, isolated yield 90%). ^{1}H NMR (500 MHz, DMSO-d$_6$) δ 7.60 (dd, J = 4.9, J = 1.1 Hz, 1H), 7.52 (d, J = 3.5 Hz, 1H), 7.05 (dd, J = 3.7, J = 4.9 Hz, 1H). ^{19}F NMR (471 MHz, DMSO-d$_6$) δ 56.2 (d, J = 5.7 Hz, 1F). ^{13}C NMR (126 MHz, DMSO-d$_6$) δ 163.0, 140.3, 131.0, 130.0, 128.8. HRMS ESI (m/z): [M+H]$^+$ calcd for $C_5H_5FNO_3S_2$: 209.9689, found: 209.9687.

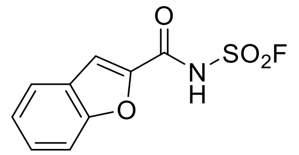

2t

(Benzofuran-2-ylcarbonyl)sulfamoyl fluoride (2t). Light orange oil (136.0 mg, isolated yield 56%). ^{1}H NMR (500 MHz, DMSO-d$_6$) δ 7.71 (d, J = 7.8 Hz, 1H), 7.61 (d, J = 8.4 Hz, 1H), 7.41-7.36 (m, 2H), 7.27 (t, J=7.5, 1H). ^{19}F NMR (471 MHz, DMSO-d$_6$) δ 51.6 (s, 1F). ^{13}C NMR (126 MHz, DMSO-d$_6$) δ 163.2, 154.7, 152.9, 127.6, 126.3, 123.4, 122.6, 111.8, 109.9. HRMS ESI (m/z): [M+H]$^+$ calcd for $C_9H_7FNO_4S$: 244.0074, found: 244.0070.

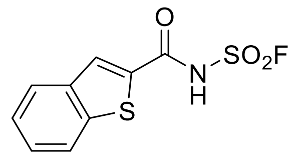

2u

(Benzo[*b*]thiophene-2-ylcarbonyl)sulfamoyl fluoride (2u). Light orange oil (220.4 mg, isolated yield 85%). ^{1}H NMR (500 MHz, DMSO-d$_6$) δ 7.93-7.89 (m, 3H), 7.41-7.35 (m, 2H). ^{19}F NMR (471 MHz, DMSO-d$_6$) δ 51.2 (s, 1F). ^{13}C NMR (126 MHz, DMSO-d$_6$) δ 166.3, 143.7 (d, J = 5.5 Hz), 141.1, 139.4, 126.7, 126.0, 125.2, 124.6, 122.8. HRMS ESI (m/z): [M+H]$^+$ calcd for $C_9H_7FNO_3S_2$: 259.9846, found: 259.9842.

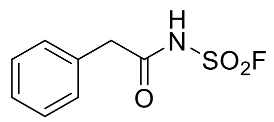

2v

(2-Phenylacetyl)sulfamoyl fluoride (2v). Light orange oil (197.9 mg, isolated yield 91%). ^{1}H NMR (500 MHz, DMSO-d$_6$) δ 7.28-7.25 (m, 2H), 7.22 (d, J = 6.9 Hz, 2H), 7.18 (t, J=7.1 Hz, 1H), 3.36 (s, 2H). ^{19}F NMR (471 MHz, DMSO-d$_6$) δ 50.2 (s, 1F). ^{13}C NMR (126 MHz, DMSO-d$_6$) δ 176.3, 137.4, 129.4, 128.0, 125.9, 46.1 (d, J = 4.5 Hz). HRMS ESI (m/z): [M+H]$^+$ calcd for $C_8H_9FNO_3S$: 218.0282, found: 218.0275.

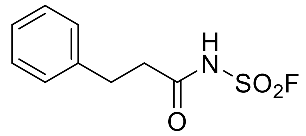

2w

(3-Phenylpropanoyl)sulfamoyl fluoride (2w). Light orange oil (138.6 mg, isolated yield 60%). ^{1}H NMR (500 MHz, DMSO-d$_6$) δ 7.26-7.23 (m, 2H), 7.19 (d, J = 6.9 Hz, 2H), 7.16-7.13 (m, 1H), 2.77 (t, J = 7.9 Hz, 2H), 2.35 (t, J = 7.9 Hz, 2H). ^{19}F NMR (471 MHz, DMSO-d$_6$) δ 50.8 (s, 1F). ^{13}C NMR (126 MHz, DMSO-d$_6$) δ 176.8, 141.8, 128.4 (d, J = 5.5 Hz), 126.9, 126.0, 31.2, 30.7. HRMS ESI (m/z): [M+H]$^+$ calcd for $C_9H_{11}FNO_3S$: 232.0438, found: 232.0432.

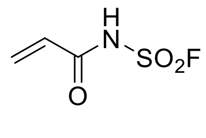

2x

Acryloylsulfamoyl fluoride (2x). Light orange oil (129.6 mg, isolated yield 85%). ^{1}H NMR (500 MHz, DMSO-d$_6$) δ 6.01-6.00 (m, 2H), 5.49 (dd, J = 4.6, J = 7.8 Hz, 1H). ^{19}F NMR (471 MHz, DMSO-d$_6$) δ 50.7 (s, 1F). ^{13}C NMR (126 MHz, DMSO-d$_6$) δ 170.3, 136.8 (d, J = 4.5 Hz), 124.7. HRMS ESI (m/z): [M+H]$^+$ calcd for $C_3H_5FNO_3S$: 153.9969, found:153.9961.

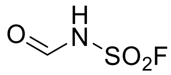

2y

Formylsulfamoyl fluoride (2y). Orange oil (82.5 mg, isolated yield 65%). ^{1}H NMR (500 MHz, DMSO-d_6) δ 8.92 (s, 1H). ^{19}F NMR (471 MHz, DMSO-d_6) δ 59.0 (s, 1F). ^{13}C NMR (126 MHz, DMSO-d_6) δ 170.4. HRMS ESI (m/z): $[M+H]^+$ calcd for CH_3FNO_3S: 127.9812, found: 127.9808.

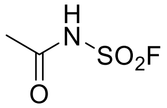

2z

Acetylsulfamoyl fluoride (2z). Light orange oil (67.7 mg, isolated yield 48%). ^{1}H NMR (500 MHz, DMSO-d_6) δ 1.99 (s, 3H). ^{19}F NMR (471 MHz, DMSO-d_6) δ 51.5 (s, 1F). ^{13}C NMR (126 MHz, DMSO-d_6) δ 171.0, 24.4. HRMS ESI (m/z): $[M+H]^+$ calcd for $C_2H_5FNO_3S$: 141.9969, found: 141.9965.

7. References

[1] Wu, Y.; Sun, P.; Zhang, K.; Yang, T.; Ya, H.; Lin, A. *J. Org. Chem.*, **2016**, *81*, 2166-2173.

[2] Lei, P.; Xu, Y.; Du, J.; Yang, X.-L.; Yuan, H.-Z.; Xu, G.-F.; Ling, Y. *Bioorg. Med. Chem. Lett*., **2016**, *26*, 2544-2546.

[3] Gaspari, P.; Banerjee, T.; Malachowski, W. P.; Muller, A. J.; Prendergast, G. C.; DuHadaway, J.; Bennett, S.; Donovan, A. M. *J. Med. Chem*., **2006**, *49*, 684-692.

[4] Guo, W.; Huang, J.; Wu, H.; Liu, T.; Luo, Z.; Jian, J.; Zeng, Z. *Org. Chem. Front.*, **2018**, *5*, 2950-2954.

8. ^{1}H, ^{19}F, ^{13}C NMR spectra

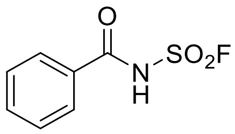

2a

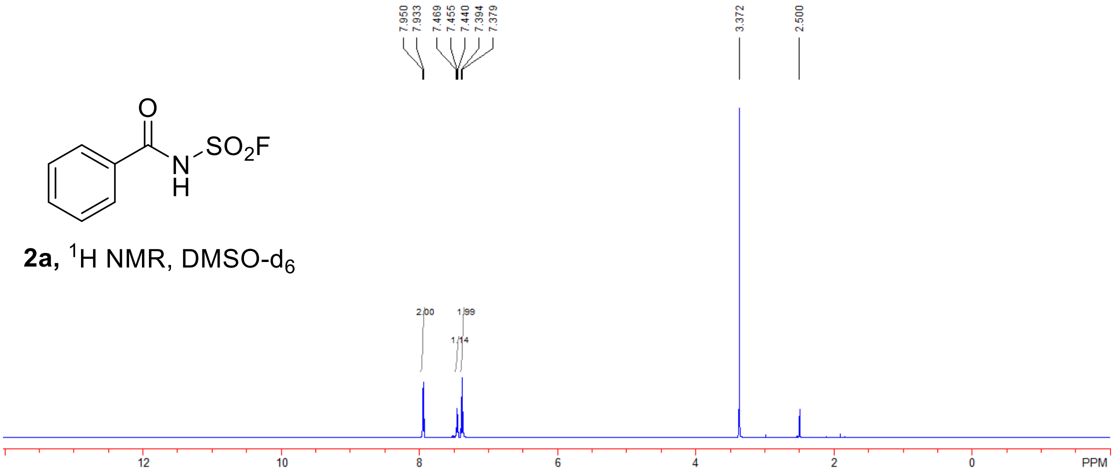

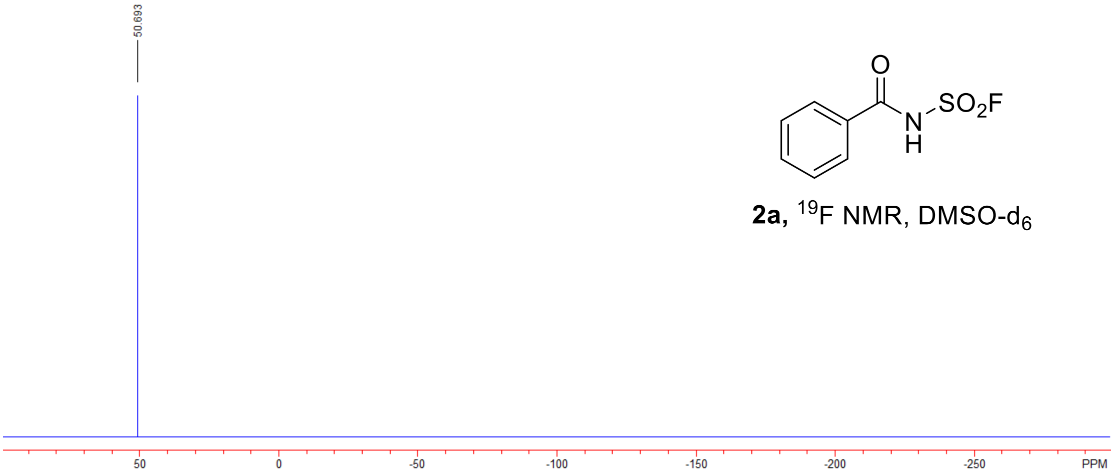

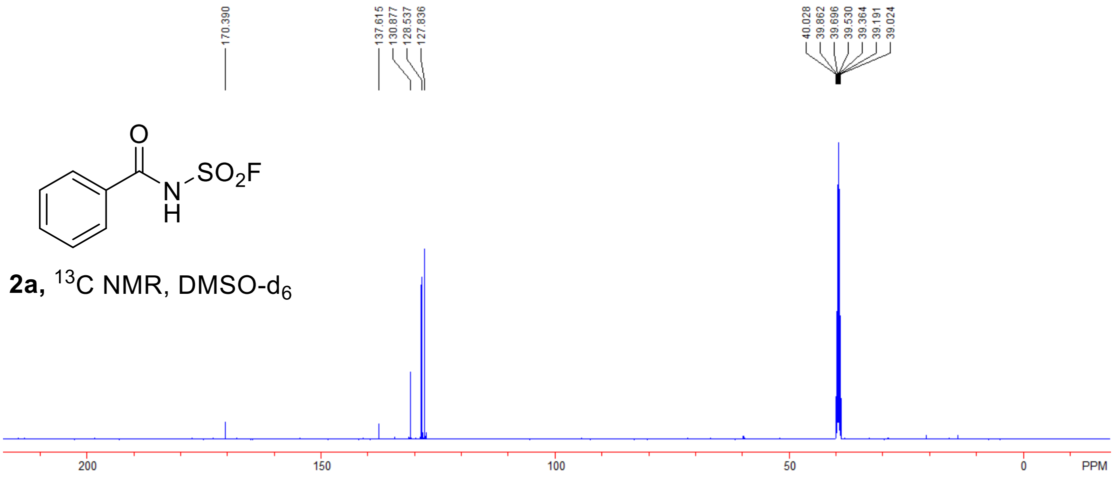

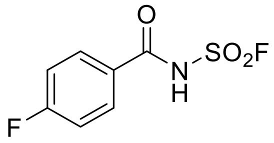

2b

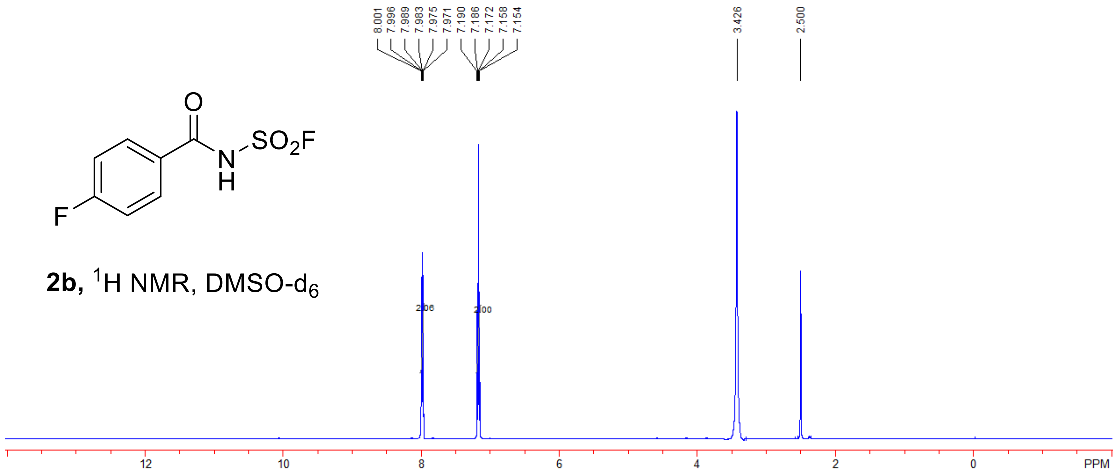
2b, ¹H NMR, DMSO-d6
8.001
7.996
7.989
7.983
7.975
7.971
7.190
7.186
7.172
7.158
7.154
3.426
2.500
PPM

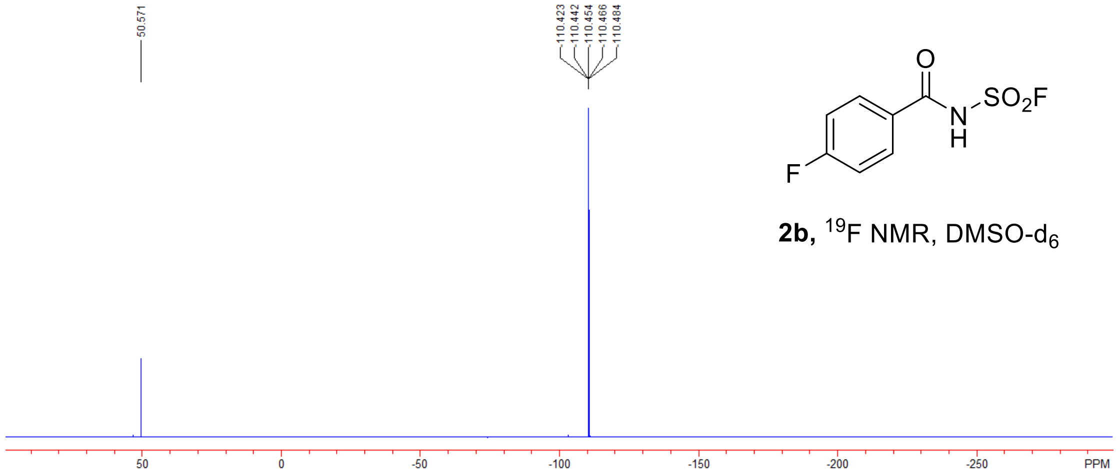
2b, ¹⁹F NMR, DMSO-d6
50.571
110.423
110.442
110.454
110.466
110.484
PPM

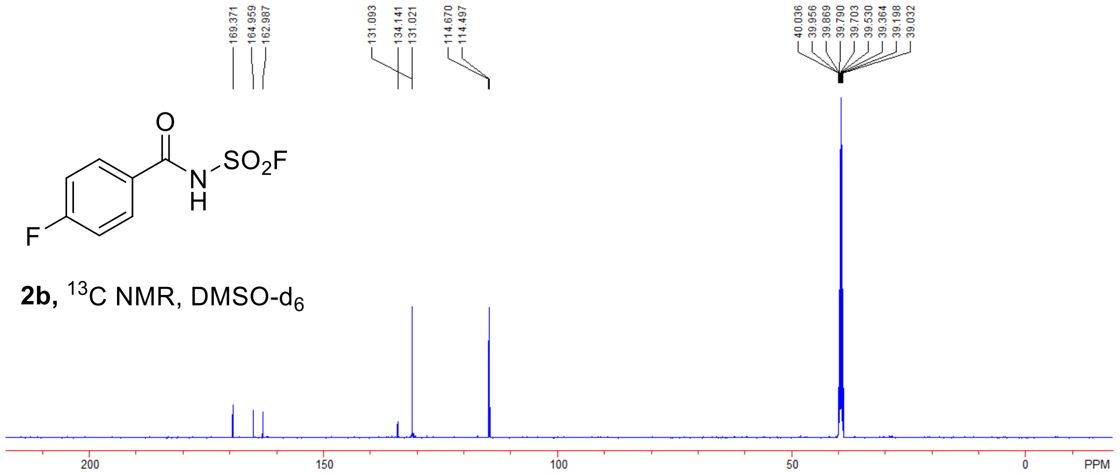
2b, ¹³C NMR, DMSO-d6
169.371
164.959
162.987
131.093
134.141
131.021
114.670
114.497
40.036
39.956
39.869
39.790
39.703
39.530
39.364
39.198
39.032
PPM

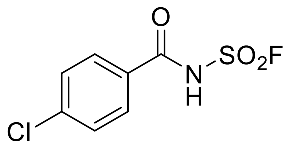
O
N
H
SO2F
Cl

2c

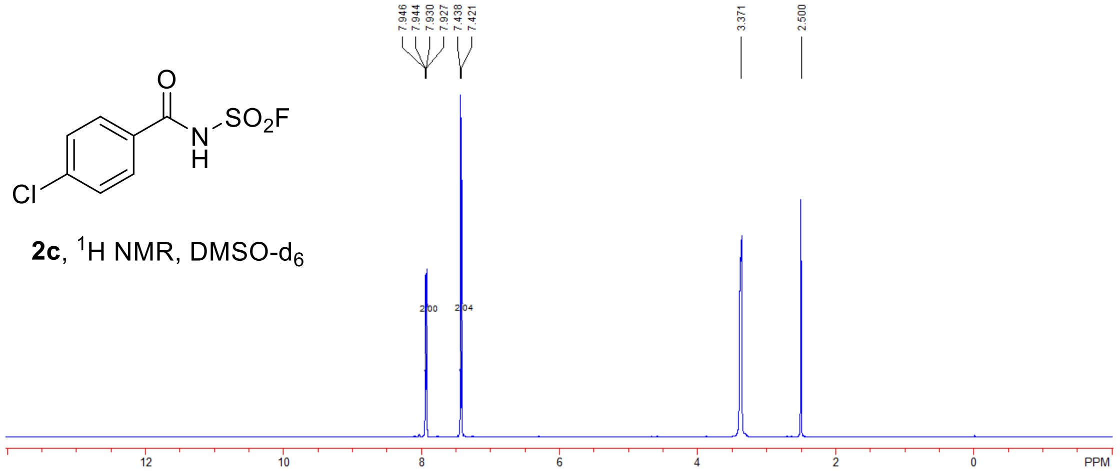
7.946
7.944
7.930
7.927
7.438
7.421
3.371
2.500
2c, 1H NMR, DMSO-d6
2.00
2.04
12
10
8
6
4
2
0
PPM

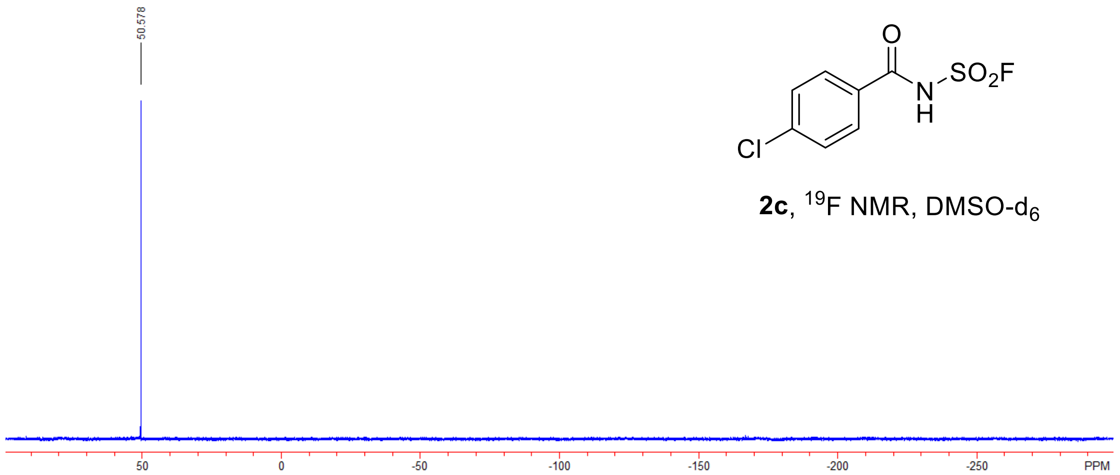
50.578
2c, 19F NMR, DMSO-d6
50
0
-50
-100
-150
-200
-250
PPM

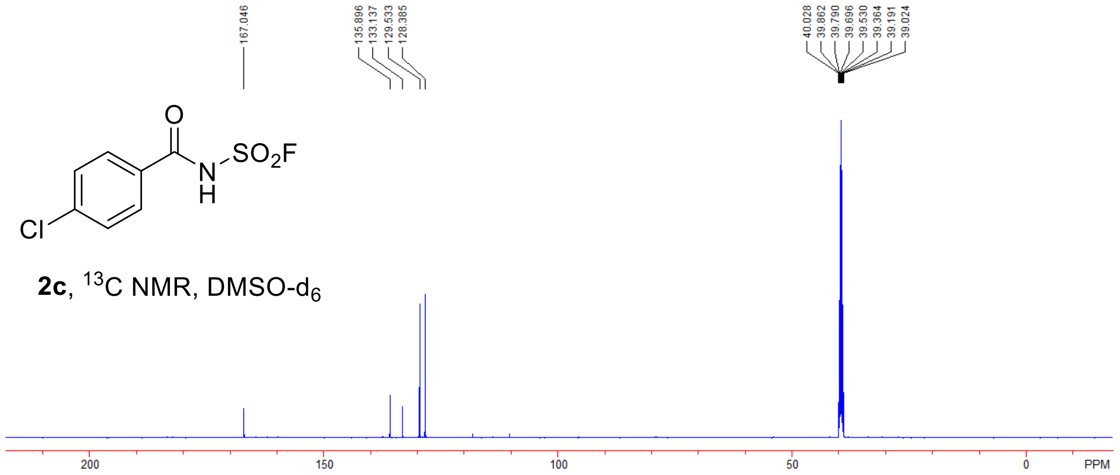
167.046
135.896
133.137
129.533
128.385
40.028
39.862
39.790
39.696
39.530
39.364
39.191
39.024
2c, 13C NMR, DMSO-d6
200
150
100
50
0
PPM

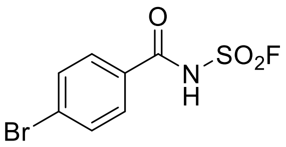
O
N
H
SO2F
Br

2d

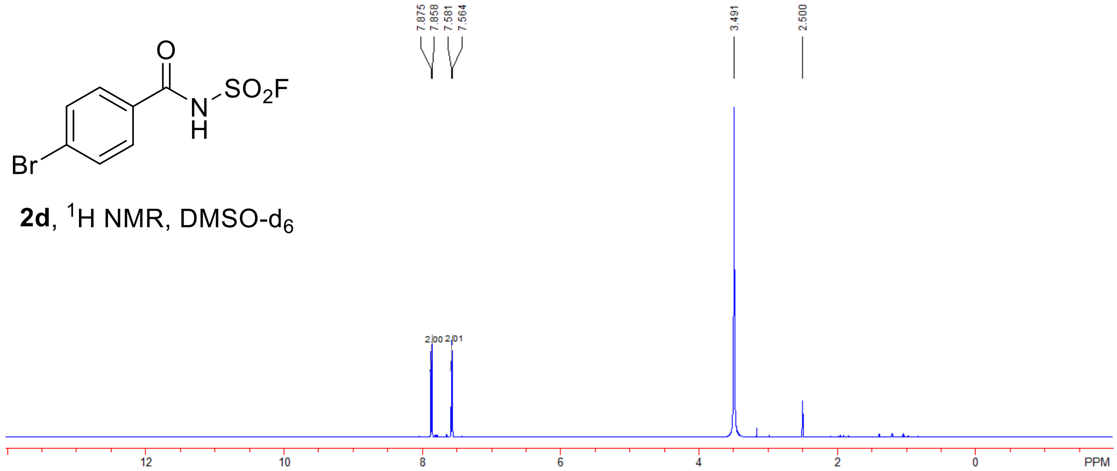
7.875
7.858
7.581
7.564
3.491
2.500
2.00
2.01
2d, 1H NMR, DMSO-d6
12
10
8
6
4
2
0
PPM

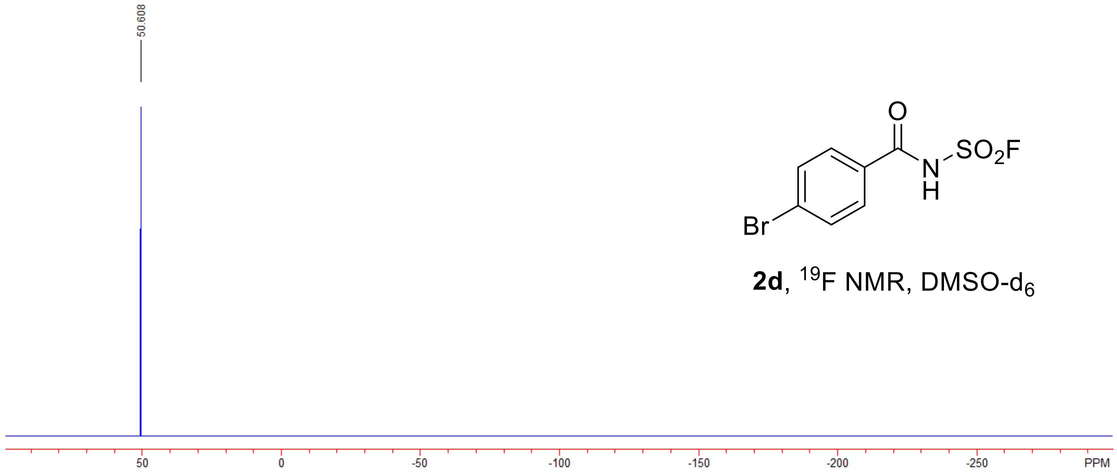
50.608
2d, 19F NMR, DMSO-d6
50
0
-50
-100
-150
-200
-250
PPM

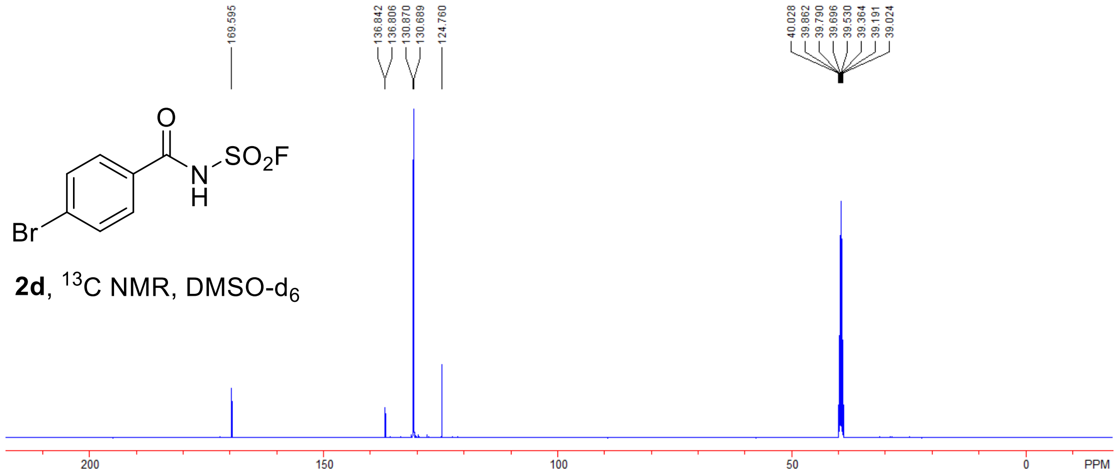
169.595
136.842
136.806
130.870
130.689
124.760
40.028
39.862
39.790
39.696
39.530
39.364
39.191
39.024
2d, 13C NMR, DMSO-d6
200
150
100
50
0
PPM

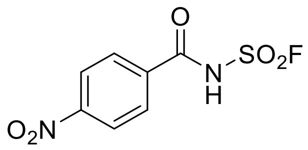
O
N
H
SO2F
O2N

2e

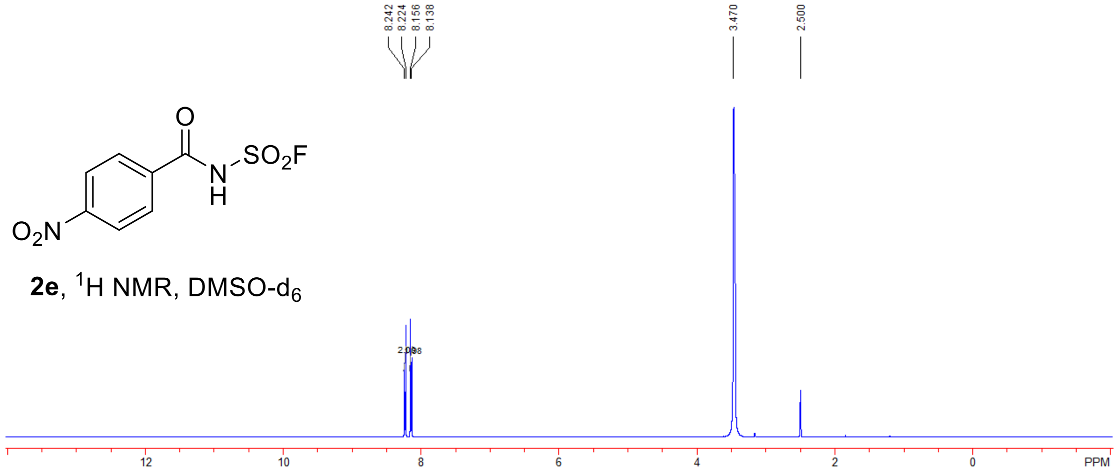
8.242
8.224
8.156
8.138
3.470
2.500
2e, 1H NMR, DMSO-d6
12
10
8
6
4
2
0
PPM

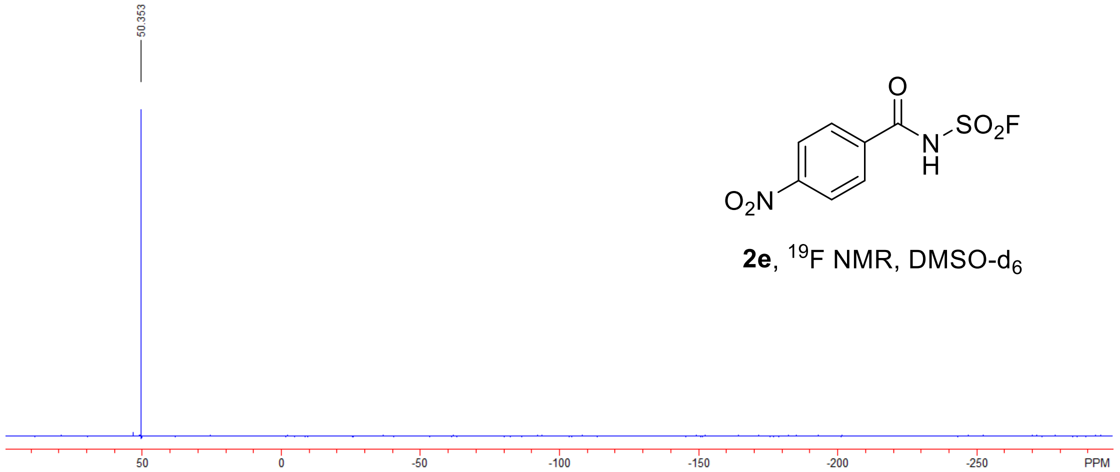
50.353
2e, 19F NMR, DMSO-d6
50
0
-50
-100
-150
-200
-250
PPM

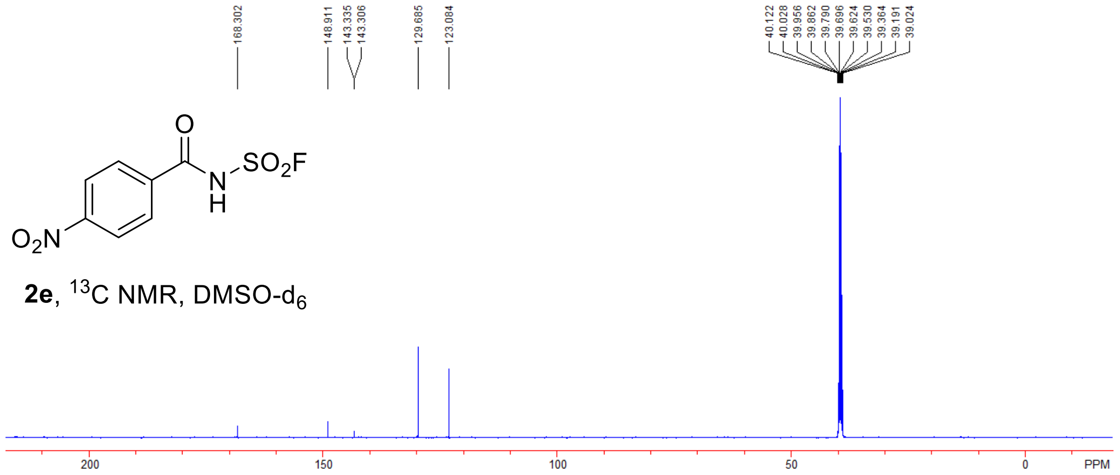
168.302
148.911
143.335
143.306
129.685
123.084
40.122
40.028
39.956
39.862
39.790
39.696
39.624
39.530
39.364
39.191
39.024
2e, 13C NMR, DMSO-d6
200
150
100
50
0
PPM

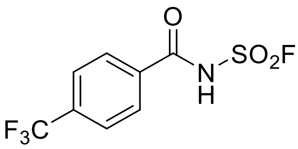
O
N
H
SO2F
F3C

2f

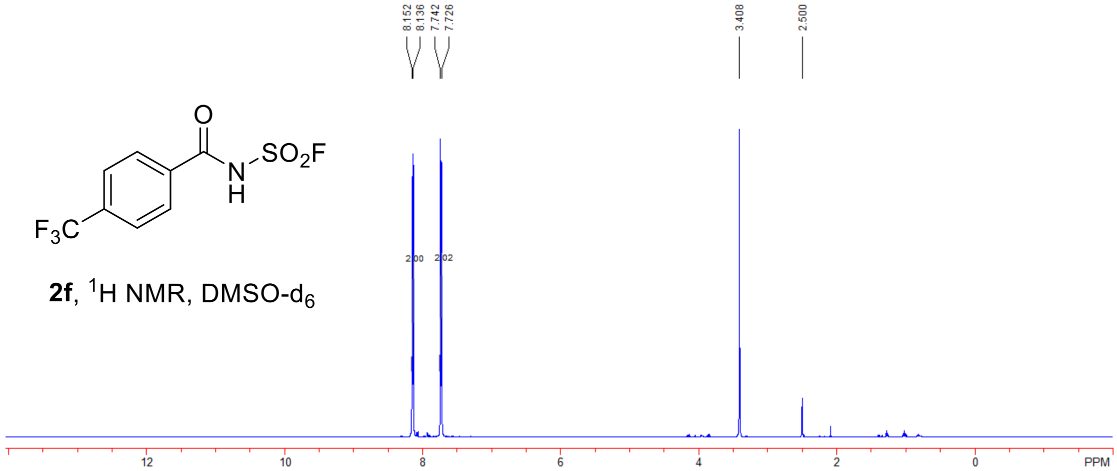
8.152
8.136
7.742
7.726
3.408
2.500
2.00
2.02
2f, 1H NMR, DMSO-d6
12
10
8
6
4
2
0
PPM

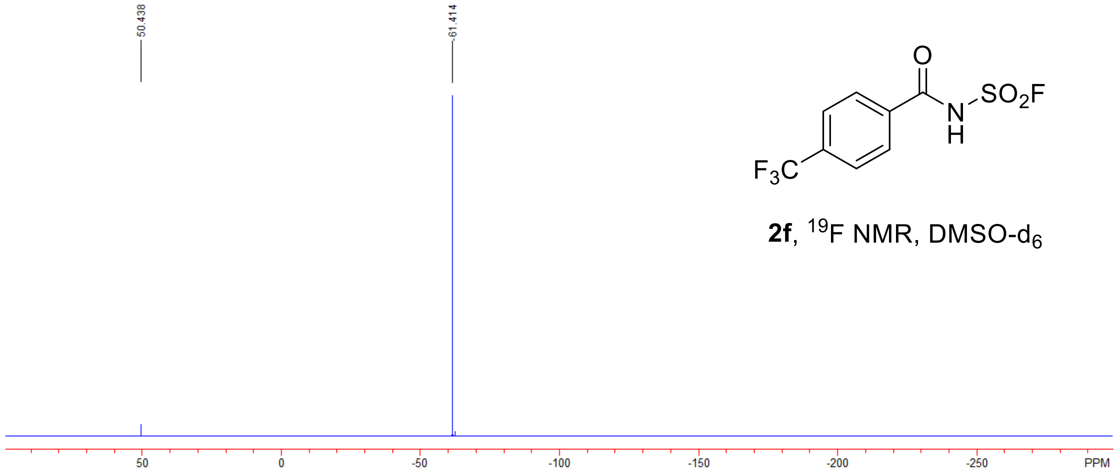
50.438
-61.414
2f, 19F NMR, DMSO-d6
50
0
-50
-100
-150
-200
-250
PPM

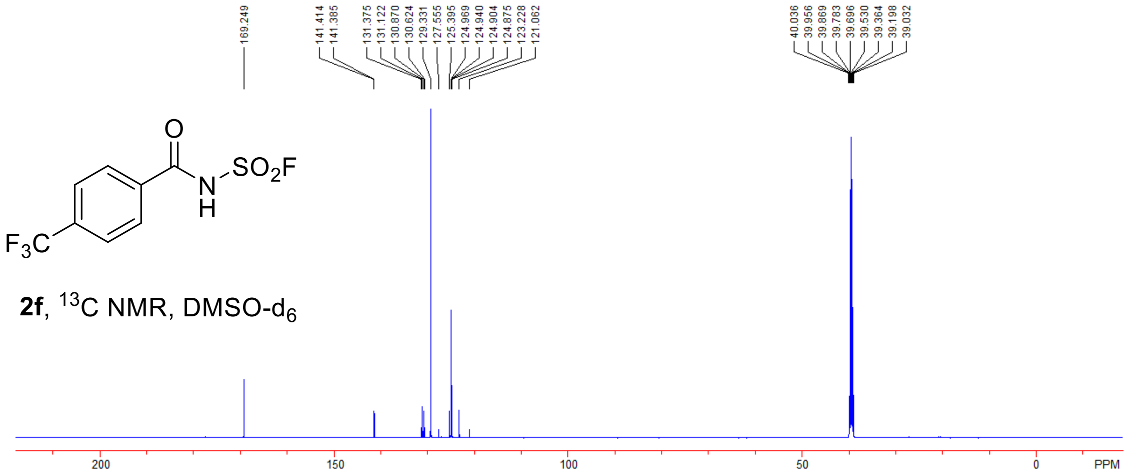
169.249
141.414
141.385
131.375
131.122
130.870
130.624
129.331
127.555
125.395
124.969
124.940
124.904
124.875
123.228
121.062
40.036
39.956
39.869
39.783
39.696
39.530
39.364
39.198
39.032
2f, 13C NMR, DMSO-d6
200
150
100
50
0
PPM

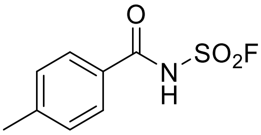

2g

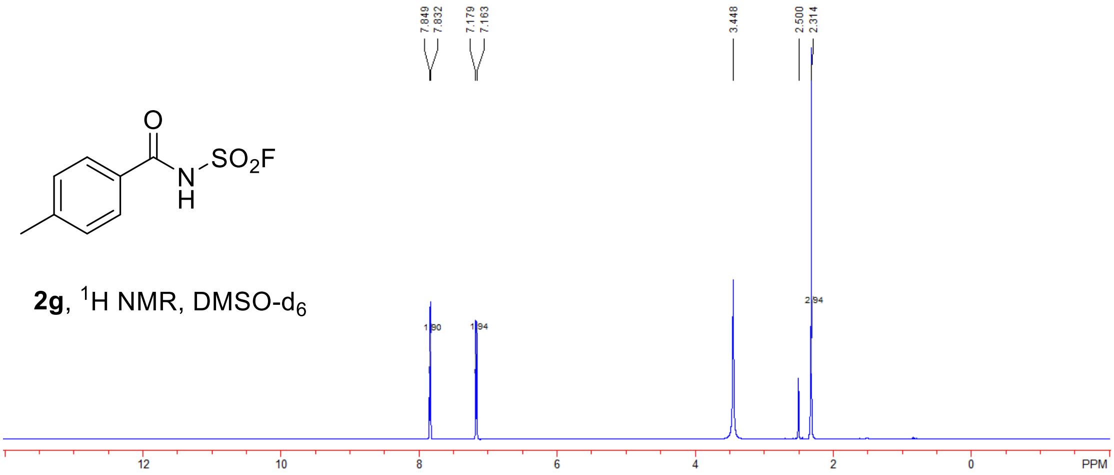
7.849
7.832
7.179
7.163
3.448
2.500
2.314
2g, ^{1}H NMR, DMSO-d$_6$
12
10
8
6
4
2
0
PPM

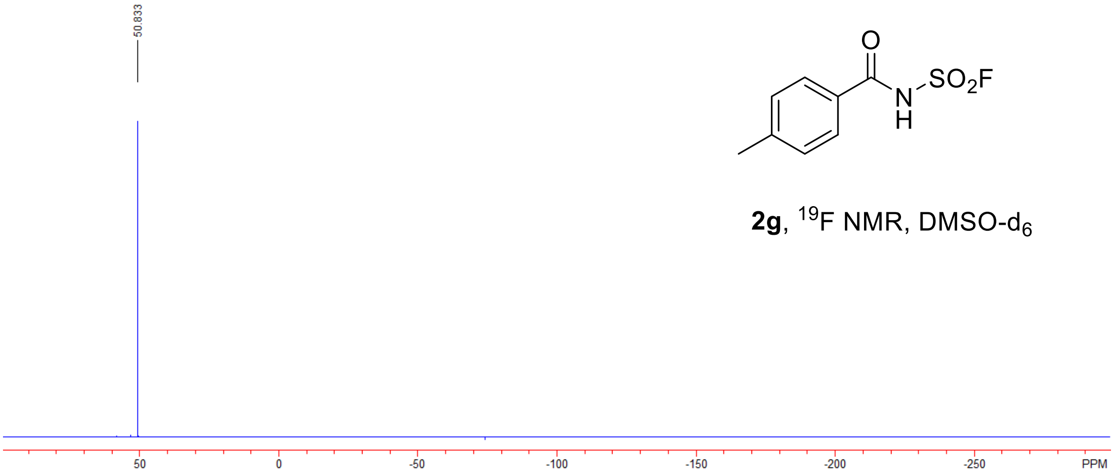
50.833
2g, ^{19}F NMR, DMSO-d$_6$
50
0
-50
-100
-150
-200
-250
PPM

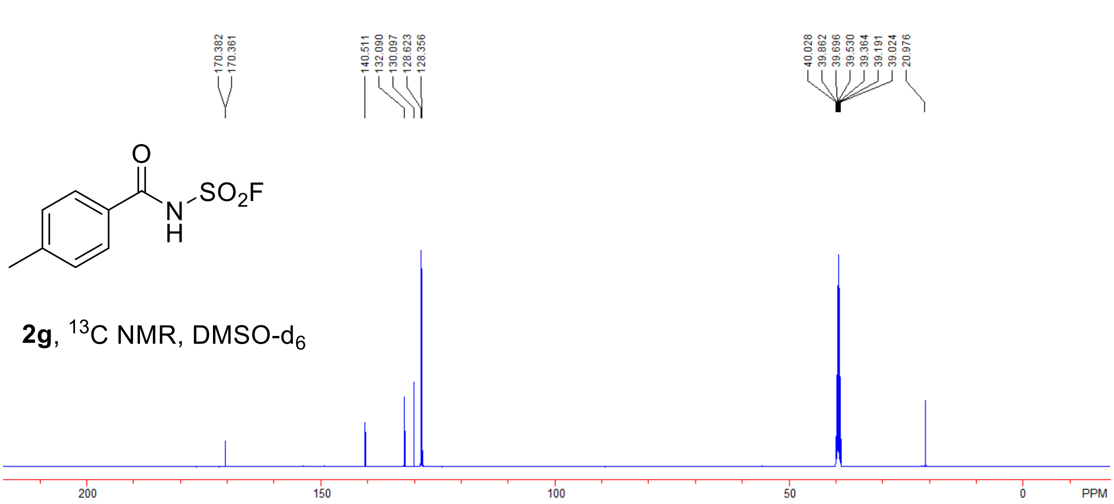
170.382
170.361
140.511
132.090
130.097
128.623
128.356
40.028
39.862
39.696
39.530
39.364
39.191
39.024
20.976
2g, ^{13}C NMR, DMSO-d$_6$
200
150
100
50
0
PPM

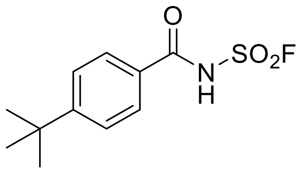

2h

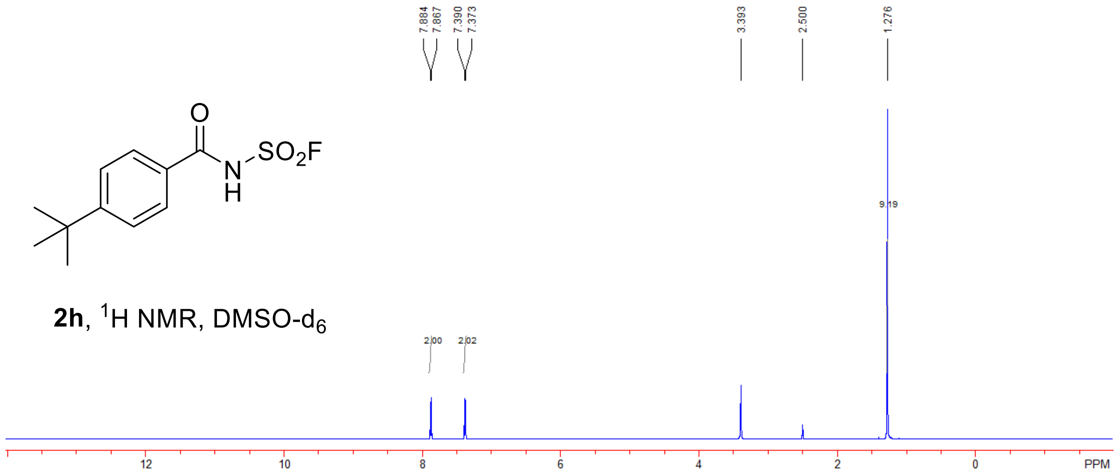
7.884
7.867
7.390
7.373
3.393
2.500
1.276
SO2F
N
H
O
9.19
2.00
2.02
2h, 1H NMR, DMSO-d6
12
10
8
6
4
2
0
PPM

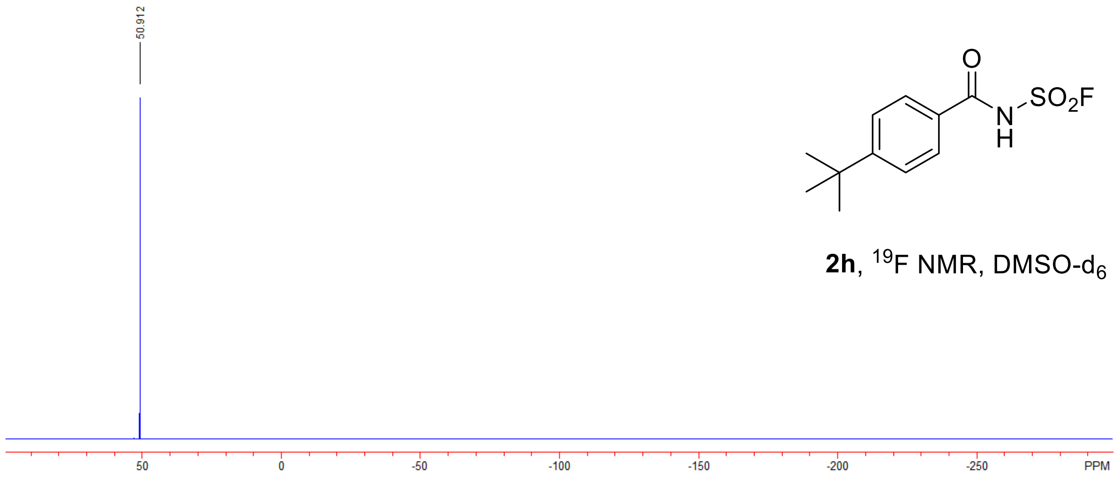
50.912
O
N
H
SO2F
2h, 19F NMR, DMSO-d6
50
0
-50
-100
-150
-200
-250
PPM

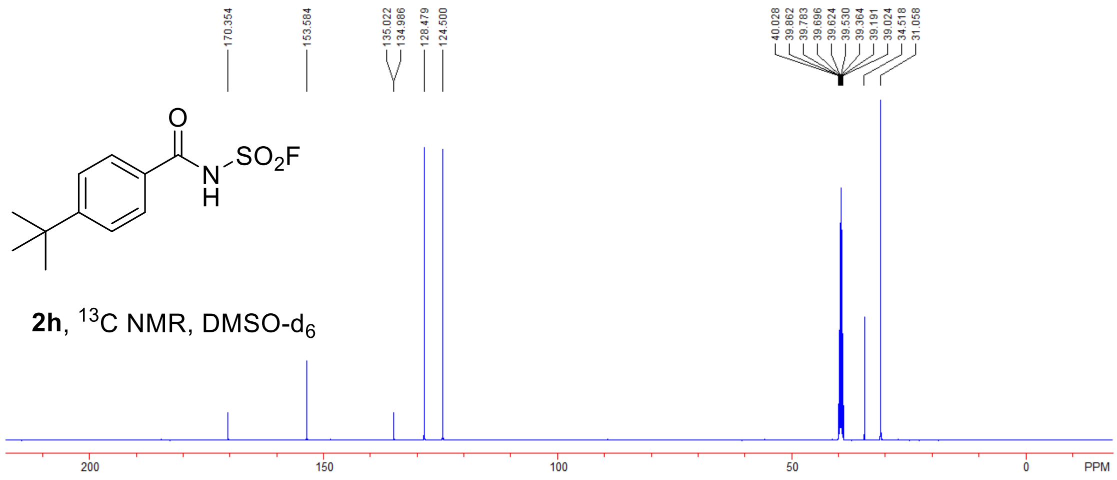
170.354
153.584
135.022
134.986
128.479
124.500
40.028
39.862
39.783
39.696
39.624
39.530
39.364
39.191
39.024
34.518
31.058
O
N
H
SO2F
2h, 13C NMR, DMSO-d6
200
150
100
50
0
PPM

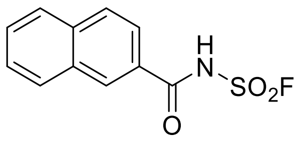

H
N
SO2F
O

2i

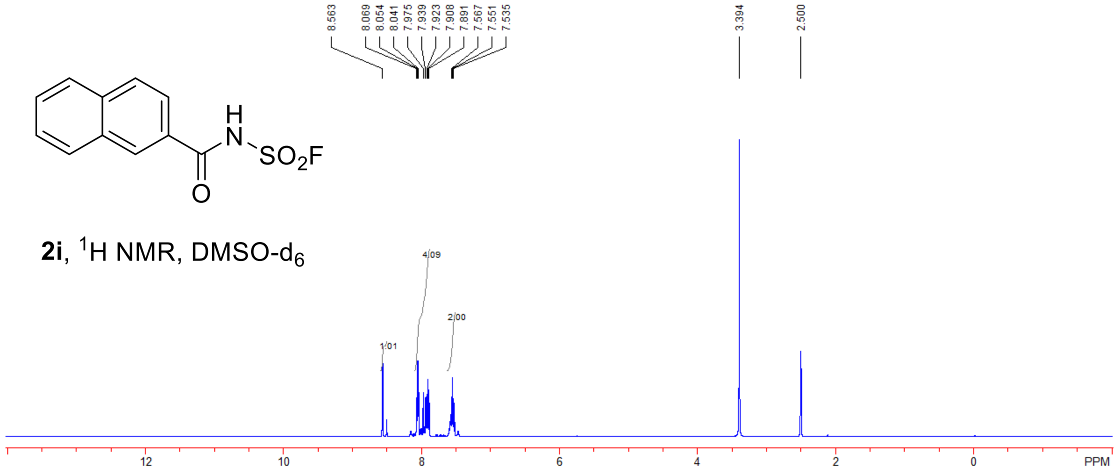

8.563
8.069
8.054
8.041
7.975
7.939
7.923
7.908
7.891
7.567
7.551
7.535
3.394
2.500
2i, 1H NMR, DMSO-d6
1.01
4.09
2.00
12
10
8
6
4
2
0
PPM

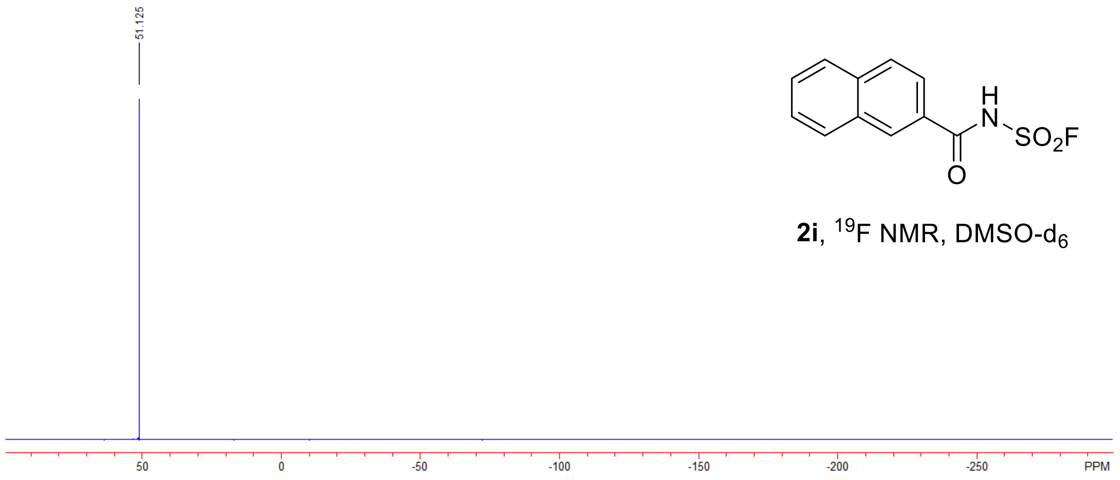

51.125
2i, 19F NMR, DMSO-d6
50
0
-50
-100
-150
-200
-250
PPM

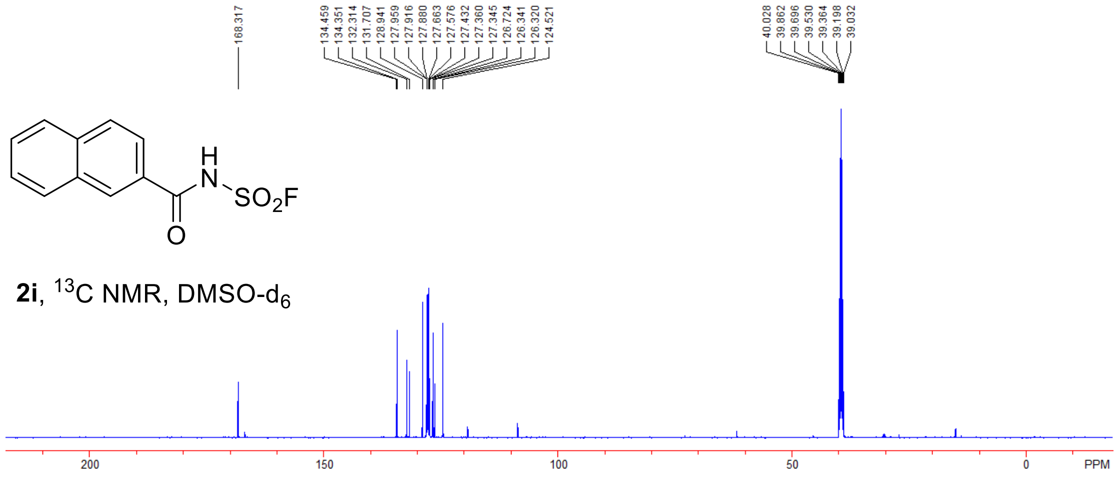

168.317
134.459
134.351
132.314
131.707
128.941
127.959
127.916
127.880
127.663
127.576
127.432
127.360
127.345
126.724
126.341
126.320
124.521
40.028
39.862
39.696
39.530
39.364
39.198
39.032
2i, 13C NMR, DMSO-d6
200
150
100
50
0
PPM

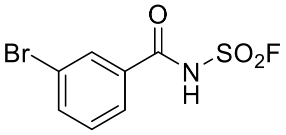
Br
O
N
H
SO2F

2j

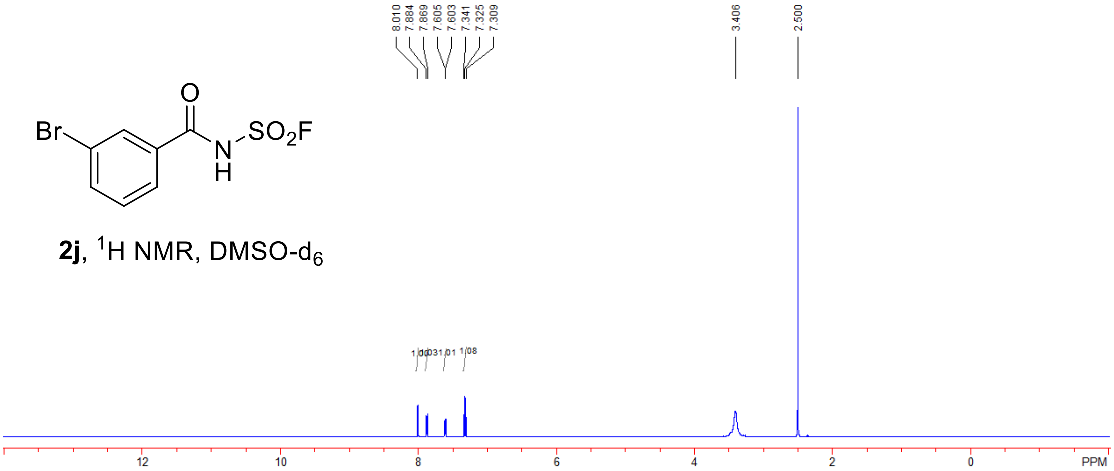
8.010
7.884
7.869
7.605
7.603
7.341
7.325
7.309
3.406
2.500
2j, 1H NMR, DMSO-d6
12
10
8
6
4
2
0
PPM

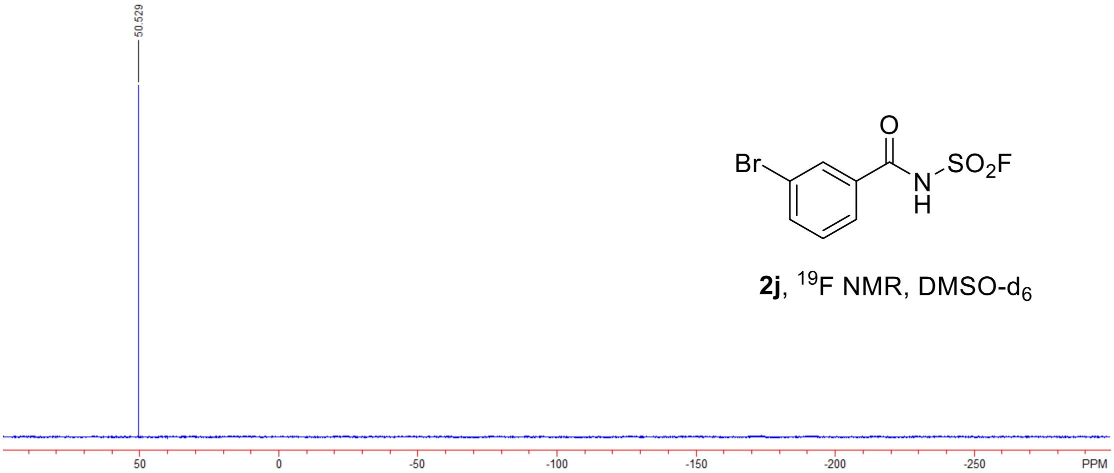
50.529
2j, 19F NMR, DMSO-d6
50
0
-50
-100
-150
-200
-250
PPM

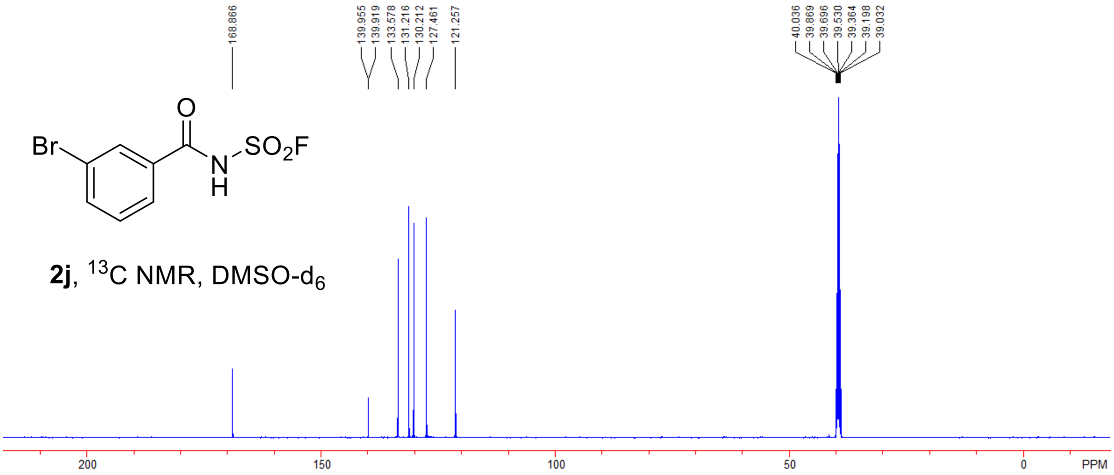
168.866
139.955
139.919
133.578
131.216
130.212
127.461
121.257
40.036
39.869
39.696
39.530
39.364
39.198
39.032
2j, 13C NMR, DMSO-d6
200
150
100
50
0
PPM

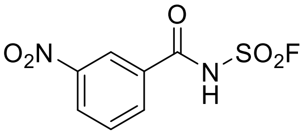

O
O2N
N
SO2F
H

2k

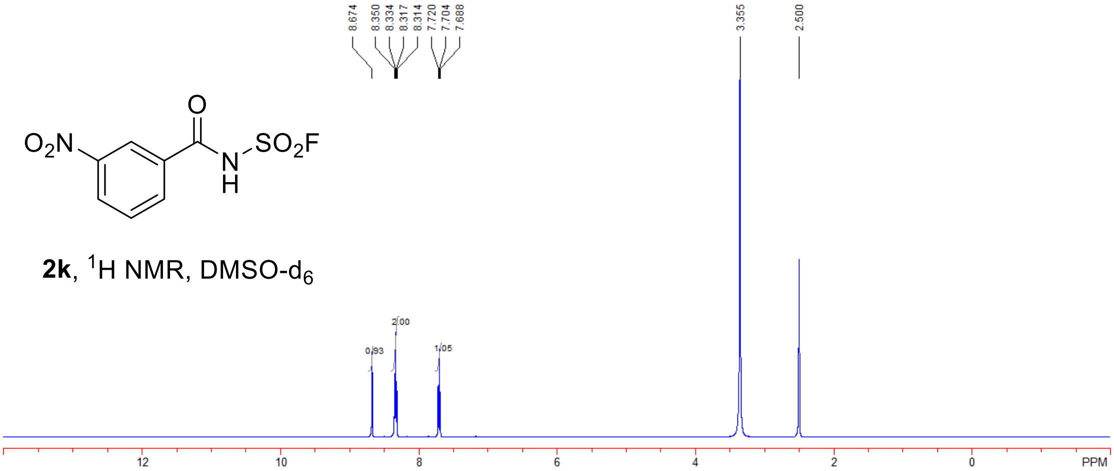

8.674
8.350
8.334
8.317
8.314
7.720
7.704
7.688
3.355
2.500
2k, 1H NMR, DMSO-d6
0.93
2.00
1.05
12
10
8
6
4
2
0
PPM

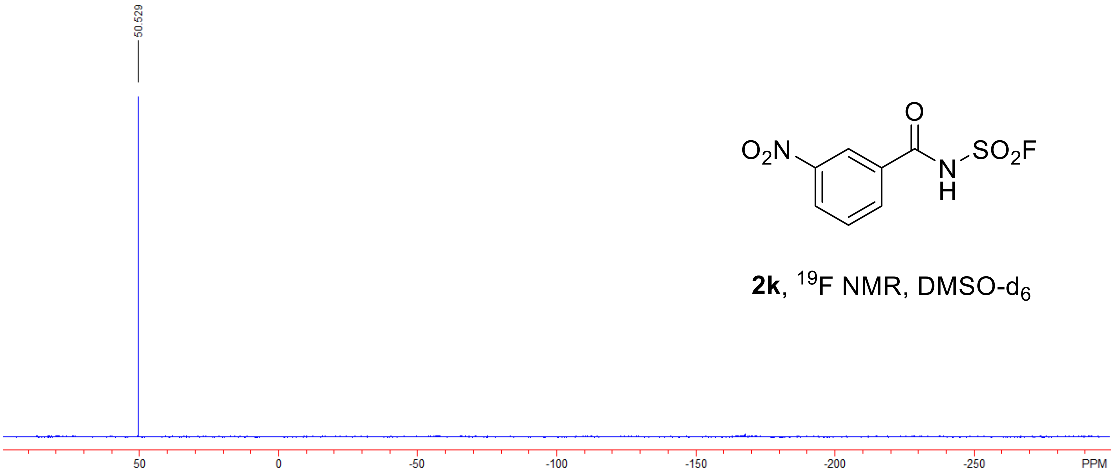

50.529
2k, 19F NMR, DMSO-d6
50
0
-50
-100
-150
-200
-250
PPM

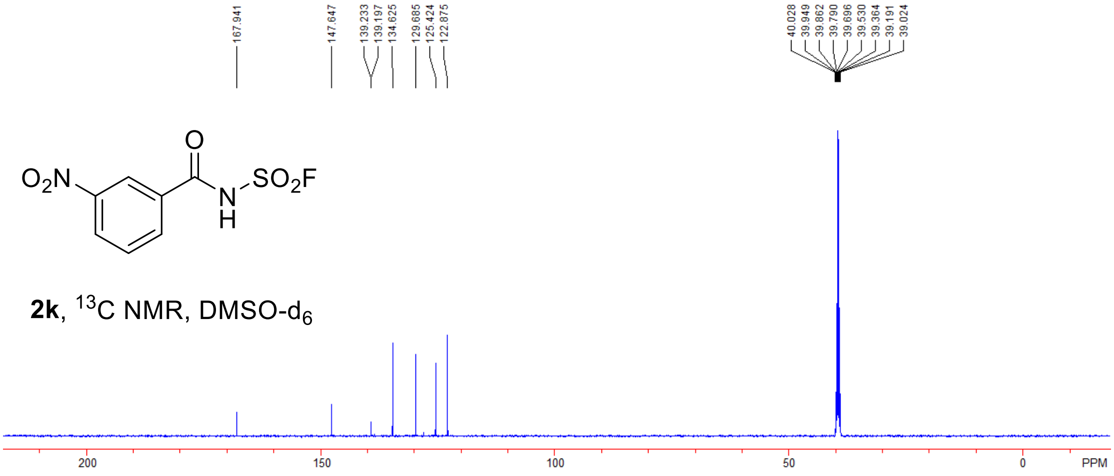

167.941
147.647
139.233
139.197
134.625
129.685
125.424
122.875
40.028
39.949
39.862
39.790
39.696
39.530
39.364
39.191
39.024
2k, 13C NMR, DMSO-d6
200
150
100
50
0
PPM

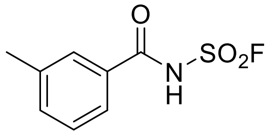

2l

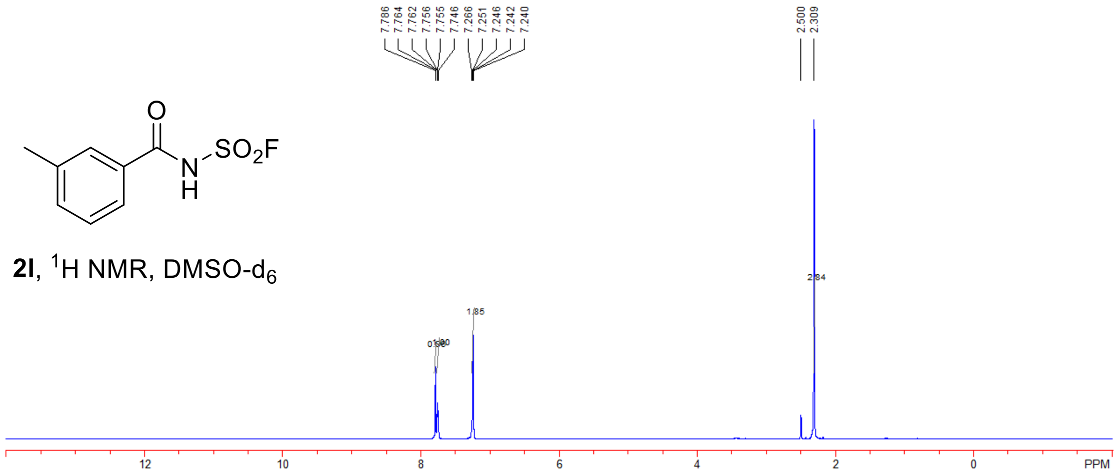

7.786
7.764
7.762
7.756
7.755
7.746
7.266
7.251
7.246
7.242
7.240
2.500
2.309
1.85
2.84
2l, ¹H NMR, DMSO-d6
12
10
8
6
4
2
0
PPM

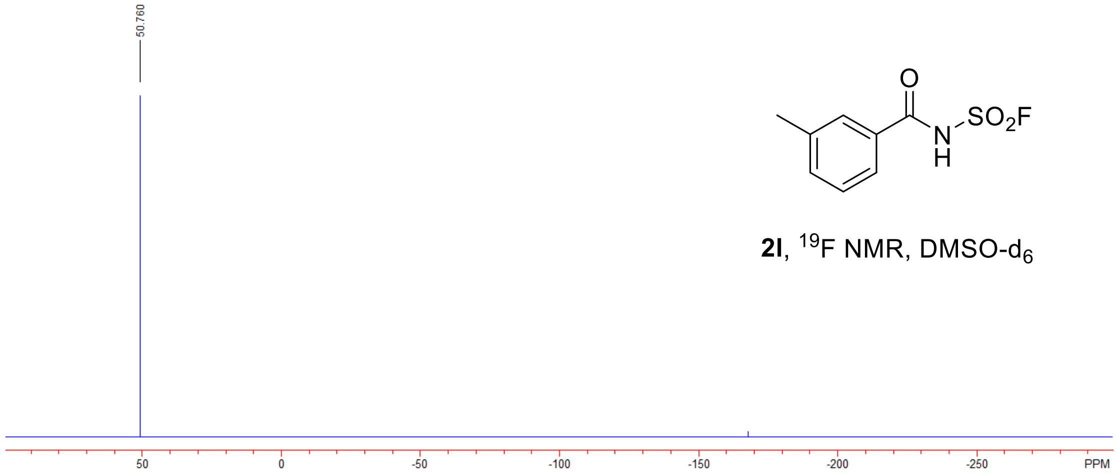

50.760
2l, ¹⁹F NMR, DMSO-d6
50
0
-50
-100
-150
-200
-250
PPM

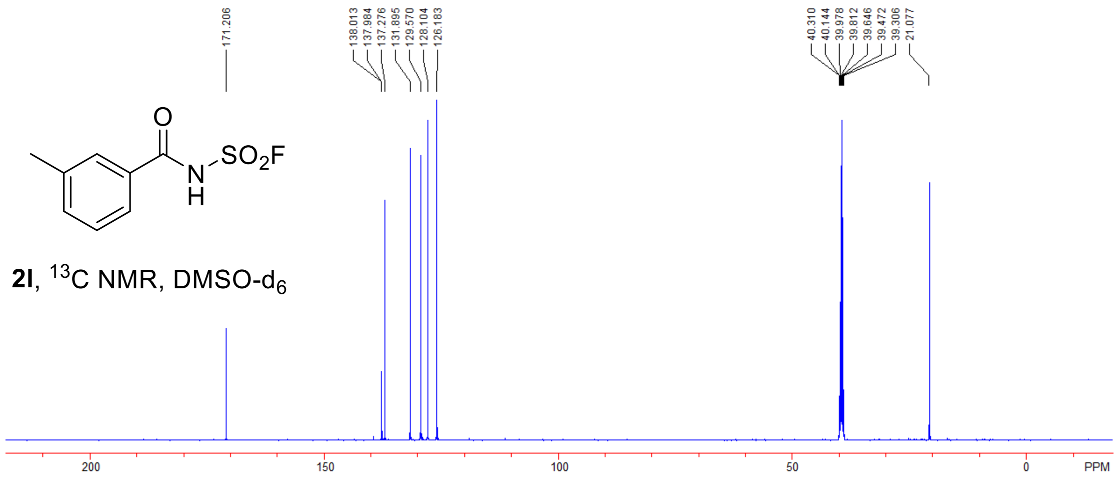

171.206
138.013
137.984
137.276
131.895
129.570
128.104
126.183
40.310
40.144
39.978
39.812
39.646
39.472
39.306
21.077
2l, ¹³C NMR, DMSO-d6
200
150
100
50
0
PPM

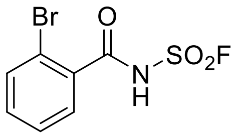
Br
O
SO2F
N
H

2m

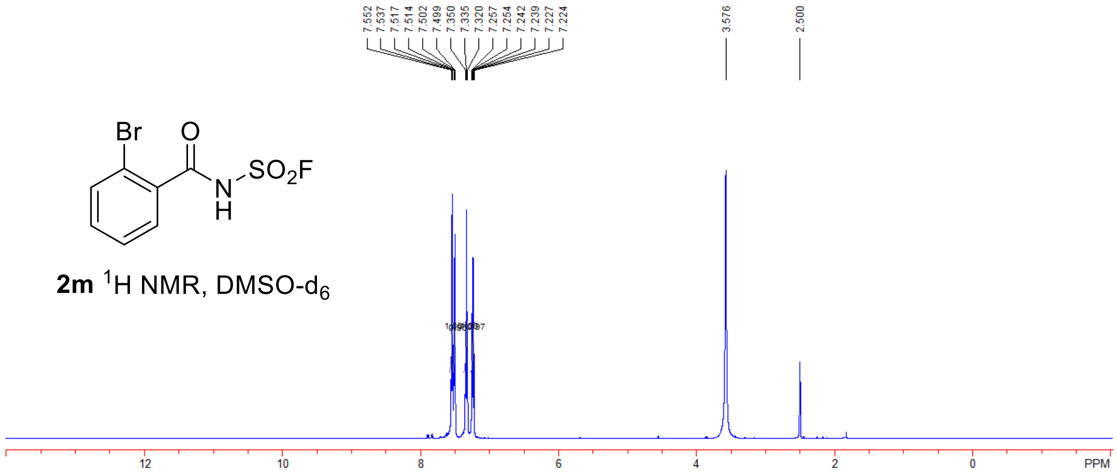
7.552
7.537
7.517
7.514
7.502
7.499
7.350
7.335
7.320
7.257
7.254
7.242
7.239
7.227
7.224
3.576
2.500
2m 1H NMR, DMSO-d6
12
10
8
6
4
2
0
PPM

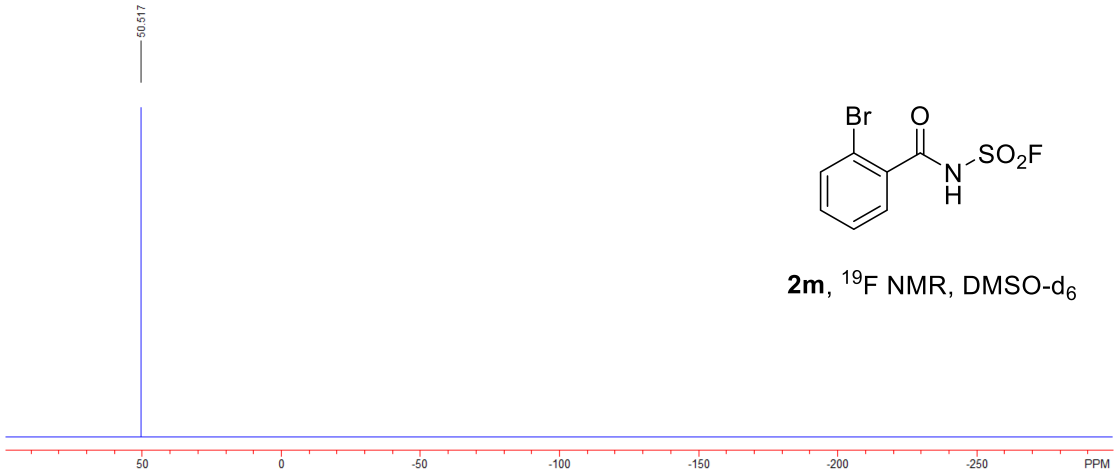
50.517
2m, 19F NMR, DMSO-d6
50
0
-50
-100
-150
-200
-250
PPM

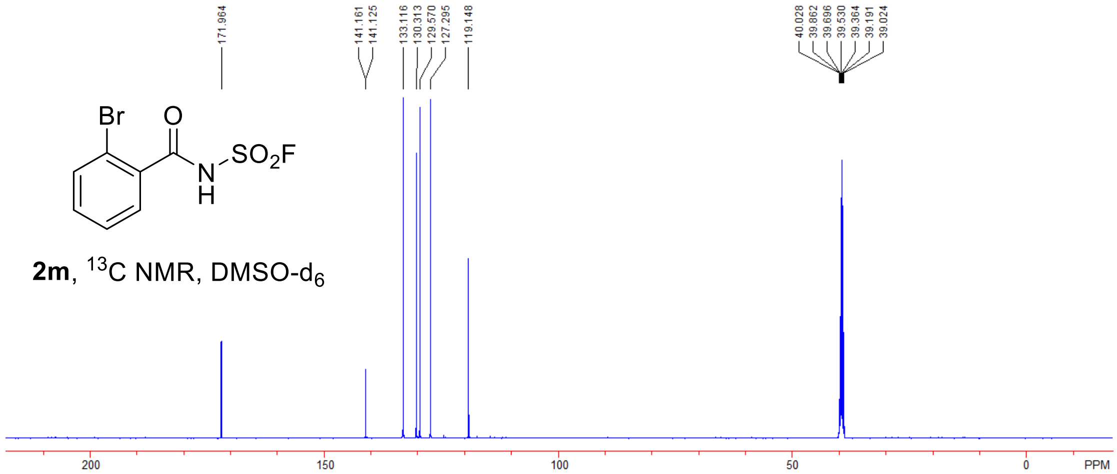
171.964
141.161
141.125
133.116
130.313
129.570
127.295
119.148
40.028
39.862
39.696
39.530
39.364
39.191
39.024
2m, 13C NMR, DMSO-d6
200
150
100
50
0
PPM

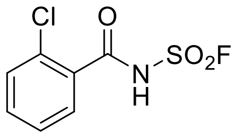

2n

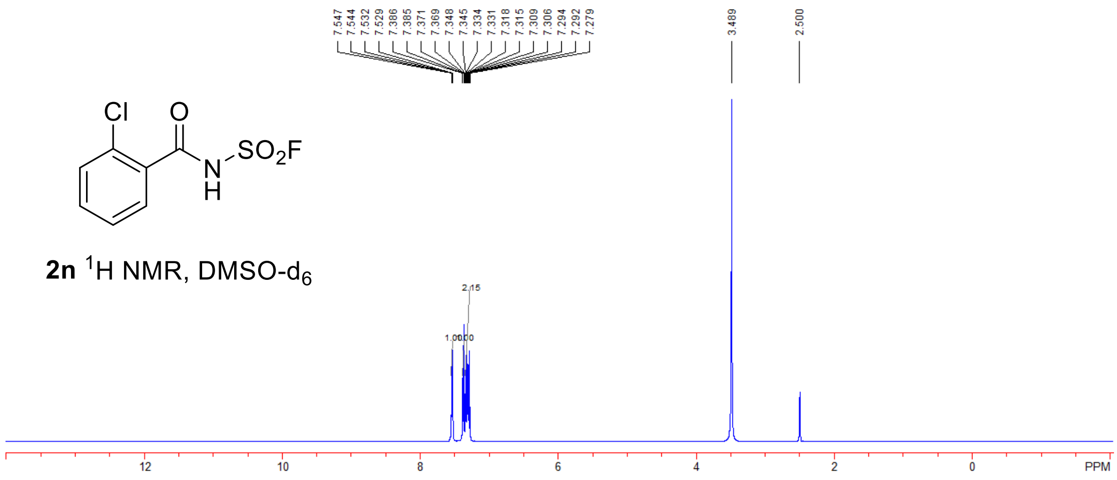
7.547
7.544
7.532
7.529
7.386
7.385
7.371
7.369
7.348
7.345
7.334
7.331
7.318
7.315
7.309
7.306
7.294
7.292
7.279
3.489
2.500
2n 1H NMR, DMSO-d6
2.15
12
10
8
6
4
2
0
PPM

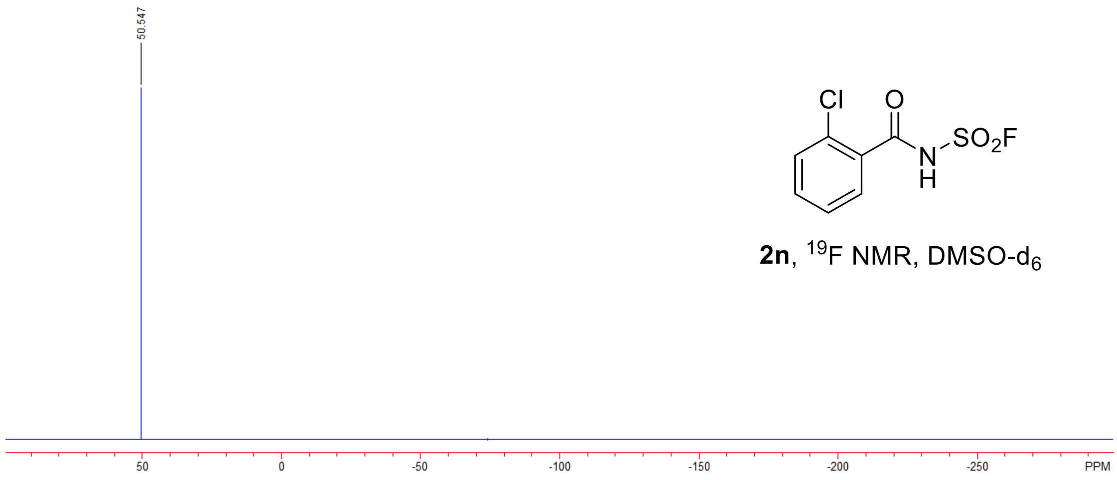
50.547
2n, 19F NMR, DMSO-d6
50
0
-50
-100
-150
-200
-250
PPM

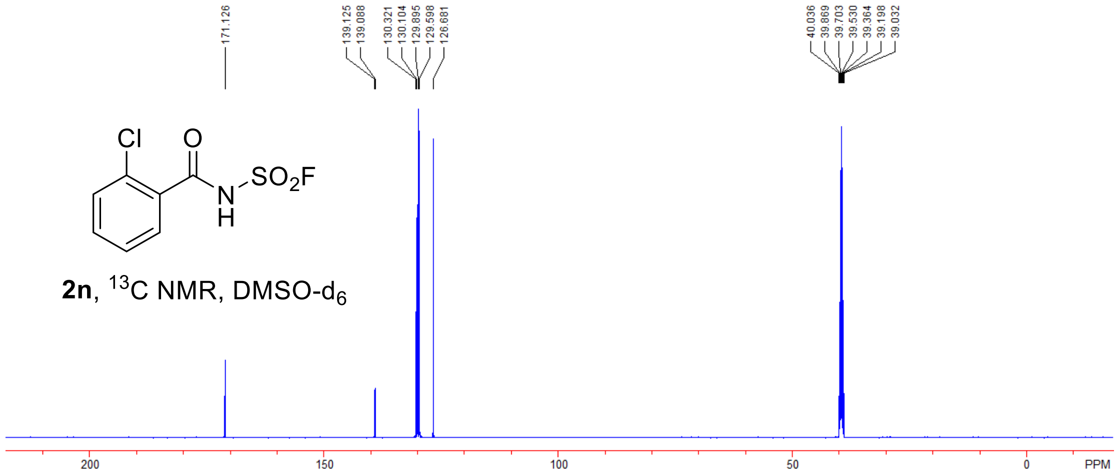
171.126
139.125
139.088
130.321
130.104
129.895
129.598
126.681
40.036
39.869
39.703
39.530
39.364
39.198
39.032
2n, 13C NMR, DMSO-d6
200
150
100
50
0
PPM

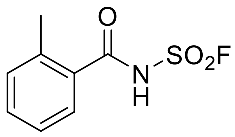

2o

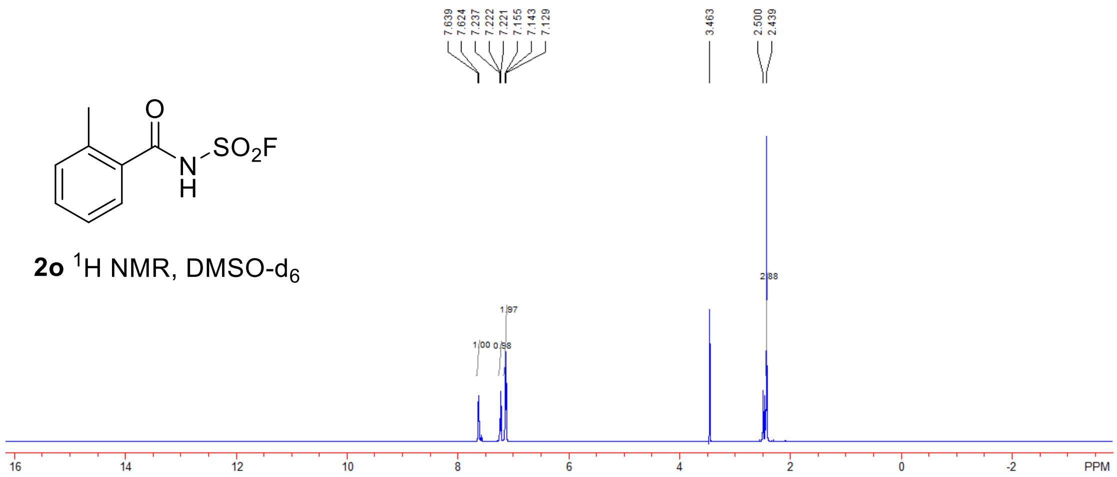
7.639
7.624
7.237
7.222
7.221
7.155
7.143
7.129
3.463
2.500
2.439
1.00
0.98
1.97
2.88
2o 1H NMR, DMSO-d6
16
14
12
10
8
6
4
2
0
-2
PPM

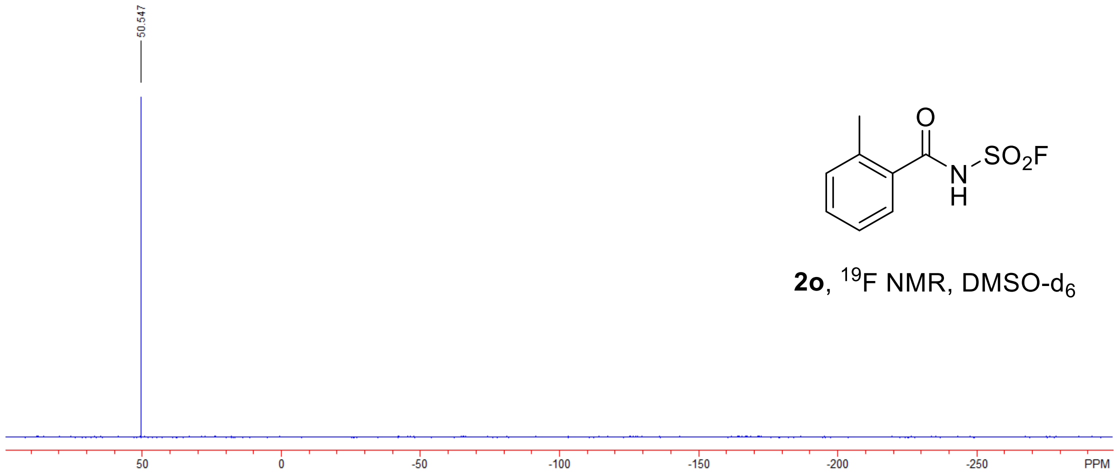
50.547
2o, 19F NMR, DMSO-d6
50
0
-50
-100
-150
-200
-250
PPM

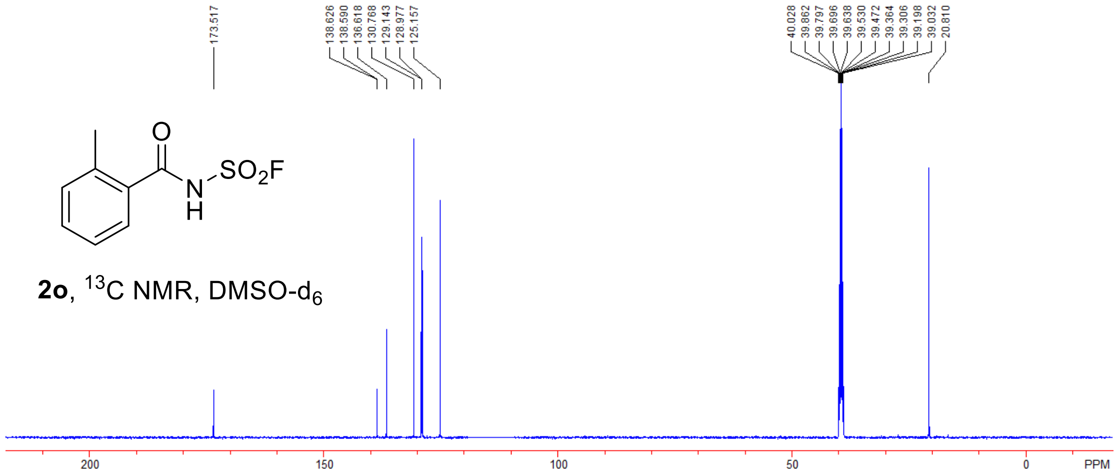
173.517
138.626
138.590
136.618
130.768
129.143
128.977
125.157
40.028
39.862
39.797
39.696
39.638
39.530
39.472
39.364
39.306
39.198
39.032
20.810
2o, 13C NMR, DMSO-d6
200
150
100
50
0
PPM

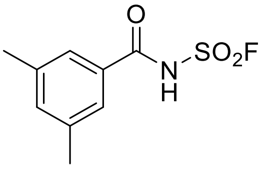

2p

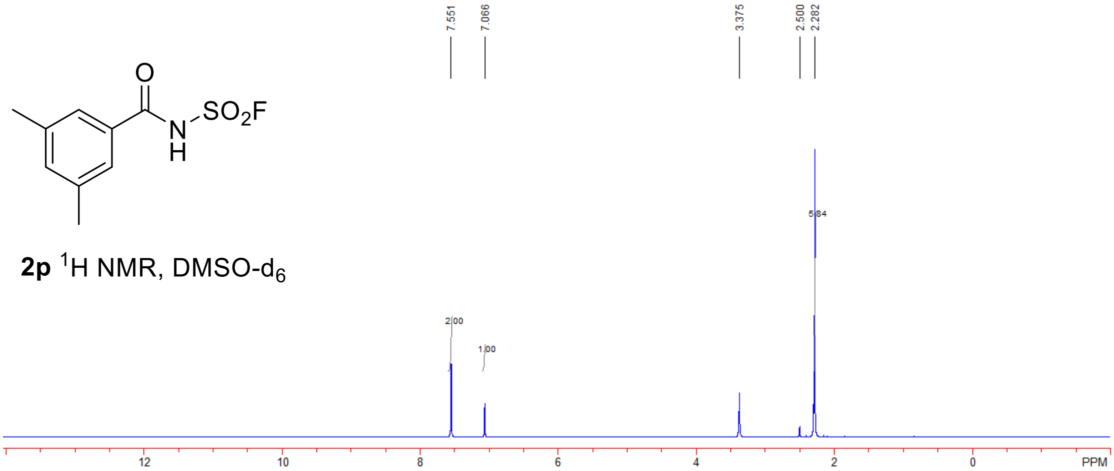
7.551
7.066
3.375
2.500
2.282
2p ¹H NMR, DMSO-d6
2.00
1.00
5.84
12
10
8
6
4
2
0
PPM

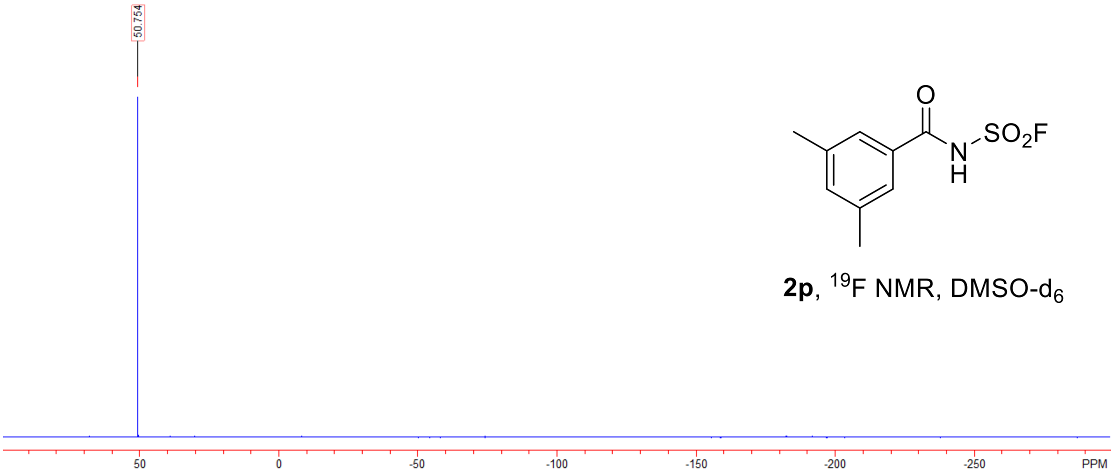
50.754
2p, ¹⁹F NMR, DMSO-d6
50
0
-50
-100
-150
-200
-250
PPM

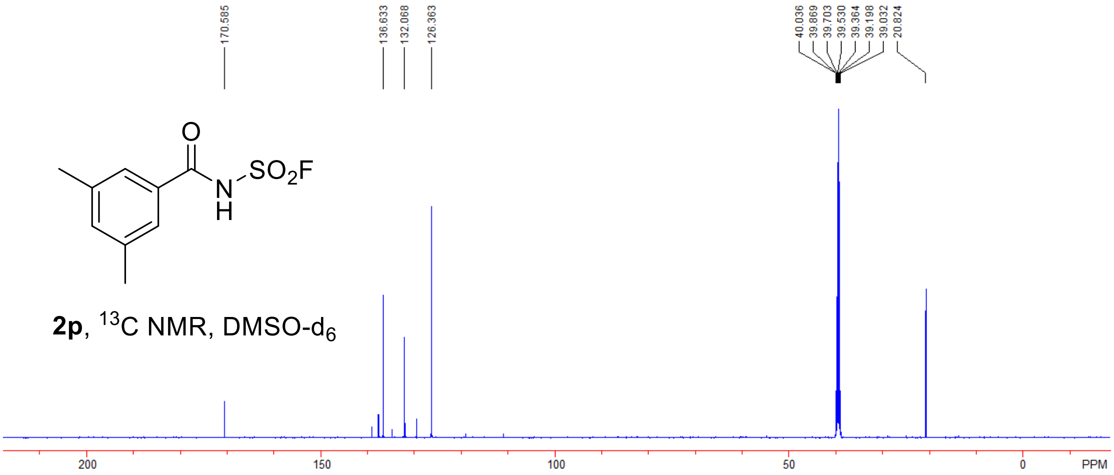
170.585
136.633
132.068
126.363
40.036
39.869
39.703
39.530
39.364
39.198
39.032
20.824
2p, ¹³C NMR, DMSO-d6
200
150
100
50
0
PPM

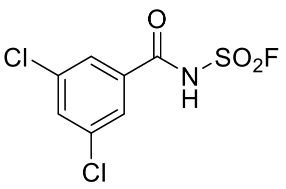

2q

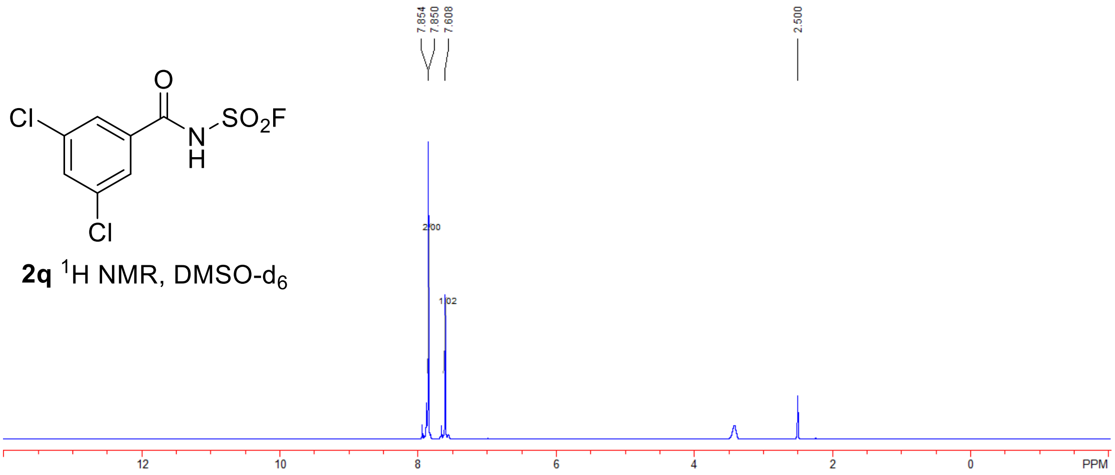

2q ^{1}H NMR, DMSO-d_6

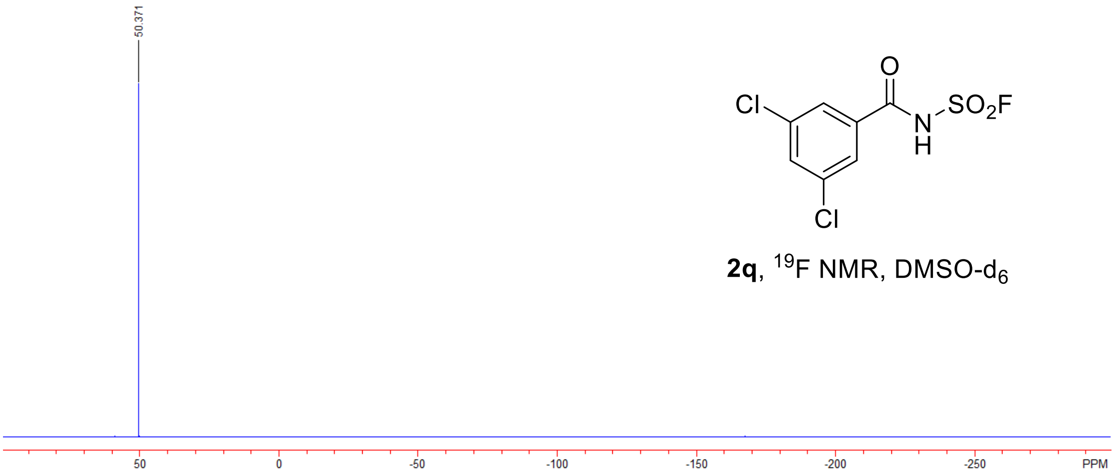

2q, ^{19}F NMR, DMSO-d_6

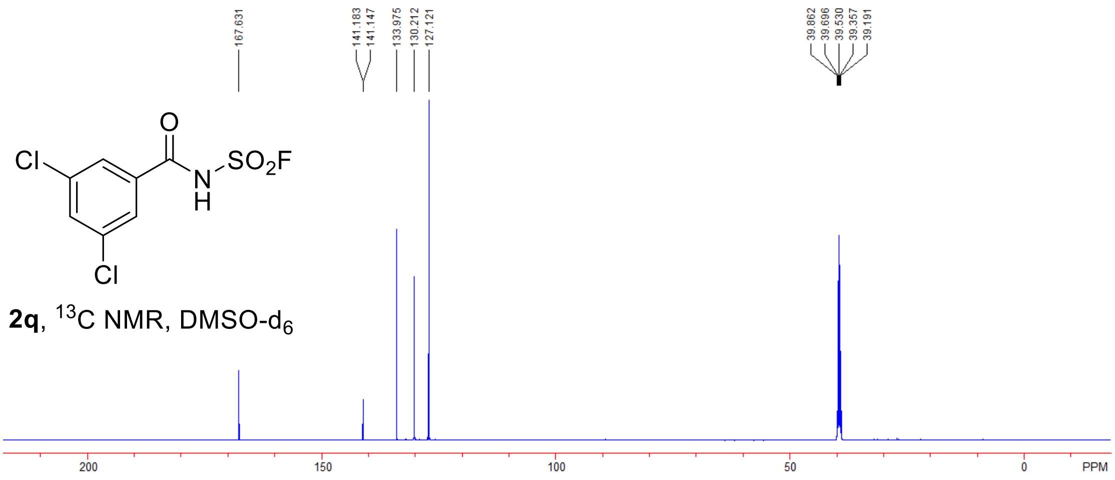

2q, ^{13}C NMR, DMSO-d_6

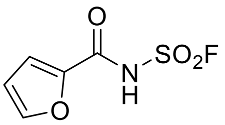
O
N
H
SO2F
O

2r

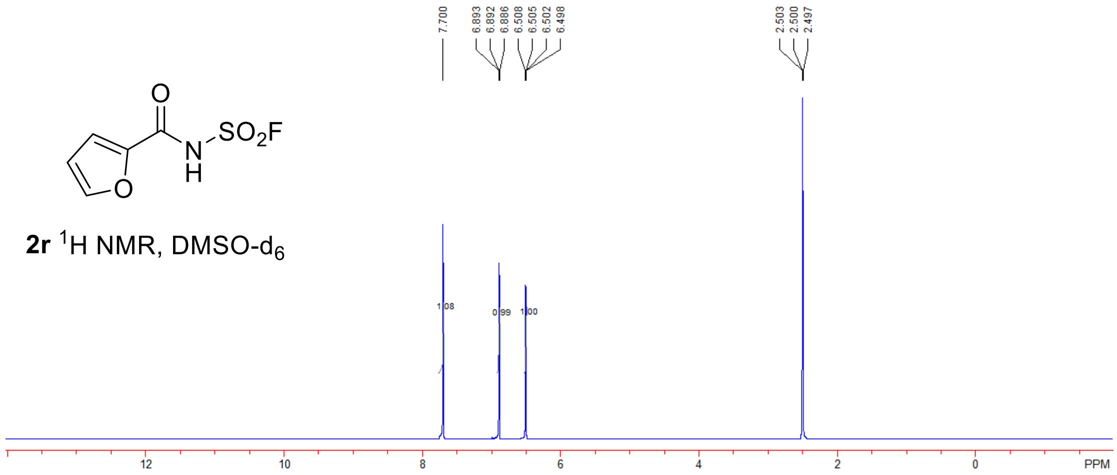
7.700
6.893
6.892
6.886
6.508
6.505
6.502
6.498
2.503
2.500
2.497
2r 1H NMR, DMSO-d6
1.08
0.99
1.00
12
10
8
6
4
2
0
PPM

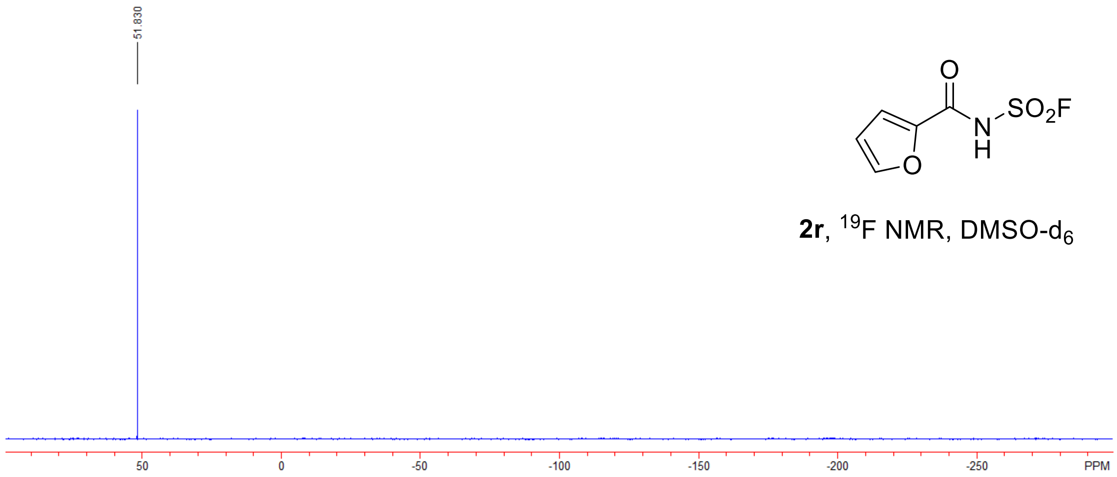
51.830
2r, 19F NMR, DMSO-d6
50
0
-50
-100
-150
-200
-250
PPM

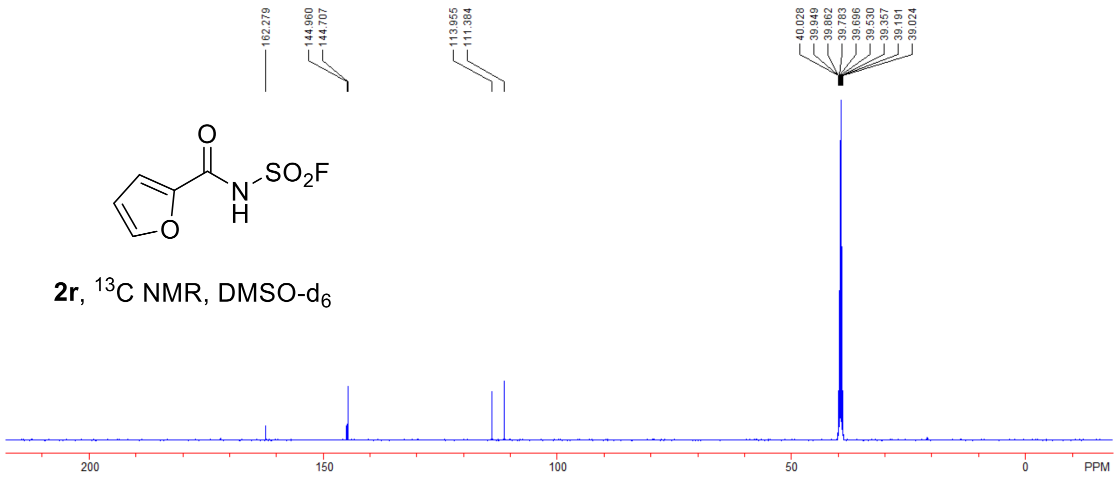
162.279
144.960
144.707
113.955
111.384
40.028
39.949
39.862
39.783
39.696
39.530
39.357
39.191
39.024
2r, 13C NMR, DMSO-d6
200
150
100
50
0
PPM

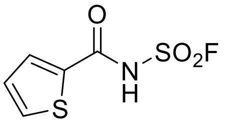

2s

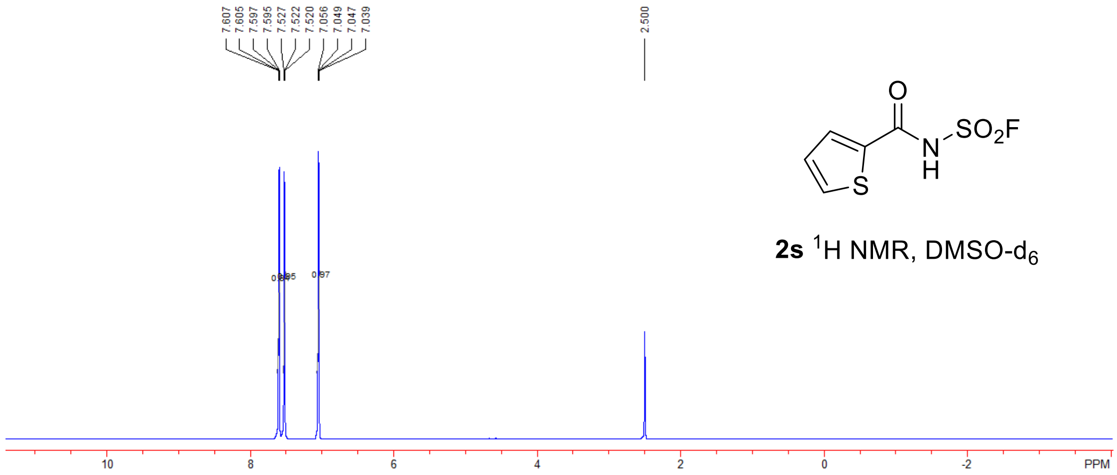
7.607
7.605
7.597
7.595
7.527
7.522
7.520
7.056
7.049
7.047
7.039
2.500
2s ^{1}H NMR, DMSO-d$_6$
10
8
6
4
2
0
-2
PPM

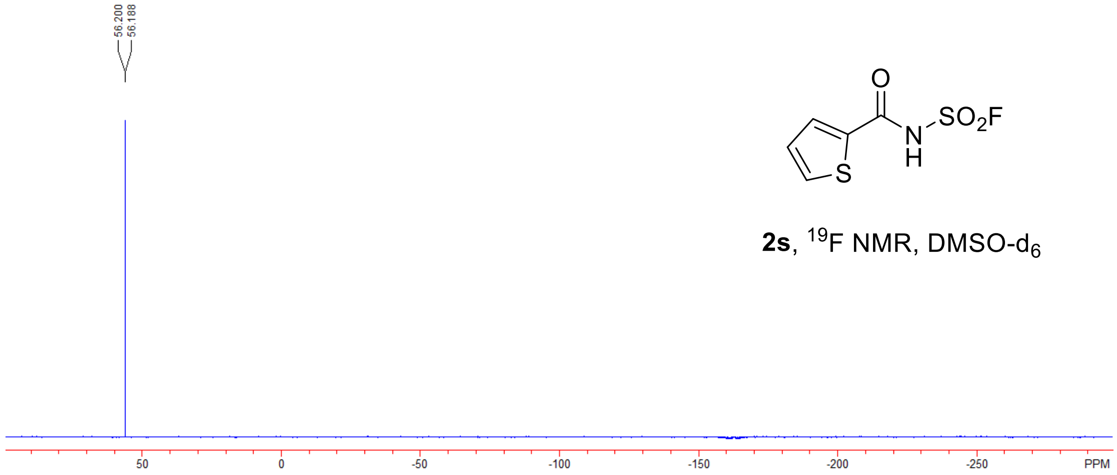
56.200
56.188
2s, ^{19}F NMR, DMSO-d$_6$
50
0
-50
-100
-150
-200
-250
PPM

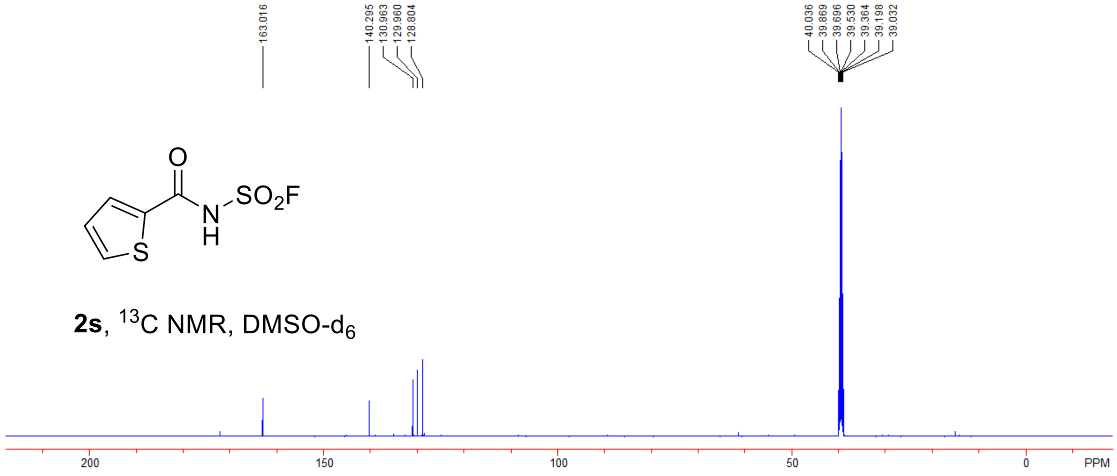
163.016
140.295
130.963
129.960
128.804
40.036
39.869
39.696
39.530
39.364
39.198
39.032
2s, ^{13}C NMR, DMSO-d$_6$
200
150
100
50
0
PPM

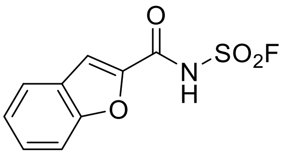

2t

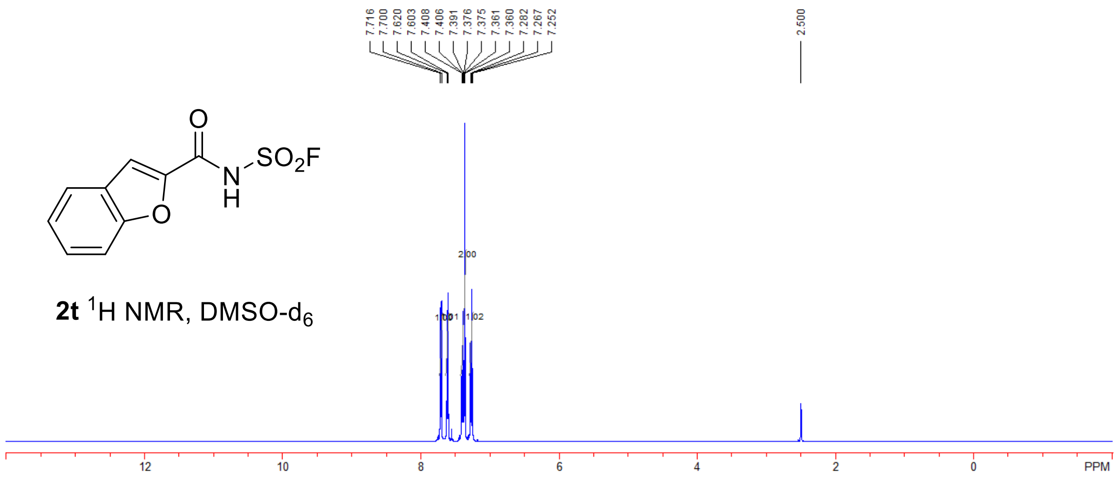
7.716
7.700
7.620
7.603
7.408
7.406
7.391
7.376
7.375
7.361
7.360
7.282
7.267
7.252
2.500
2.00
1.02
2t 1H NMR, DMSO-d6
12
10
8
6
4
2
0
PPM

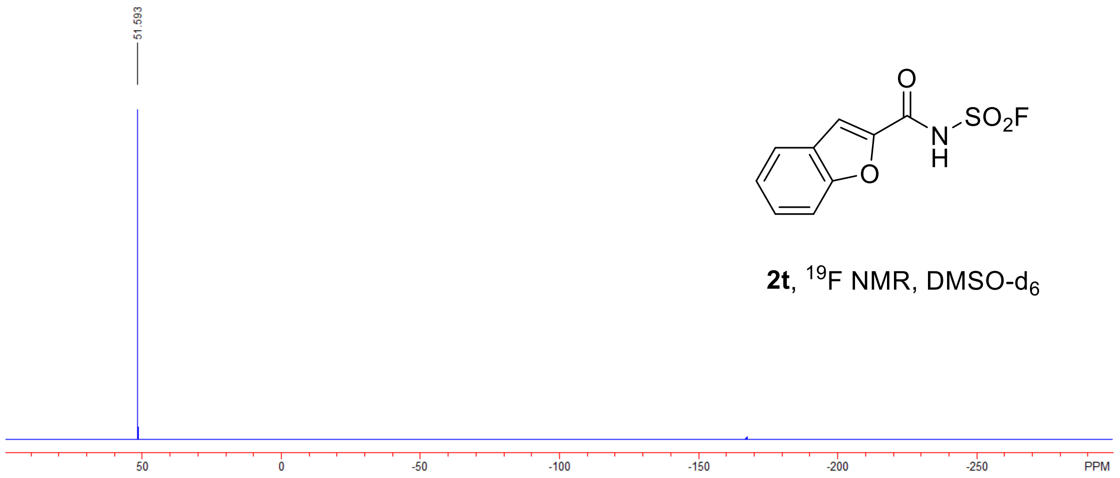
51.593
2t, 19F NMR, DMSO-d6
50
0
-50
-100
-150
-200
-250
PPM

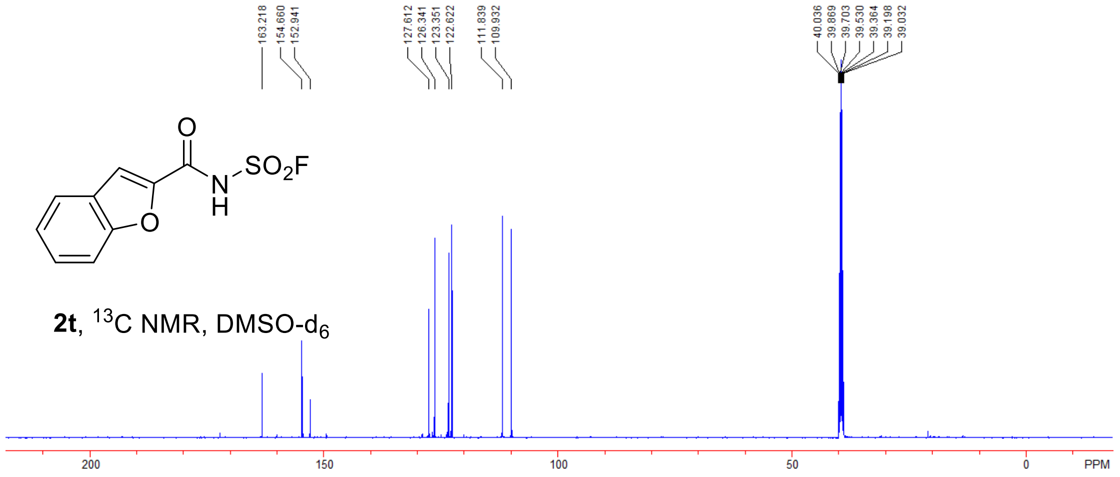
163.218
154.660
152.941
127.612
126.341
123.351
122.622
111.839
109.932
40.036
39.869
39.703
39.530
39.364
39.198
39.032
2t, 13C NMR, DMSO-d6
200
150
100
50
0
PPM

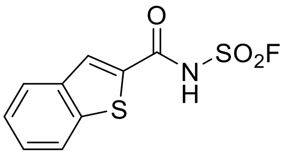
O
N
H
SO2F
S

2u

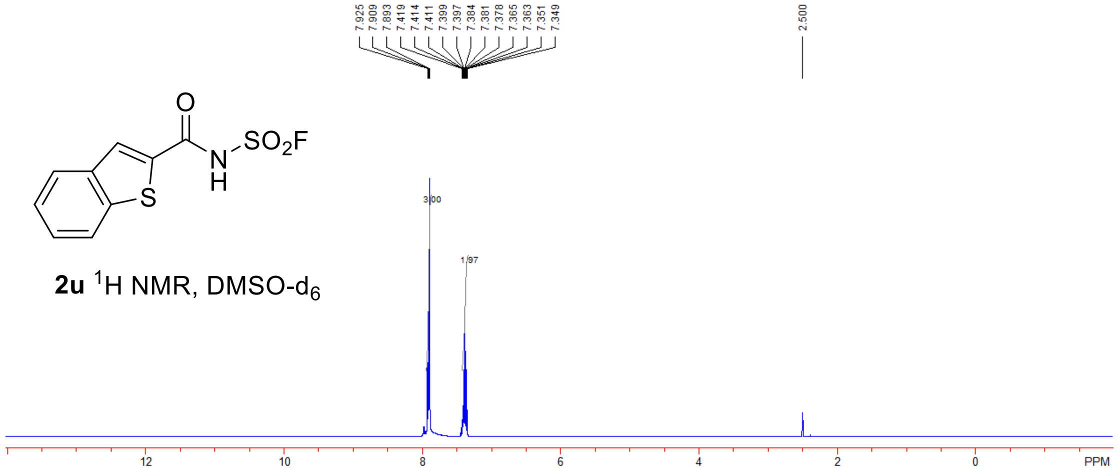
7.925
7.909
7.893
7.419
7.414
7.411
7.399
7.397
7.384
7.381
7.378
7.365
7.363
7.351
7.349
2.500
3.00
1.97
2u 1H NMR, DMSO-d6
12
10
8
6
4
2
0
PPM

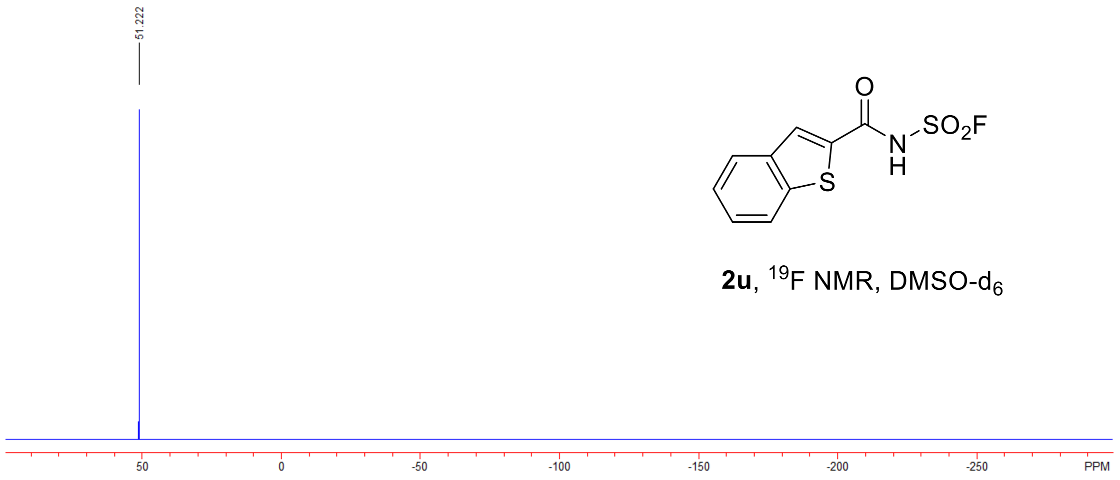
51.222
2u, 19F NMR, DMSO-d6
50
0
-50
-100
-150
-200
-250
PPM

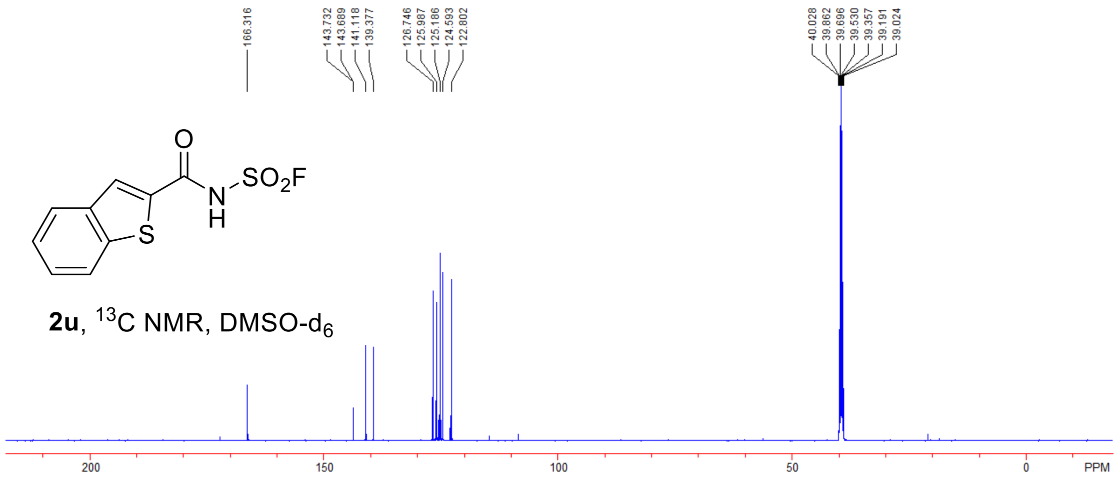
166.316
143.732
143.689
141.118
139.377
126.746
125.987
125.186
124.593
122.802
40.028
39.862
39.696
39.530
39.357
39.191
39.024
2u, 13C NMR, DMSO-d6
200
150
100
50
0
PPM

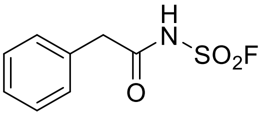

2v

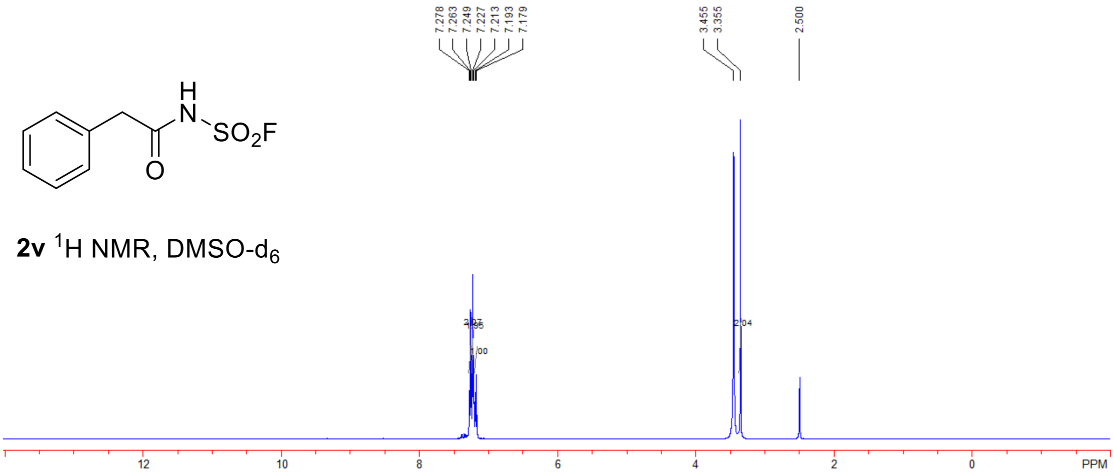
7.278
7.263
7.249
7.227
7.213
7.193
7.179
3.455
3.355
2.500
2v ^{1}H NMR, DMSO-d$_6$
12
10
8
6
4
2
0
PPM

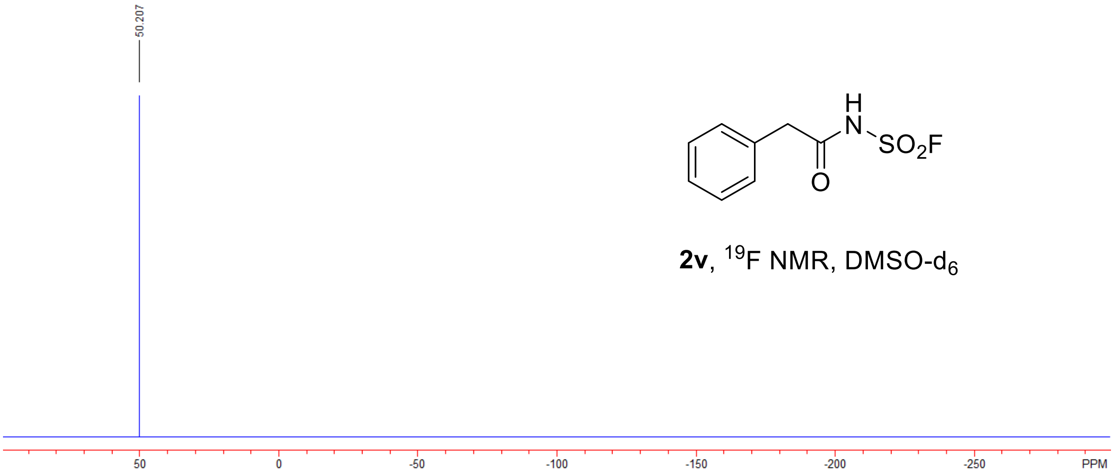
50.207
2v, ^{19}F NMR, DMSO-d$_6$
50
0
-50
-100
-150
-200
-250
PPM

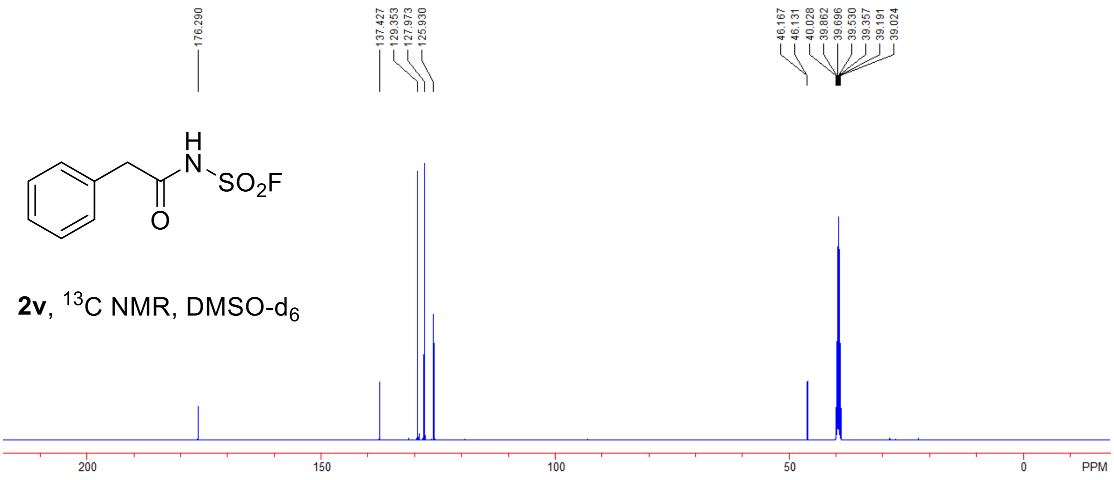
176.290
137.427
129.353
127.973
125.930
46.167
46.131
40.028
39.862
39.696
39.530
39.357
39.191
39.024
2v, ^{13}C NMR, DMSO-d$_6$
200
150
100
50
0
PPM

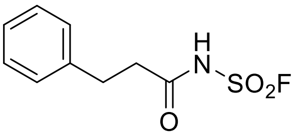
H
N
SO2F
O

2w

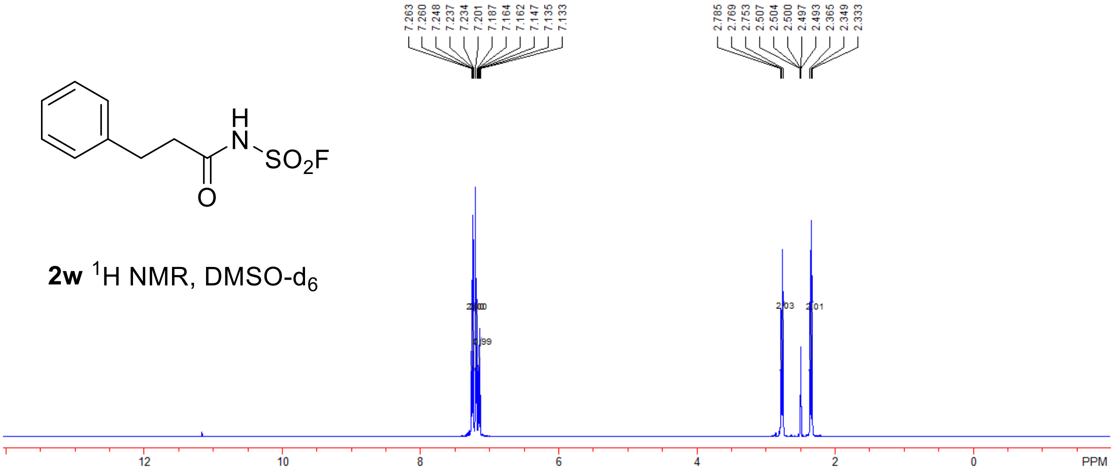
7.263 7.260 7.248 7.237 7.234 7.201 7.187 7.164 7.162 7.147 7.135 7.133
2.785 2.769 2.753 2.507 2.504 2.500 2.497 2.493 2.365 2.349 2.333
2w 1H NMR, DMSO-d6
2.03
2.01
12 10 8 6 4 2 0 PPM

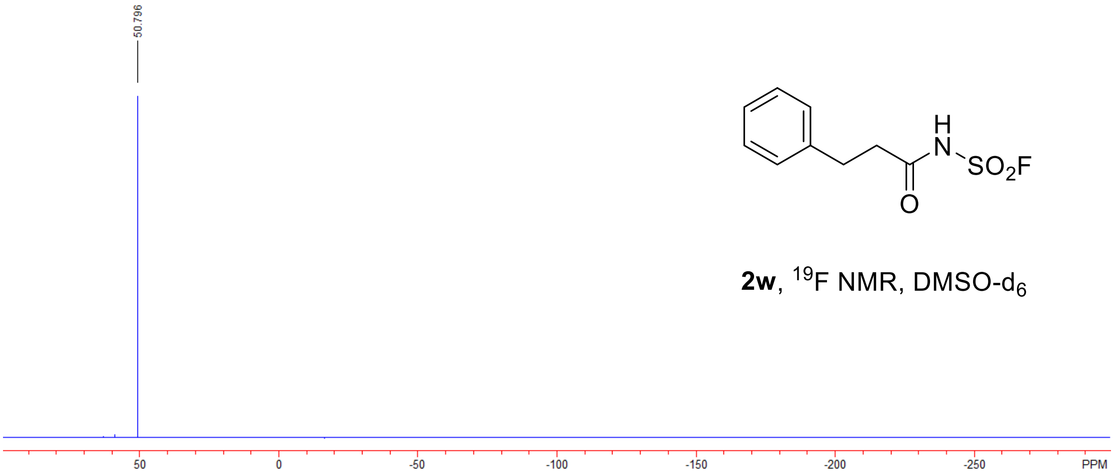
50.796
2w, 19F NMR, DMSO-d6
50 0 -50 -100 -150 -200 -250 PPM

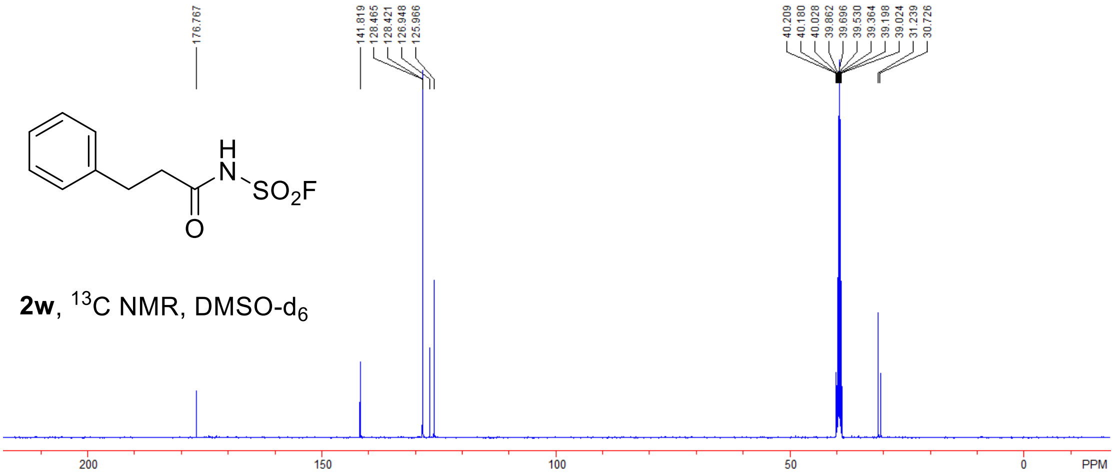
176.767
141.819 128.465 128.421 126.948 125.966
40.209 40.180 40.028 39.862 39.696 39.530 39.364 39.198 39.024 31.239 30.726
2w, 13C NMR, DMSO-d6
200 150 100 50 0 PPM

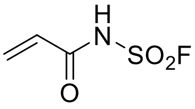

2x

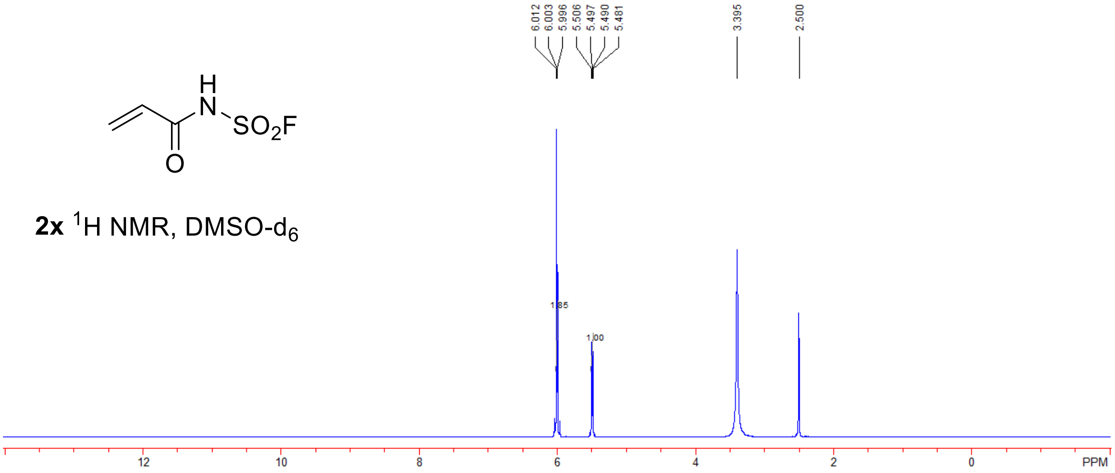
6.012
6.003
5.996
5.506
5.497
5.490
5.481
3.395
2.500
2x ^{1}H NMR, DMSO-d$_6$
1.85
1.00
12
10
8
6
4
2
0
PPM

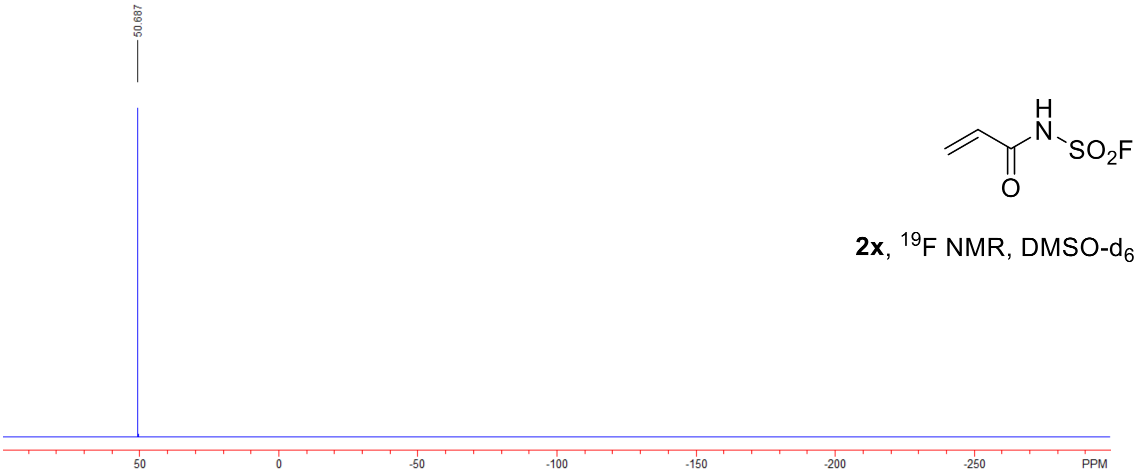
50.687
2x, ^{19}F NMR, DMSO-d$_6$
50
0
-50
-100
-150
-200
-250
PPM

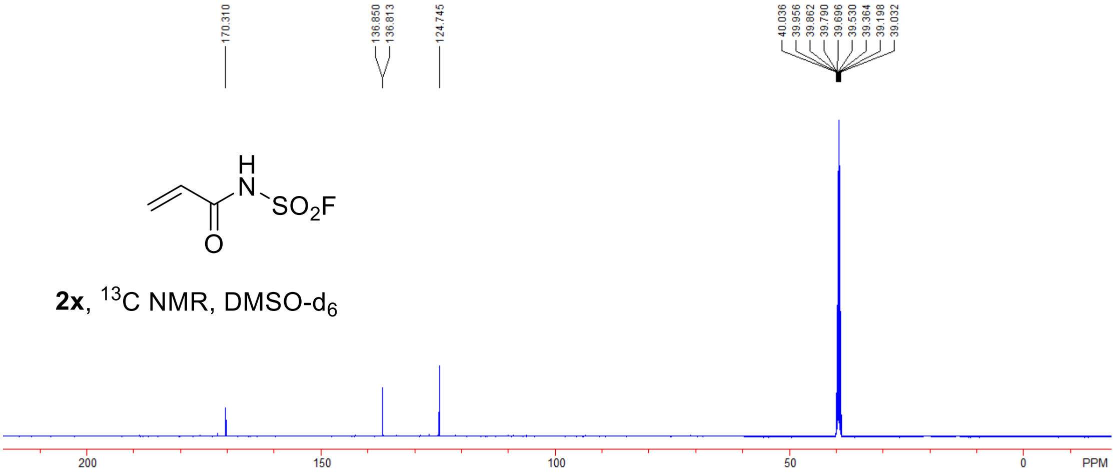
170.310
136.850
136.813
124.745
40.036
39.956
39.862
39.790
39.696
39.530
39.364
39.198
39.032
2x, ^{13}C NMR, DMSO-d$_6$
200
150
100
50
0
PPM

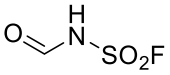

2y

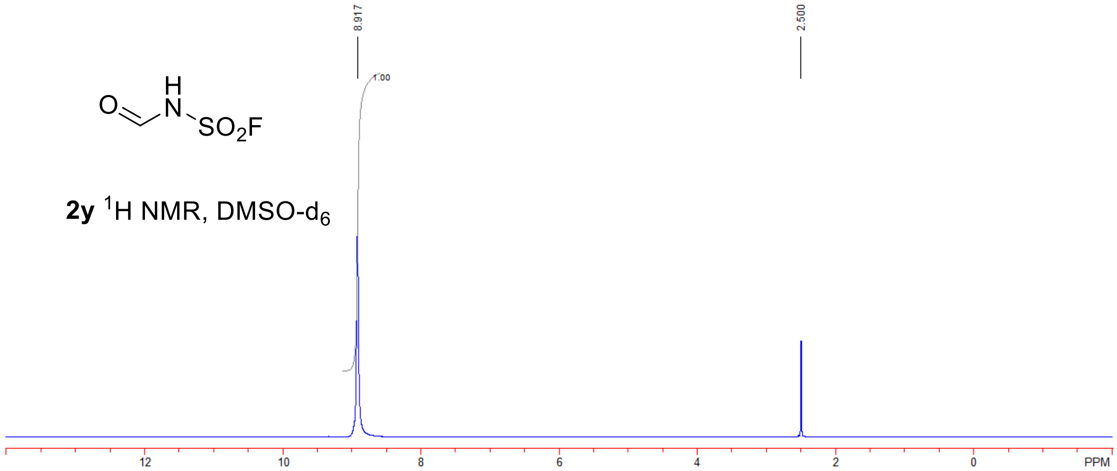
8.917
2.500
1.00
2y 1H NMR, DMSO-d6
12
10
8
6
4
2
0
PPM

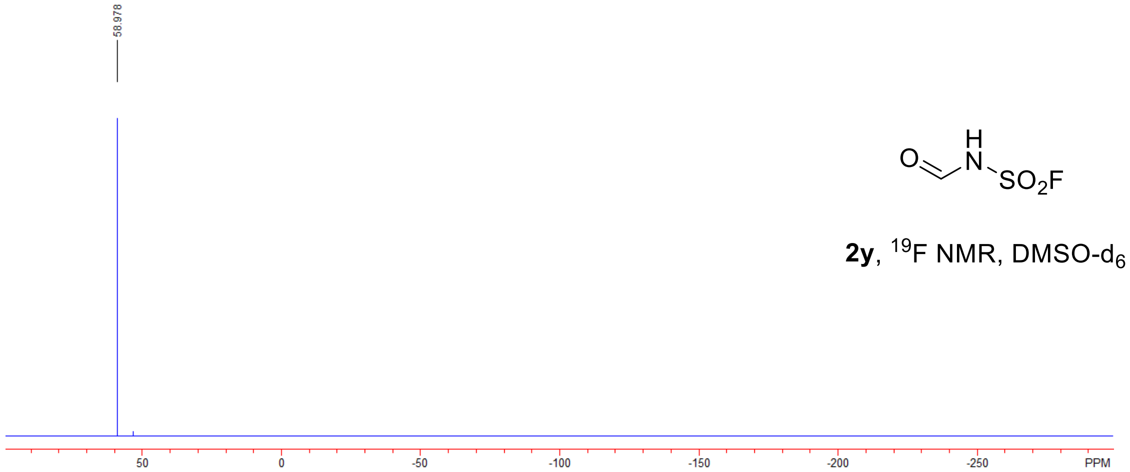
58.978
2y, 19F NMR, DMSO-d6
50
0
-50
-100
-150
-200
-250
PPM

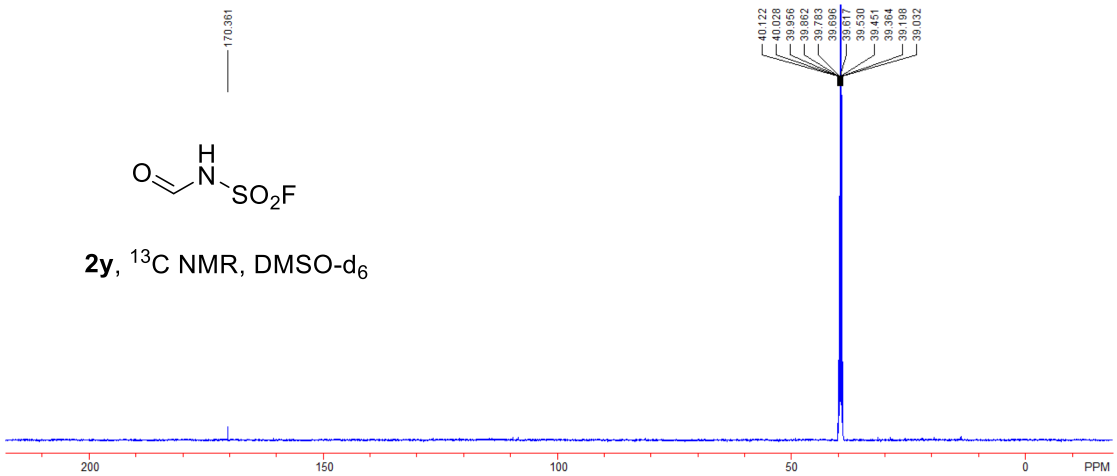
170.361
40.122
40.028
39.956
39.862
39.783
39.696
39.617
39.530
39.451
39.364
39.198
39.032
2y, 13C NMR, DMSO-d6
200
150
100
50
0
PPM

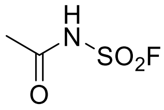

2z

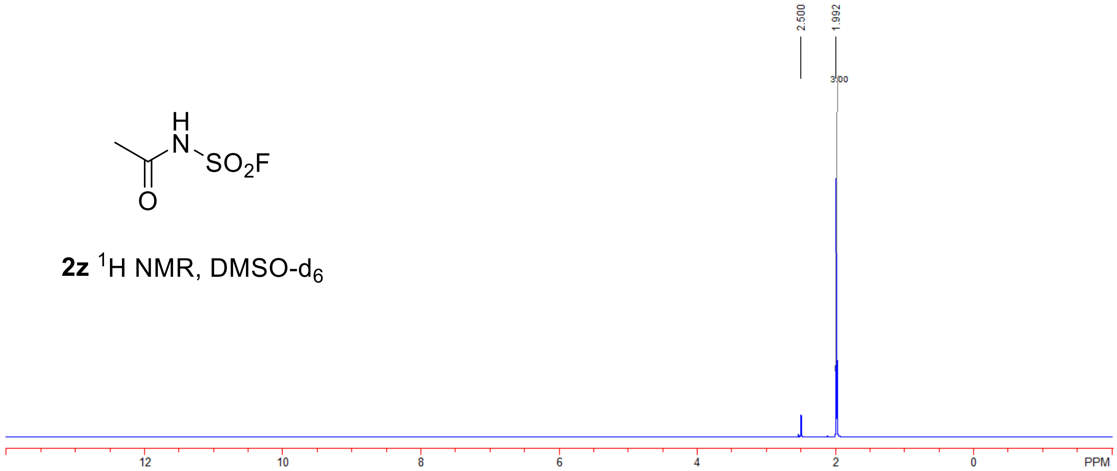
2.500
1.992
3.00
2z 1H NMR, DMSO-d6
12
10
8
6
4
2
0
PPM

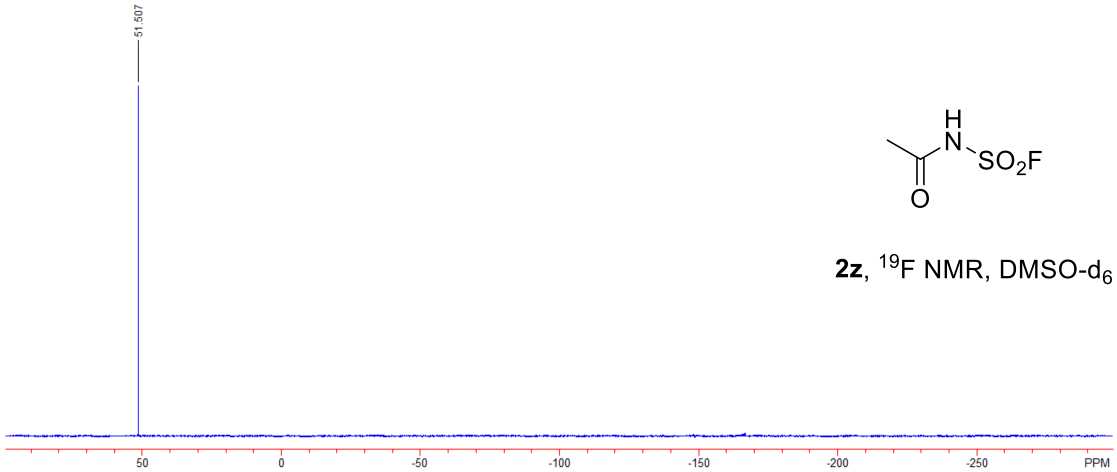
51.507
2z, 19F NMR, DMSO-d6
50
0
-50
-100
-150
-200
-250
PPM

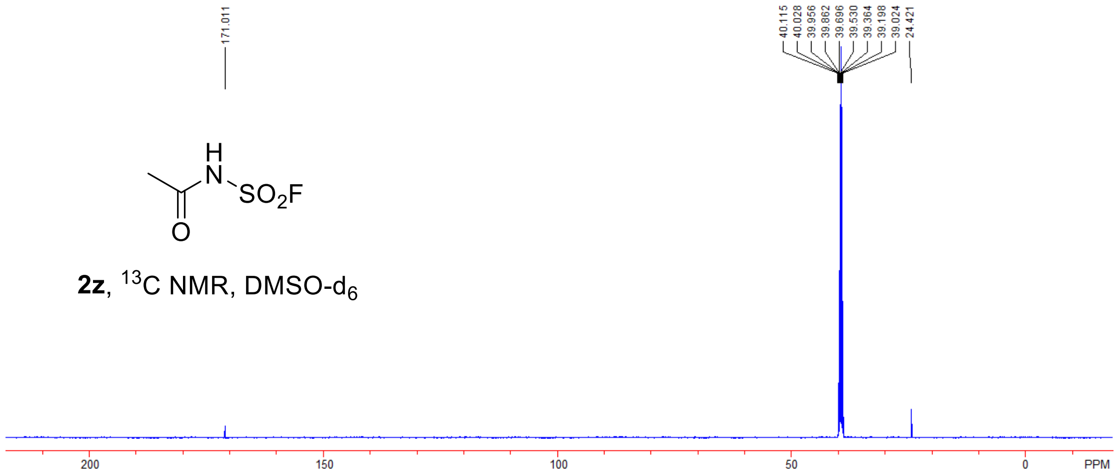
171.011
40.115
40.028
39.956
39.862
39.696
39.530
39.364
39.198
39.024
24.421
2z, 13C NMR, DMSO-d6
200
150
100
50
0
PPM

9. Single crystal data of 4e.

Table 4. Crystal data and structure refinement for 181108c.

Identification code	181108c	
Empirical formula	C7 H8 F N2 Na O7 S	
Formula weight	306.20	
Temperature	293(2) K	
Wavelength	0.71073 A	
Crystal system, space group	Triclinic, P-1	
Unit cell dimensions	a = 5.7233(5) A	alpha = 93.5580(10) deg.
	b = 7.9155(7) A	beta = 96.119(2) deg.
	c = 12.9158(11) A	gamma = 91.6400(10) deg.
Volume	580.30(9) A^3	
Z, Calculated density	2, 1.752 Mg/m^3	
Absorption coefficient	0.362 mm^-1	
F(000)	312	
Crystal size	0.47 x 0.35 x 0.18 mm	
Theta range for data collection	2.58 to 25.00 deg.	
Limiting indices	-6<=h<=6, -9<=k<=9, -15<=l<=12	
Reflections collected / unique	2959 / 2018 [R(int) = 0.0477]	
Completeness to theta = 25.00	98.4 %	
Absorption correction	Semi-empirical from equivalents	
Max. and min. transmission	0.9376 and 0.8481	
Refinement method	Full-matrix least-squares on F^2	
Data / restraints / parameters	2018 / 0 / 172	
Goodness-of-fit on F^2	1.106	
Final R indices [I>2sigma(I)]	R1 = 0.0782, wR2 = 0.1947	
R indices (all data)	R1 = 0.0929, wR2 = 0.2058	
Largest diff. peak and hole	0.595 and -0.616 e.A^-3	

Table 5. Atomic coordinates (x 10^4) and equivalent isotropic displacement parameters (A^2 x 10^3) for 181108c.
U(eq) is defined as one third of the trace of the orthogonalized Uij tensor.

	x	y	z	U(eq)
Na(1)	7313(3)	8827(2)	4447(1)	45(1)
F(1)	2803(5)	4668(4)	4095(2)	66(1)
N(1)	1721(6)	5657(5)	2329(3)	44(1)
N(2)	6653(7)	8298(5)	-1527(3)	51(1)
O(1)	-96(6)	6673(4)	3955(3)	58(1)
O(2)	-859(7)	3811(5)	3164(3)	67(1)
O(3)	4261(5)	7789(4)	3147(2)	52(1)
O(4)	5341(7)	7984(6)	-2323(3)	80(1)
O(5)	8572(7)	8957(5)	-1527(3)	73(1)
O(6)	10555(5)	10375(4)	3820(2)	50(1)
O(7)	4097(5)	8327(4)	5452(2)	46(1)
S(1)	674(2)	5267(1)	3358(1)	42(1)
C(1)	3437(7)	6902(5)	2374(3)	40(1)
C(2)	4266(7)	7195(5)	1327(3)	38(1)
C(3)	2945(7)	6645(5)	395(3)	44(1)
C(4)	3687(8)	7005(6)	-552(3)	45(1)
C(5)	5820(7)	7859(5)	-534(3)	40(1)
C(6)	7204(8)	8378(6)	376(3)	45(1)
C(7)	6396(7)	8030(6)	1311(3)	45(1)

Table 6. Bond lengths [A] and angles [deg] for 181108c.

Na(1)-O(3)	2.380(3)
Na(1)-O(1)#1	2.392(3)
Na(1)-O(7)	2.400(3)
Na(1)-O(7)#2	2.415(3)
Na(1)-O(6)	2.432(3)
Na(1)-O(6)#3	2.470(3)
Na(1)-Na(1)#2	3.641(3)
Na(1)-Na(1)#3	3.655(3)
F(1)-S(1)	1.567(3)
N(1)-C(1)	1.365(5)
N(1)-S(1)	1.560(4)
N(2)-O(5)	1.203(5)
N(2)-O(4)	1.215(5)
N(2)-C(5)	1.472(5)
O(1)-S(1)	1.422(3)
O(1)-Na(1)#4	2.392(3)
O(2)-S(1)	1.421(3)
O(3)-C(1)	1.228(5)
O(6)-Na(1)#3	2.470(3)
O(6)-H(6A)	0.8500
O(6)-H(6B)	0.8499
O(7)-Na(1)#2	2.415(3)
O(7)-H(7A)	0.8500
O(7)-H(7B)	0.8500
C(1)-C(2)	1.508(6)
C(2)-C(7)	1.373(6)
C(2)-C(3)	1.391(5)
C(3)-C(4)	1.380(6)
C(3)-H(3)	0.9300
C(4)-C(5)	1.376(6)
C(4)-H(4)	0.9300
C(5)-C(6)	1.379(6)
C(6)-C(7)	1.379(6)
C(6)-H(6)	0.9300
C(7)-H(7)	0.9300
O(3)-Na(1)-O(1)#1	92.37(13)
O(3)-Na(1)-O(7)	77.52(11)
O(1)#1-Na(1)-O(7)	123.41(13)
O(3)-Na(1)-O(7)#2	94.46(12)
O(1)#1-Na(1)-O(7)#2	154.82(13)

O(7)-Na(1)-O(7)#2	81.75(12)
O(3)-Na(1)-O(6)	115.75(12)
O(1)#1-Na(1)-O(6)	75.73(11)
O(7)-Na(1)-O(6)	157.70(12)
O(7)#2-Na(1)-O(6)	79.47(11)
O(3)-Na(1)-O(6)#3	160.29(13)
O(1)#1-Na(1)-O(6)#3	96.48(12)
O(7)-Na(1)-O(6)#3	82.90(11)
O(7)#2-Na(1)-O(6)#3	84.95(11)
O(6)-Na(1)-O(6)#3	83.57(11)
O(3)-Na(1)-Na(1)#2	84.79(10)
O(1)#1-Na(1)-Na(1)#2	164.42(12)
O(7)-Na(1)-Na(1)#2	41.02(8)
O(7)#2-Na(1)-Na(1)#2	40.72(7)
O(6)-Na(1)-Na(1)#2	119.26(11)
O(6)#3-Na(1)-Na(1)#2	81.96(9)
O(3)-Na(1)-Na(1)#3	157.71(11)
O(1)#1-Na(1)-Na(1)#3	84.97(10)
O(7)-Na(1)-Na(1)#3	122.15(10)
O(7)#2-Na(1)-Na(1)#3	79.57(9)
O(6)-Na(1)-Na(1)#3	42.18(8)
O(6)#3-Na(1)-Na(1)#3	41.39(7)
Na(1)#2-Na(1)-Na(1)#3	103.34(8)
C(1)-N(1)-S(1)	118.2(3)
O(5)-N(2)-O(4)	122.3(4)
O(5)-N(2)-C(5)	119.7(4)
O(4)-N(2)-C(5)	117.9(4)
S(1)-O(1)-Na(1)#4	157.2(2)
C(1)-O(3)-Na(1)	154.5(3)
Na(1)-O(6)-Na(1)#3	96.43(11)
Na(1)-O(6)-H(6A)	112.4
Na(1)#3-O(6)-H(6A)	112.6
Na(1)-O(6)-H(6B)	112.4
Na(1)#3-O(6)-H(6B)	112.4
H(6A)-O(6)-H(6B)	110.1
Na(1)-O(7)-Na(1)#2	98.25(11)
Na(1)-O(7)-H(7A)	112.2
Na(1)#2-O(7)-H(7A)	112.1
Na(1)-O(7)-H(7B)	112.1
Na(1)#2-O(7)-H(7B)	112.1
H(7A)-O(7)-H(7B)	109.8
O(2)-S(1)-O(1)	117.8(2)
O(2)-S(1)-N(1)	109.0(2)
O(1)-S(1)-N(1)	116.6(2)

O(2)-S(1)-F(1)	104.3(2)
O(1)-S(1)-F(1)	102.28(18)
N(1)-S(1)-F(1)	104.93(18)
O(3)-C(1)-N(1)	127.0(4)
O(3)-C(1)-C(2)	119.6(4)
N(1)-C(1)-C(2)	113.4(3)
C(7)-C(2)-C(3)	119.9(4)
C(7)-C(2)-C(1)	118.1(3)
C(3)-C(2)-C(1)	122.0(4)
C(4)-C(3)-C(2)	120.8(4)
C(4)-C(3)-H(3)	119.6
C(2)-C(3)-H(3)	119.6
C(5)-C(4)-C(3)	117.4(4)
C(5)-C(4)-H(4)	121.3
C(3)-C(4)-H(4)	121.3
C(4)-C(5)-C(6)	123.2(4)
C(4)-C(5)-N(2)	119.1(4)
C(6)-C(5)-N(2)	117.7(4)
C(7)-C(6)-C(5)	118.1(4)
C(7)-C(6)-H(6)	120.9
C(5)-C(6)-H(6)	120.9
C(2)-C(7)-C(6)	120.5(4)
C(2)-C(7)-H(7)	119.7
C(6)-C(7)-H(7)	119.7

Symmetry transformations used to generate equivalent atoms:

#1 x+1,y,z #2 -x+1,-y+2,-z+1 #3 -x+2,-y+2,-z+1

#4 x-1,y,z

Table 7. Anisotropic displacement parameters (A^2 x 10^3) for 181108c.
The anisotropic displacement factor exponent takes the form:
-2 pi^2 [h^2 a*^2 U11 + ... + 2 h k a* b* U12]

	U11	U22	U33	U23	U13	U12
Na(1)	40(1)	46(1)	48(1)	2(1)	5(1)	2(1)
F(1)	70(2)	73(2)	58(2)	27(1)	-4(1)	20(2)
N(1)	47(2)	45(2)	40(2)	6(2)	2(2)	-10(2)
N(2)	60(2)	51(2)	44(2)	9(2)	6(2)	7(2)
O(1)	61(2)	47(2)	70(2)	8(2)	24(2)	9(2)
O(2)	77(2)	59(2)	65(2)	5(2)	12(2)	-27(2)
O(3)	54(2)	63(2)	37(2)	0(1)	1(1)	-17(2)
O(4)	89(3)	114(3)	37(2)	17(2)	1(2)	-6(2)
O(5)	63(2)	95(3)	61(2)	12(2)	17(2)	-19(2)
O(6)	49(2)	46(2)	58(2)	16(1)	11(1)	1(1)
O(7)	49(2)	46(2)	45(2)	11(1)	2(1)	1(1)
S(1)	46(1)	36(1)	46(1)	9(1)	5(1)	0(1)
C(1)	36(2)	41(2)	42(2)	5(2)	1(2)	4(2)
C(2)	39(2)	33(2)	41(2)	5(2)	-1(2)	3(2)
C(3)	39(2)	45(3)	48(2)	9(2)	3(2)	-2(2)
C(4)	49(2)	48(3)	37(2)	1(2)	-4(2)	1(2)
C(5)	44(2)	40(2)	37(2)	10(2)	6(2)	6(2)
C(6)	42(2)	47(3)	48(2)	11(2)	4(2)	-5(2)
C(7)	39(2)	55(3)	38(2)	5(2)	-3(2)	-2(2)

Table 8. Hydrogen coordinates (x 10^4) and isotropic displacement parameters (A^2 x 10^3) for 181108c.

	x	y	z	U(eq)
H(6A)	10103	11230	3496	60
H(6B)	11349	9747	3441	60
H(7A)	4555	7994	6051	56
H(7B)	3072	7625	5135	56
H(3)	1543	6026	409	53
H(4)	2781	6683	-1178	55
H(6)	8643	8947	360	55
H(7)	7300	8364	1936	54

Table 9. Torsion angles [deg] for 181108c.

O(1)#1-Na(1)-O(3)-C(1)	-10.7(7)
O(7)-Na(1)-O(3)-C(1)	-134.4(7)
O(7)#2-Na(1)-O(3)-C(1)	145.1(7)
O(6)-Na(1)-O(3)-C(1)	64.6(7)
O(6)#3-Na(1)-O(3)-C(1)	-127.4(7)
Na(1)#2-Na(1)-O(3)-C(1)	-175.3(7)
Na(1)#3-Na(1)-O(3)-C(1)	71.8(8)
O(3)-Na(1)-O(6)-Na(1)#3	175.93(14)
O(1)#1-Na(1)-O(6)-Na(1)#3	-98.39(13)
O(7)-Na(1)-O(6)-Na(1)#3	53.0(3)
O(7)#2-Na(1)-O(6)-Na(1)#3	86.03(12)
O(6)#3-Na(1)-O(6)-Na(1)#3	0.0
Na(1)#2-Na(1)-O(6)-Na(1)#3	77.03(12)
O(3)-Na(1)-O(7)-Na(1)#2	-96.47(13)
O(1)#1-Na(1)-O(7)-Na(1)#2	179.00(14)
O(7)#2-Na(1)-O(7)-Na(1)#2	0.0
O(6)-Na(1)-O(7)-Na(1)#2	32.8(3)
O(6)#3-Na(1)-O(7)-Na(1)#2	85.90(12)
Na(1)#3-Na(1)-O(7)-Na(1)#2	72.11(13)
Na(1)#4-O(1)-S(1)-O(2)	-51.3(6)
Na(1)#4-O(1)-S(1)-N(1)	81.2(5)
Na(1)#4-O(1)-S(1)-F(1)	-165.0(5)
C(1)-N(1)-S(1)-O(2)	-173.1(3)
C(1)-N(1)-S(1)-O(1)	50.5(4)
C(1)-N(1)-S(1)-F(1)	-61.8(4)
Na(1)-O(3)-C(1)-N(1)	116.1(6)
Na(1)-O(3)-C(1)-C(2)	-66.4(8)
S(1)-N(1)-C(1)-O(3)	-0.9(6)
S(1)-N(1)-C(1)-C(2)	-178.5(3)
O(3)-C(1)-C(2)-C(7)	20.8(6)
N(1)-C(1)-C(2)-C(7)	-161.3(4)
O(3)-C(1)-C(2)-C(3)	-159.3(4)
N(1)-C(1)-C(2)-C(3)	18.6(6)
C(7)-C(2)-C(3)-C(4)	-3.4(6)
C(1)-C(2)-C(3)-C(4)	176.7(4)
C(2)-C(3)-C(4)-C(5)	2.7(6)
C(3)-C(4)-C(5)-C(6)	-0.8(7)
C(3)-C(4)-C(5)-N(2)	-178.8(4)
O(5)-N(2)-C(5)-C(4)	-175.7(4)
O(4)-N(2)-C(5)-C(4)	4.8(6)
O(5)-N(2)-C(5)-C(6)	6.2(6)

O(4)-N(2)-C(5)-C(6)	-173.2(4)
C(4)-C(5)-C(6)-C(7)	-0.4(7)
N(2)-C(5)-C(6)-C(7)	177.6(4)
C(3)-C(2)-C(7)-C(6)	2.1(7)
C(1)-C(2)-C(7)-C(6)	-178.0(4)
C(5)-C(6)-C(7)-C(2)	-0.2(7)

Symmetry transformations used to generate equivalent atoms:
#1 x+1,y,z #2 -x+1,-y+2,-z+1 #3 -x+2,-y+2,-z+1
#4 x-1,y,z

Table 10. Hydrogen bonds for 181108c [A and deg.].

D-H	d(D-H)	d(H..A)	<DHA	d(D..A)	A
O6-H6A	0.850	2.187	161.73	3.006	O2 [x+1, y+1, z]
O6-H6B	0.850	2.353	154.45	3.141	O3 [x+1, y, z]
O7-H7A	0.850	2.101	160.82	2.917	O4 [x, y, z+1]
O7-H7A	0.850	2.637	111.27	3.050	F1 [-x+1, -y+1, -z+1]
O7-H7B	0.850	2.317	157.46	3.119	O1
O7-H7B	0.850	2.618	139.96	3.314	F1
