# Supplementary material for: Installation of -SO2F groups onto primary amides
Source: Beilstein J Org Chem. 2019 Aug 9;15:1907–12. doi: 10.3762/bjoc.15.186 (PMC6693406; doi:10.3762/bjoc.15.186)

BEILSTEIN JOURNAL OF ORGANIC CHEMISTRY

Supporting Information

for

Installation of -SO_2F groups onto primary amides

Jing Liu, Shi-Meng Wang, Njud S. Alharbi and Hua-Li Qin

Beilstein J. Org. Chem. **2019,** *15,* 1907–1912. doi:10.3762/bjoc.15.186

Checkcif file of 4e

License and Terms: This is a supporting information file under the terms of the Creative Commons Attribution License (http://creativecommons.org/licenses/by/4.0). Please note that the reuse, redistribution and reproduction in particular requires that the authors and source are credited.
The license is subject to the *Beilstein Journal of Organic Chemistry* terms and conditions: (https://www.beilstein-journals.org/bjoc)

checkCIF/PLATON report

Structure factors have been supplied for datablock(s) 181108c

THIS REPORT IS FOR GUIDANCE ONLY. IF USED AS PART OF A REVIEW PROCEDURE FOR PUBLICATION, IT SHOULD NOT REPLACE THE EXPERTISE OF AN EXPERIENCED CRYSTALLOGRAPHIC REFEREE.

No syntax errors found. CIF dictionary Interpreting this report

Datablock: 181108c

```
Bond precision:  C-C = 0.0059 A          Wavelength=0.71073

Cell:           a=5.7233(5)        b=7.9155(7)       c=12.9158(11)
                alpha=93.558(1)    beta=96.119(2)    gamma=91.640(1)
Temperature:    293 K

                 Calculated                 Reported
Volume           580.30(9)                  580.30(9)
Space group      P -1                       P-1
Hall group       -P 1                       ?
Moiety formula   C7 H8 F N2 Na O7 S         ?
Sum formula      C7 H8 F N2 Na O7 S         C7 H8 F N2 Na O7 S
Mr               306.20                     306.20
Dx,g cm-3        1.752                      1.752
Z                2                          2
Mu (mm-1)        0.362                      0.362
F000             312.0                      312.0
F000'            312.52
h,k,lmax         6,9,15                     6,9,15
Nref             2050                       2018
Tmin,Tmax        0.859,0.937                0.848,0.938
Tmin'            0.844

Correction method= # Reported T Limits: Tmin=0.848 Tmax=0.938
AbsCorr = MULTI-SCAN

Data completeness= 0.984          Theta(max)= 25.000

R(reflections)= 0.0782( 1488)     wR2(reflections)= 0.2058( 2018)

S = 1.106                 Npar= 172
```

The following ALERTS were generated. Each ALERT has the format
test-name_ALERT_alert-type_alert-level.
Click on the hyperlinks for more details of the test.

Alert level A

```
EXPT005_ALERT_1_A  _exptl_crystal_description is missing
            Crystal habit description.
            The following tests will not be performed.
            CRYSR_01
PLAT902_ALERT_1_A No (Interpretable) Reflections Found in FCF ....     Please Check
```

Alert level C

```
PLAT242_ALERT_2_C Low    'MainMol' Ueq as Compared to Neighbors of         S1 Check
PLAT340_ALERT_3_C Low Bond Precision on  C-C Bonds ...............    0.00586 Ang.
```

Alert level G

```
PLAT004_ALERT_5_G Polymeric Structure Found with Maximum Dimension          1 Info
PLAT005_ALERT_5_G No Embedded Refinement Details Found  in the CIF     Please Do !
PLAT007_ALERT_5_G Number of Unrefined Donor-H Atoms ..............          4 Report
PLAT066_ALERT_1_G Predicted and Reported Tmin&Tmax Range Identical          ? Check
PLAT072_ALERT_2_G SHELXL First  Parameter in WGHT  Unusually Large       0.12 Report
PLAT093_ALERT_1_G No s.u.'s on H-positions, Refinement Reported as      mixed Check
PLAT199_ALERT_1_G Reported _cell_measurement_temperature ..... (K)        293 Check
PLAT200_ALERT_1_G Reported   _diffrn_ambient_temperature ..... (K)        293 Check
PLAT764_ALERT_4_G Overcomplete CIF Bond List Detected (Rep/Expd) .       1.23 Ratio
PLAT899_ALERT_4_G SHELXL97   is Deprecated and Succeeded by SHELXL       2018 Note
```

2 **ALERT level A** = Most likely a serious problem - resolve or explain
0 **ALERT level B** = A potentially serious problem, consider carefully
2 **ALERT level C** = Check. Ensure it is not caused by an omission or oversight
10 **ALERT level G** = General information/check it is not something unexpected

6 ALERT type 1 CIF construction/syntax error, inconsistent or missing data
2 ALERT type 2 Indicator that the structure model may be wrong or deficient
1 ALERT type 3 Indicator that the structure quality may be low
2 ALERT type 4 Improvement, methodology, query or suggestion
3 ALERT type 5 Informative message, check

It is advisable to attempt to resolve as many as possible of the alerts in all categories. Often the minor alerts point to easily fixed oversights, errors and omissions in your CIF or refinement strategy, so attention to these fine details can be worthwhile. In order to resolve some of the more serious problems it may be necessary to carry out additional measurements or structure refinements. However, the purpose of your study may justify the reported deviations and the more serious of these should normally be commented upon in the discussion or experimental section of a paper or in the "special_details" fields of the CIF. checkCIF was carefully designed to identify outliers and unusual parameters, but every test has its limitations and alerts that are not important in a particular case may appear. Conversely, the absence of alerts does not guarantee there are no aspects of the results needing attention. It is up to the individual to critically assess their own results and, if necessary, seek expert advice.

Publication of your CIF in IUCr journals

A basic structural check has been run on your CIF. These basic checks will be run on all CIFs submitted for publication in IUCr journals (*Acta Crystallographica*, *Journal of Applied Crystallography*, *Journal of Synchrotron Radiation*); however, if you intend to submit to *Acta Crystallographica Section C* or *E* or *IUCrData*, you should make sure that full publication checks are run on the final version of your CIF prior to submission.

Publication of your CIF in other journals

Please refer to the *Notes for Authors* of the relevant journal for any special instructions relating to CIF submission.

Validation response form

Please find below a validation response form (VRF) that can be filled in and pasted into your CIF.

```
# start Validation Reply Form
_vrf_EXPT005_181108c
;
PROBLEM: _exptl_crystal_description is missing
RESPONSE: ...
;
_vrf_PLAT902_181108c
;
PROBLEM: No (Interpretable) Reflections Found in FCF ....     Please Check
RESPONSE: ...
;
_vrf_PLAT242_181108c
;
PROBLEM: Low    'MainMol' Ueq as Compared to Neighbors of         S1 Check
RESPONSE: ...
;
_vrf_PLAT340_181108c
;
PROBLEM: Low Bond Precision on  C-C Bonds ..............    0.00586 Ang.
RESPONSE: ...
;
# end Validation Reply Form
```

PLATON version of 17/03/2019; check.def file version of 04/03/2019

Datablock 181108c - ellipsoid plot

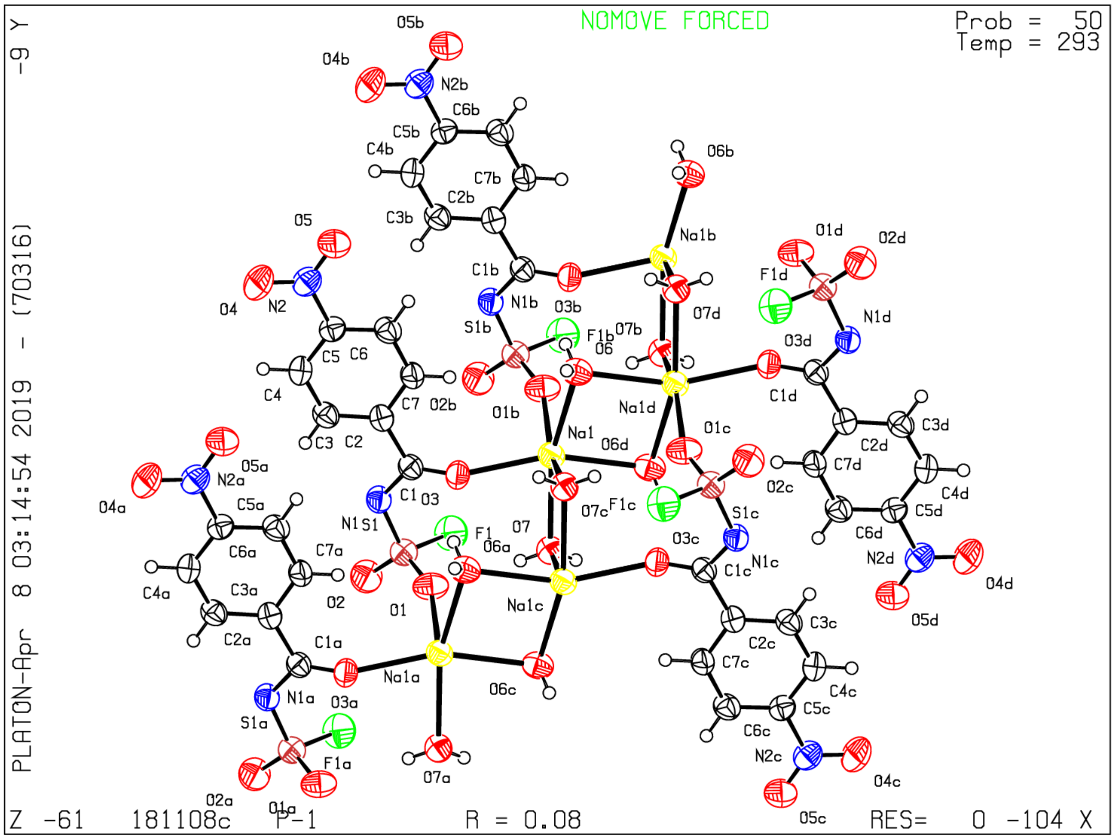

Supplement: File 3 — Checkcif file of 4e. [file Beilstein_J_Org_Chem-15-1907-s003.pdf]
